# Global burden, trends, and inequalities in cancer and subtypes attributable to high BMI among older adults, 1990–2021: a secondary analysis of the global burden of disease study 2021

**DOI:** 10.3389/fnut.2025.1683893

**Published:** 2025-11-11

**Authors:** Yitong Huang, Di Qiu, Feng Xuan

**Affiliations:** 1 Department of Internal Medicine, Zhuji Maternal and Child Health Hospital, Shaoxing, Zhejiang, China; 2 Department of Hematology, Zhuji Affiliated Hospital of Wenzhou Medical University, Shaoxing, China; 3 Department of Radiation Oncology, Zhuji Affiliated Hospital of Wenzhou Medical University, Shaoxing, Zhejiang, China

**Keywords:** global burden of disease, cancer, disability-adjusted life years, high body mass index, older adults, health inequalities, joinpoint regression model

## Abstract

**Background:**

High body mass index (BMI) is a significant modifiable risk factor for cancer, yet its global burden in older adults (aged ≥60 years) remains incompletely understood.

**Methods:**

Using Global Burden of Disease Study 2021 data, we estimated disability-adjusted life-years (DALYs) and age-standardized DALY rates (ASDRs) for total and 11 high BMI-related cancers from 1990 to 2021. Trends were assessed using average annual percentage change. Inequalities were measured using the slope index of inequality and concentration index. Additionally, decomposition and frontier analyses were utilized to examine driving factors and explore improvement potential.

**Results:**

Global cancer-related DALYs attributable to high BMI in older adults increased from 2.11 million in 1990 to 5.40 million in 2021, with ASDR rising from 439.99 to 497.15 per 100,000 population. Males showed greater increases despite lower ASDR. From 1990 to 2021, Asia and Africa experienced the steepest increases, while Europe and America recorded the highest ASDRs in 2021. High Socio-demographic Index (SDI) regions consistently reported the highest ASDRs in both 1990 and 2021, whereas low-middle SDI regions exhibited the most rapid increase during this period. The global ASDRs for most cancers increased from 1990 to 2021, with colorectal cancer demonstrating the highest ASDR among men and breast cancer among women in 2021. DALY growth was largely attributed to population expansion (133.04%) through decomposition analysis. Inequalities have narrowed but remained predominantly concentrated in higher SDI countries. Frontier analysis identified several high SDI countries, such as the United Arab Emirates, Slovakia, and Qatar, with substantial potential for burden reduction.

**Conclusion:**

The cancer burden attributable to high BMI in older adults has risen substantially since 1990, with marked geographic, socioeconomic, and cancer-site disparities. Targeted high BMI reduction strategies in aging populations are critical to mitigating future cancer burden and reducing inequalities.

## 1 Introduction

Cancer constitutes a significant and growing global public health challenge, ranking among the leading causes of mortality and disability worldwide (1). The roughly 10 million cancer-related deaths in 2022 underscore the increasing burden on health systems, particularly in the context of population changes such as aging (2). Age is a major risk factor for cancer, with the incidence and mortality rates rising markedly after the age of 60, largely due to cumulative exposure to carcinogens, immune senescence, and increased prevalence of comorbidities (3, 4). It is estimated that by 2050, the global population of older adults will exceed 2 billion, making up more than 20% of the global population (5). This demographic change is likely to drive an increasing proportion of the global cancer burden among older individuals. Therefore, understanding the cancer burden in this population, especially that related to modifiable risk factors, is crucial for developing effective public health interventions.

High body mass index (BMI), a measure of overweight and obesity, is a well-documented modifiable risk factor for various chronic diseases, including cancer (6–8). The global prevalence of obesity has increased significantly, with the age-standardized prevalence in men rising from 3.2% in 1975 to 10.8% in 2014, and in women from 6.4% to 14.9% (9). Additionally, over 640 million adults were classified as obese in 2014 (9). This trend is particularly concerning for older adults, who are more likely to have excess body weight and face heightened health risks associated with it (10–12). Furthermore, high BMI has been linked to an increased risk of various cancers, such as breast, stomach, colorectal, liver, gallbladder, thyroid, and kidney cancers (7, 8, 13). The mechanisms underlying this association are multifaceted, involving chronic inflammation, insulin resistance, and alterations in sex hormone levels, all of which are exacerbated by excess body fat (14–16).

Despite the established link between high BMI and cancer risk, previous research has primarily focused on global cancer burden across all age groups or on specific cancer subtypes. For instance, a 2019 study estimated deaths from cancers attributable to high BMI increased by 35% between 2010 and 2019 (17). Similarly, recent reports have examined high BMI attributable breast cancer, colorectal cancer and liver cancer (18–20), but these studies were limited to single cancer types. A comprehensive population-level analysis assessing the burden and temporal trends of high BMI attributable cancers in older adults, especially across various cancer subtypes, remains a gap in current research. This is particularly critical given the demographic shift toward an aging population and the higher prevalence of both cancer and obesity in this group.

To address these gaps, we performed a comprehensive secondary analysis of the latest 2021 Global Burden of Disease (GBD) study data, assessing the cancer burden, temporal trends, and inequalities attributable to high BMI among adults aged 60 years and older. We focus on disability-adjusted life-years (DALYs) associated with total cancer and 11 specific cancer types that have been identified as causally or potentially related to high BMI: breast, ovarian, uterine, colon and rectum, gallbladder and biliary tract, liver, multiple myeloma, non-Hodgkin lymphoma, leukemia, kidney, and thyroid cancers. Additionally, we estimated the burden across multiple spatial and sociodemographic levels, including global, continental (Africa, Americas, Asia, Europe), GBD regions, 204 countries and territories, and Socio-demographic Index (SDI) levels. These findings might inform public health strategies focused on aging populations, prioritize cancer prevention efforts targeting modifiable risk factors, and support policies aimed at mitigating the impact of the obesity epidemic on aging societies. Collectively, these measures could promote healthy aging, reduce cancer-related DALYs, and enhance global equity in cancer outcomes.

## 2 Methods

### 2.1 Data sources

We used data from the GBD Study 2021, coordinated by the Institute for Health Metrics and Evaluation, which provides systematic and comparable estimates of disease burden across 204 countries and territories from 1990 to 2021. The GBD 2021 database includes age- and sex-specific estimates for 371 diseases and injuries, and 288 causes of death, as well as comparative risks for 88 risk factors (21–23). In this study, we selected specific parameters: Risk factor under GBD Estimate; DALYs under Measure; and number and rate under Metric. High BMI was chosen as the risk factor. The causes included neoplasms, breast, ovarian, uterine, colorectal, gallbladder and biliary tract, liver, multiple myeloma, non-Hodgkin lymphoma, leukemia, kidney, and thyroid cancers. All data used can be accessed publicly through the Global Health Data Exchange online platform at https://ghdx.healthdata.org/gbd-results-tool. Further methodological details and the comparative risk assessment specifically for high BMI are publicly available through the GBD 2021 data repository (https://ghdx.healthdata.org/gbd-2021) and have described in prior publications (21–24).

### 2.2 Definitions

In this study, we defined older adults as individuals aged 60 years or older, consistent with the age group stratification recommended by the World Health Organization (WHO) and used in previous GBD studies (5, 25, 26). DALYs were calculated as the sum of YLLs due to premature mortality and YLDs, thereby reflecting the overall health loss from disease or injury. High BMI was considered a key metabolic risk factor in this study and defined according to GBD 2021 as a BMI greater than 25 kg/m^2^ (27), following WHO guidelines for overweight and obesity.

The SDI, developed by the Institute for Health Metrics and Evaluation, was used to assess country-level development status (28). It is a composite indicator that integrates three dimensions of socio-economic development: (1) total fertility rate under age 25, (2) mean educational attainment in the population aged 15 years and older, and (3) lag-distributed income per capita. Two hundred four countries and territories were grouped into five SDI quintiles (low, low-middle, middle, high-middle, and high) based on their 2021 SDI values (Supplementary Tables S1, S2).

Cancer in this study was categorized to include total cancer (neoplasms) and 11 specific site-level subtypes as defined in the GBD 2021 cause list. The International Classification of Diseases codes used to define each cancer type is provided in Supplementary Table S3 (21).

### 2.3 Statistical analysis

Subgroups were analyzed across age groups (60–64, 65–69, 70–74, 75–79, 80–84, 85–89, 90–94, and 95+ years), sex, and multiple geographical levels, including global, continental (Africa, Americas, Asia, Europe), SDI levels, GBD regions, and 204 countries/territories.

#### 2.3.1 Age-standardized DALYs rates

Age-standardized DALYs rates (ASDRs) for cancer attributable to high BMI among adults aged 60 years and older were calculated using the direct standardization method (29). ASDRs were expressed per 100,000 population and computed using the following formula:


$$\begin{array}{l} \frac{\sum_{i=1}^{N}\alpha_{i}w_{i}}{\sum_{i=1}^{N}w_{i}} \end{array}$$ (1)

where *a*_*i*_ denotes the age-specific rate in the *i*th 5-year age group and *w*_*i*_ is the corresponding age-group weight from the GBD global reference population (21). N, in this study, was 8, representing the total number of age groups. This method enables comparison of disease burden across populations by adjusting for differences in age structure.

#### 2.3.2 Joinpoint regression analysis

Joinpoint regression analysis was conducted to examine temporal trends in cancer and its subtypes attributable to high BMI from 1990 to 2021. This segmented regression technique identifies inflection points (“joinpoints”) where statistically significant shifts in trend occur. ASDRs were analyzed using the Joinpoint Regression Program (30). Annual percentage change (APC) and average annual percentage change (AAPC) were computed for each segment and across the full interval. The Monte Carlo permutation method (4,499 permutations) was applied to assess the significance of identified joinpoints. A trend was defined as increasing or decreasing if the APC or AAPC and its 95% confidence interval (CI) were entirely above or below zero, respectively. Non-significant trends were identified if the 95% CI included zero.

#### 2.3.3 Decomposition analysis

To identify the underlying drivers of the observed changes in DALYs from 1990 to 2021, we conducted a decomposition analysis based on the Das Gupta method (31, 32), which partitions the net change in total DALYs into three additive components: population growth, population aging, and epidemiological changes. Here, “epidemiological changes” refer to shifts in age-specific DALY rates that may arise from evolving incidence, treatment outcomes, survival, healthcare accessibility, and public health interventions. The decomposition was conducted for both sexes and across global, continental, SDI regional, and GBD regional levels. This approach has been widely adopted in GBD-based research to provide quantitative insights into the demographic and epidemiologic transitions that shape disease burden across time and regions (32, 33).

#### 2.3.4 Cross-country inequality analysis

To quantify global disparities in ASDR of high BMI-attributable cancers across 204 countries and territories, we employed two established inequality metrics recommended by the WHO: The Slope Index of Inequality (SII) and the Concentration Index (CIX) (34). The SII measures absolute inequality by regressing ASDR on a relative rank based on each country's SDI, defined by the midpoint of its cumulative population distribution. A positive SII indicates that ASDRs are concentrated among higher SDI countries, whereas a negative SII implies higher burdens in lower SDI countries. Relative inequality was assessed using the CIX, calculated by numerically integrating the area under the Lorenz curve that plots the cumulative proportion of ASDR against the cumulative population share ranked by SDI. The CIX ranges from −1 to +1; values closer to zero denote equality, while positive (or negative) values indicate disproportionate burden among higher- (or lower-) SDI countries, respectively. Both indices were calculated using robust regression models to account for heteroscedasticity and to ensure stability against outliers. This dual-metric approach enables a nuanced assessment of the magnitude and direction of global inequality in ASDR of high BMI–related cancer among older adults.

#### 2.3.5 Frontier analysis

To assess the minimum achievable burden of cancer attributable to high BMI across varying levels of socio-economic development, we performed frontier analysis incorporating the Free Disposal Hull (FDH) model and locally estimated scatterplot smoothing (LOESS) (35). Based on GBD 2021 data from 204 countries and territories, we performed 100 bootstrap iterations to estimate average ASDRs at each SDI value. A nonparametric frontier curve was generated using LOESS (local polynomial degree = 1, span = 0.2) to capture the nonlinear relationship between ASDR and SDI. The vertical distance between each country's observed ASDR and the estimated frontier, termed the “effective difference,” quantified the gap between actual and optimal performance. Countries achieving frontier-level performance were assigned an effective difference of zero. This approach enabled identification of countries with unrealized health potential at similar levels of socio-economic development, highlighting regions with the greatest opportunities for targeted intervention and resource optimization.

Statistical analyses were conducted utilizing R software (version 4.4.1; The R Foundation for Statistical Computing, Vienna, Austria) for data processing and visualization. Joinpoint regression analysis was performed using Joinpoint software (version 5.1.0; National Cancer Institute, Bethesda, MD, USA). Figures were generated with the ggplot2 package.

## 3 Results

### 3.1 Burden of total cancer attributable to high BMI among older people

#### 3.1.1 At global levels

**Figure 1:**
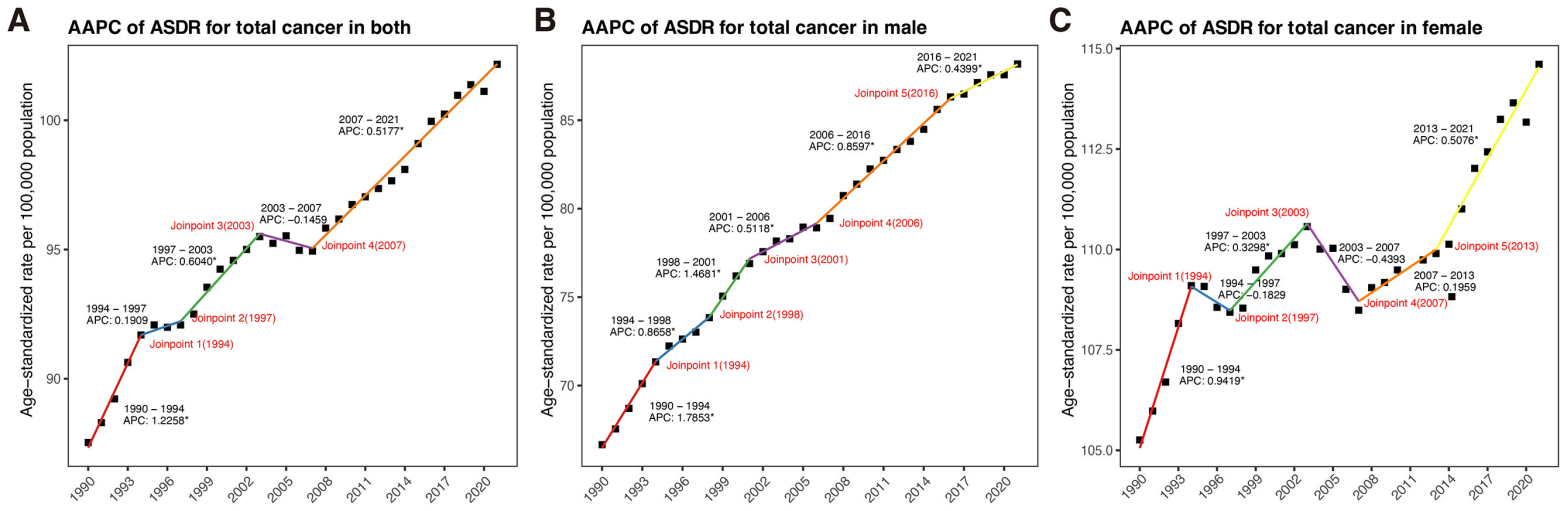
Joinpoint regression analysis of global ASDR for total cancer attributable to high BMI among older people from 1990 to 2021, by sex. **(A)** In both sexes. **(B)** In male. **(C)** In female. DALYs, disability-adjusted life years; ASDR, age-standardized DALYs rate; AAPC, average annual percent change.

In 2021, the global number of cancer-related DALYs attributable to high BMI among older adults reached 5.40 million, increasing by more than 2.5-fold from 2.11 million in 1990 (Table 1). The global ASDR increased modestly from 439.99 per 100,000 population (95% CI: 177.29–724.52) in 1990 to 497.15 per 100,000 population (95% CI: 197.77–813.05) in 2021, with an AAPC of 0.51% (95% CI: 0.40–0.62) (Table 1, Figure 1A). Males experienced a more pronounced increase in ASDR, with an AAPC of 0.91% (95% CI: 0.81–1.02) (Table 1, Figure 1B). In contrast, females had higher ASDRs across both years but a slower increase over time (AAPC: 0.28%, 95% CI: 0.16–0.40) (Table 1, Figure 1C).

**Table 1:** DALYs and ASDR for total cancer attributable to high BMI among older people in 1990 and 2021, with AAPC of ASDR from 1990 to 2021.

**Location**	**DALYs in 1990 (95%CI)**	**DALYs in 2021 (95%CI)**	**ASDR in 1990 (per 100,000 population, 95%CI)**	**ASDR in 2021 (per 100,000 population, 95%CI)**	**AAPC of ASDR (95%CI)**
Global	2,106,306 (850,646 to 3,464,747)	5,398,720 (2,150,802 to 8,822,883)	439.99 (177.29 to 724.52)	497.15 (197.77 to 813.05)	0.51 (0.40 to 0.62)
Male	674,837 (304,835 to 1,100,145)	2,043,909 (882,175 to 3,321,092)	317.63 (143.93 to 516.74)	411.60 (177.82 to 668.72)	0.91 (0.81 to 1.02)
Female	1,431,469 (548,330 to 2,379,743)	3,354,811 (1,258,005 to 5,541,201)	537.07 (205.41 to 893.51)	571.74 (214.22 to 944.29)	0.28 (0.16 to 0.40)
Asia	346,110 (167,848 to 552,443)	1,700,997 (707,180 to 2,763,100)	145.69 (70.88 to 232.01)	270.00 (112.35 to 438.63)	1.85 (1.77 to 1.93)
America	585,379 (219,787 to 976,001)	1,439,257 (572,914 to 2,374,024)	753.22 (282.66 to 1,256.36)	819.46 (326.06 to 1,351.61)	0.36 (0.27 to 0.45)
Europe	1,099,516 (435,642 to 1,817,419)	1,916,666 (740,491 to 3,195,552)	803.83 (318.03 to 1,329.19)	910.96 (352.46 to 1,517.28)	0.24 (0.05 to 0.43)
Africa	68,365 (26,184 to 111,419)	325,975 (120,380 to 544,055)	207.94 (79.21 to 339.71)	441.35 (163.15 to 736.77)	2.20 (2.06 to 2.34)
High SDI	1,095,387 (417,054 to 1,824,885)	2,088,693 (806,778 to 3,457,569)	758.03 (288.18 to 1,263.11)	753.40 (291.41 to 1,244.91)	−0.02 (−0.17 to 0.14)
High-middle SDI	681,967 (285,637 to 1,116,198)	1,638,810 (653,959 to 2,701,854)	539.80 (225.78 to 883.44)	638.35 (254.44 to 1,053.07)	0.49 (0.29 to 0.69)
Middle SDI	212,357 (97,144 to 342,250)	1,109,676 (453,532 to 1,834,919)	175.65 (80.54 to 282.71)	332.67 (136.12 to 550.09)	1.92 (1.83 to 2.00)
Low-middle SDI	83,933 (38,268 to 132,586)	449,316 (184,706 to 736,548)	118.16 (54.10 to 186.67)	256.72 (105.99 to 421.00)	2.48 (2.38 to 2.58)
Low SDI	28,651 (12,374 to 45,954)	104,123 (40,372 to 172,587)	106.41 (45.94 to 170.57)	178.94 (69.28 to 296.48)	1.64 (1.57 to 1.71)
High-income Asia Pacific	69,905 (36,125 to 109,165)	192,036 (83,961 to 315,204)	275.59 (142.76 to 430.22)	311.70 (133.70 to 513.40)	−0.04 (−0.10 to 0.02)
High-income North America	431,674 (155,237 to 727,594)	869,221 (337,525 to 1,424,523)	928.60 (333.43 to 1,564.76)	979.31 (380.40 to 1,603.99)	0.15 (−0.11 to 0.41)
Western Europe	615,230 (227,702 to 1,037,435)	963,954 (351,861 to 1,644,874)	803.75 (296.40 to 1,356.86)	782.88 (286.66 to 1,334.69)	−0.21 (−0.29 to −0.13)
Australasia	24,550 (8,792 to 41,636)	60,266 (22,396 to 100,836)	791.64 (282.95 to 1,342.84)	845.90 (314.50 to 1,413.62)	0.13 (−0.07 to 0.34)
Andean Latin America	9,616 (4,730 to 15,701)	44,235 (19,956 to 73,883)	400.28 (197.45 to 653.26)	612.84 (276.56 to 1,023.61)	1.16 (0.61 to 1.71)
Tropical Latin America	42,086 (17,262 to 69,563)	187,841 (72,116 to 315,636)	386.96 (159.31 to 639.36)	581.34 (223.16 to 976.63)	1.28 (1.16 to 1.41)
Central Latin America	39,359 (17,627 to 63,830)	192,127 (80,021 to 319,170)	407.26 (182.82 to 660.48)	618.59 (257.62 to 1,028.23)	1.43 (1.36 to 1.50)
Southern Latin America	53,811 (21,923 to 89,870)	113,552 (45,406 to 187,322)	907.97 (369.93 to 1,516.73)	1,008.78 (403.52 to 1,663.08)	0.28 (0.18 to 0.39)
Caribbean	12,161 (4,757 to 20,014)	41,259 (16,692 to 68,651)	378.67 (147.97 to 623.28)	614.73 (248.71 to 1,022.81)	1.43 (1.18 to 1.67)
Central Europe	183,503 (78,565 to 297,962)	349,074 (143,209 to 577,826)	934.23 (399.82 to 1,517.31)	1,155.44 (473.22 to 1,913.36)	0.43 (0.29 to 0.57)
Eastern Europe	275,945 (115,280 to 449,004)	506,572 (202,156 to 823,496)	740.58 (309.34 to 1,203.25)	1,048.83 (418.77 to 1,704.99)	0.91 (0.42 to 1.40)
Central Asia	32,099 (13,311 to 52,657)	59,681 (24,190 to 99,361)	555.95 (231.03 to 911.05)	590.18 (239.74 to 983.02)	0.04 (−0.20 to 0.28)
North Africa and Middle East	68,388 (30,941 to 109,047)	335,061 (138,713 to 546,499)	347.62 (157.87 to 555.25)	639.06 (264.76 to 1,043.17)	1.60 (1.51 to 1.70)
South Asia	34,109 (16,721 to 56,157)	249,213 (105,712 to 410,715)	51.21 (25.39 to 84.01)	136.35 (58.28 to 225.01)	3.12 (2.96 to 3.27)
Southeast Asia	30,578 (14,715 to 48,609)	197,286 (81,571 to 327,001)	100.99 (49.09 to 160.37)	241.46 (100.65 to 400.01)	2.48 (2.41 to 2.55)
East Asia	139,181 (68,505 to 223,537)	845,921 (345,231 to 1,443,795)	133.02 (65.29 to 213.50)	302.04 (123.47 to 515.53)	2.34 (2.16 to 2.52)
Oceania	882 (356 to 1,507)	2,817 (1,085 to 4,685)	256.99 (103.66 to 439.31)	334.87 (128.73 to 557.01)	0.69 (0.61 to 0.77)
Western Sub-Saharan Africa	16,940 (5,195 to 29,065)	70,823 (19,589 to 123,815)	162.92 (49.8 to 279.98)	325.1 (90.64 to 567.88)	2.07 (1.97 to 2.16)
Eastern Sub-Saharan Africa	11,769 (5,114 to 19,145)	47,423 (17,235 to 80,563)	133.83 (57.96 to 217.58)	252.17 (91.24 to 429.16)	1.83 (1.75 to 1.92)
Central Sub-Saharan Africa	3,265 (1,151 to 5,622)	15,855 (5,242 to 28,863)	120.57 (42.11 to 209.23)	261.38 (85.56 to 478.08)	2.49 (2.43 to 2.56)
Southern Sub-Saharan Africa	11,252 (3,782 to 19,176)	54,504 (18,555 to 91,390)	356.59 (119.72 to 607.85)	798.92 (272.10 to 1,342.59)	2.38 (2.00 to 2.77)

DALYs, disability-adjusted life years; ASDR, age-standardized DALYs rate; CI, confidence interval; AAPC, average annual percentage change; SDI, socio-demographic index.

#### 3.1.2 At continental and SDI regional levels

**Figure 2:**
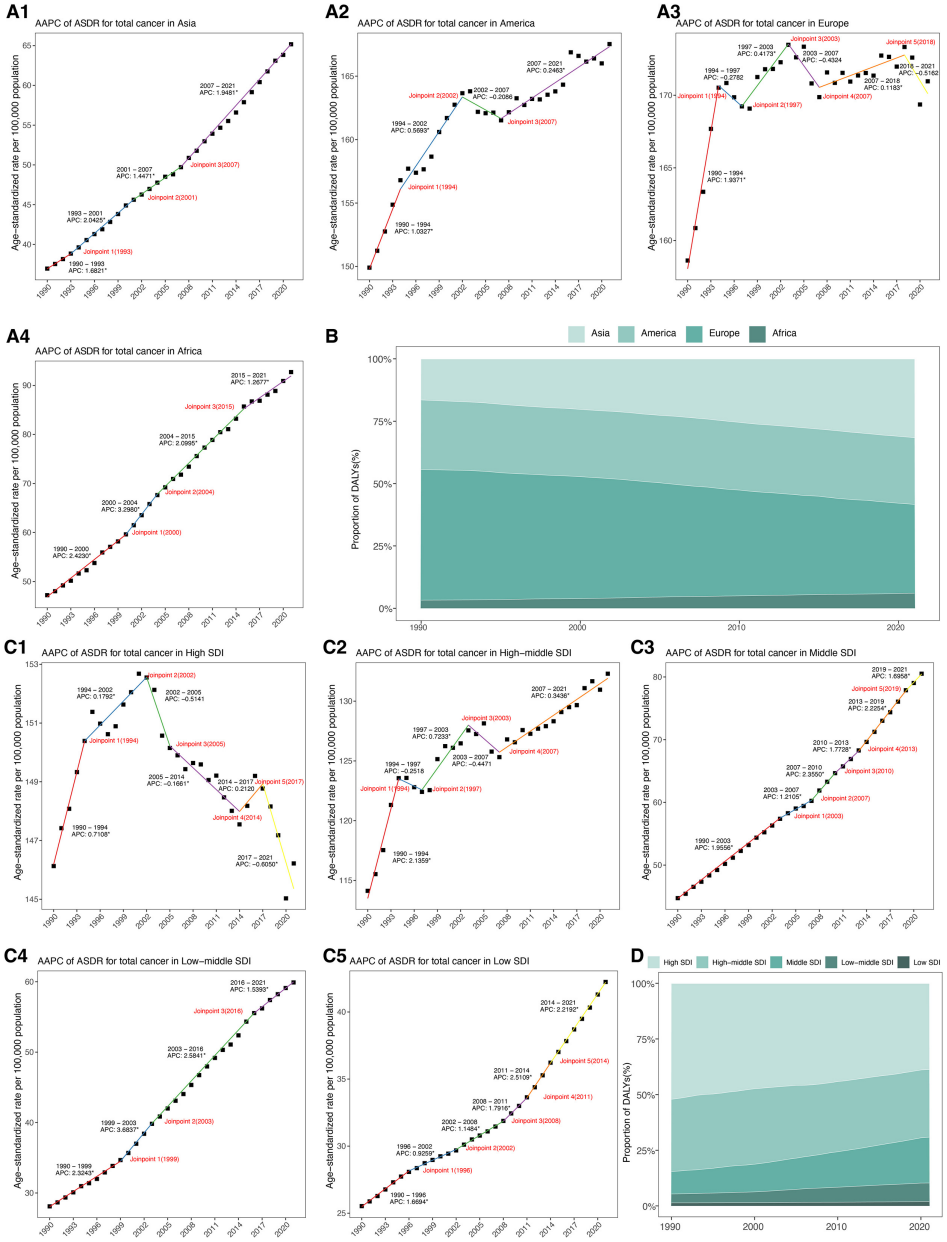
**(A)** Joinpoint regression analysis of ASDR for total cancer attributable to high BMI among older people from 1990 to 2021 across four continental regions. **(B)** Proportion of DALYs for total cancer attributable to high body mass index from 1990 to 2021 across four continental regions. **(C)**. Joinpoint regression analysis of ASDR for total cancer attributable to high BMI among older people from 1990 to 2021 across five SDI regions. **(D)** Proportion of DALYs for total cancer attributable to high body mass index from 1990 to 2021 across five SDI regions. DALYs, disability-adjusted life years; ASDR, age-standardized DALYs rate; AAPC, average annual percentage change; SDI, socio-demographic index.

Among the four continental regions, Europe and America bore the greatest ASDRs in 2021 (Table 1, Figure 2A). However, their AAPCs remained relatively modest (0.24% and 0.36%, respectively). By contrast, Asia, despite lower absolute ASDR in 2021, experienced a more rapid increase with an AAPC of 1.85% (95% CI: 1.77–1.93). Africa exhibited both the lowest absolute burden in 1990, yet showed the highest AAPC, rising at 2.20% (95% CI: 2.06–2.34) over the period. Additionally, Asia's DALYs proportion nearly doubled from 16.49% to 31.60% between 1990 and 2021, Europe's decreased from 52.37% to 35.61%, while America remained stable around 27%, and Africa's share rose modestly from 3.26% to 6.06% (Figure 2B, Supplementary Table S5).

Across five SDI regions, high-SDI region continued to report the highest ASDR in 2021, yet showed a slight, non-significant decline over time (AAPC: −0.02%, 95% CI: −0.17–0.14) (Table 1, Figure 2C). Conversely, low-middle SDI regions demonstrated the most rapid increase, with an AAPC of 2.48% (95% CI: 2.38 to 2.58), and their ASDR more than doubled from 118.16 to 256.72 per 100,000 population. Middle SDI and low SDI regions also showed sustained upward trends, with AAPCs of 1.92% and 1.64%, respectively. From 1990 to 2021, the proportion of DALYs attributable to high BMI decreased markedly in high SDI regions from 52.10% to 38.75%, and slightly declined in middle SDI regions from 32.44% to 30.40% (Figure 2D, Supplementary Table S6). In contrast, low-middle SDI and low SDI regions experienced substantial increases. High-middle SDI regions maintained a relatively stable proportion around 30% to 32%, positioning between the high and middle SDI groups.

#### 3.1.3 At GBD regional and national levels

**Figure 3:**
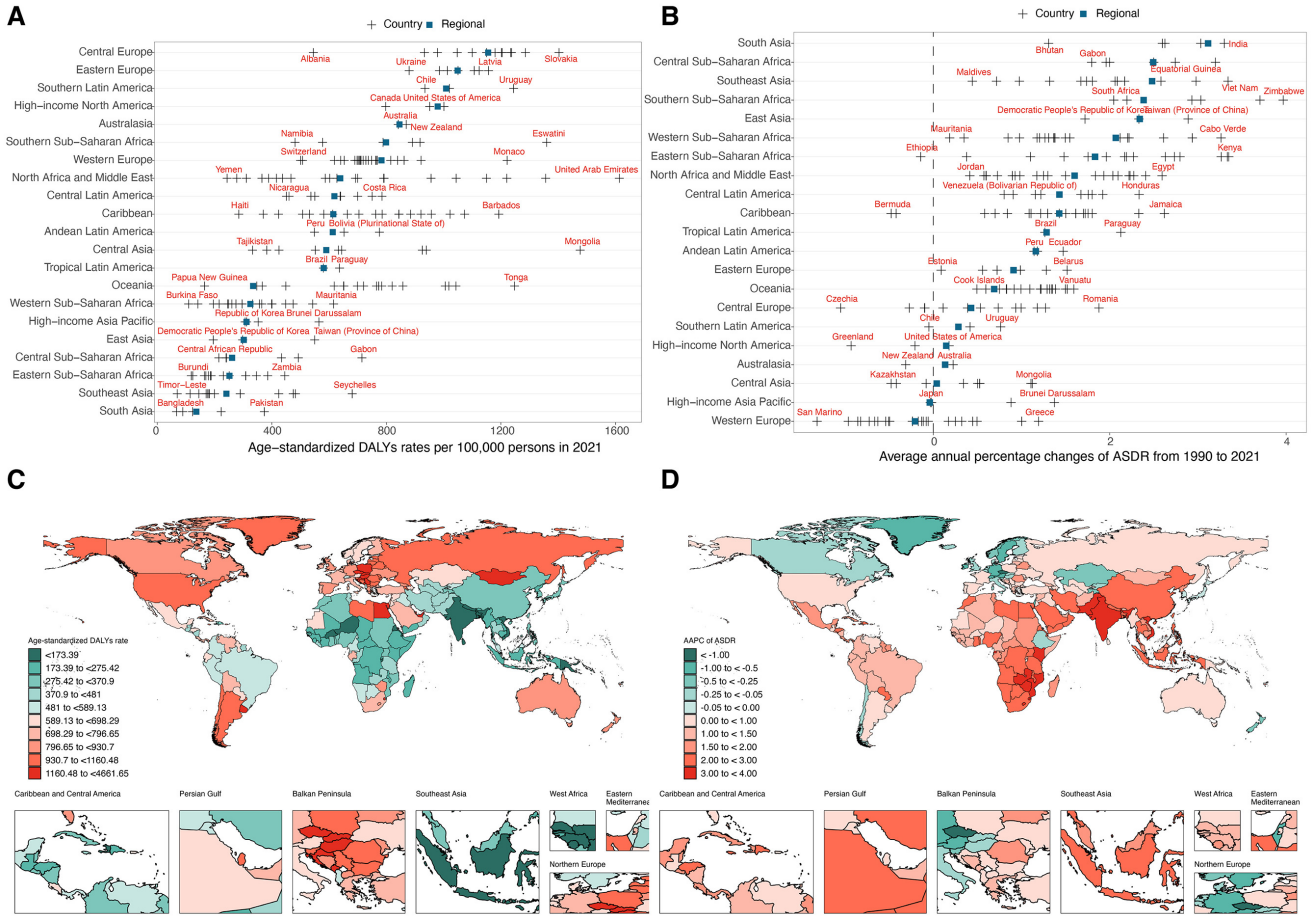
ASDR of total cancer attributable to high BMI among older people in 2021 across GBD regional and national levels, with AAPC from 1990 to 2021. **(A)** ASDR in 2021 across GBD regional and national levels. **(B)** AAPC of ASDR from 1990 to 2021 across GBD regional and national levels. **(C)** Map of ASDR in 2021 among 204 countries and territories. **(D)** Map of AAPC from 1990 to 2021 among 204 countries and territories. DALYs, disability-adjusted life years; ASDR, age-standardized DALYs rate; AAPC, average annual percentage change.

Substantial heterogeneity was observed across the 21 GBD regions. In 2021, the highest ASDRs were found in Central Europe, Eastern Europe, and Southern Latin America (Table 1, Figure 3A). However, growth in these regions remained modest (AAPC: 0.28% to 0.91%) (Table 1, Figure 3B). Notably, South Asia exhibited the highest AAPC, increasing at 3.12% (95% CI: 2.96–3.27), followed by Central Sub-Saharan Africa (AAPC: 2.49%; 95%CI: 2.43–2.56) and Southeast Asia (AAPC: 2.48%, 95%CI: 2.41–2.55) (Table 1, Figure 3B). In contrast, ASDR declined or remained stable in certain high-income regions, such as Western Europe (AAPC: −0.21%, 95% CI: −0.29 to −0.13) and high-income Asia-Pacific (AAPC: −0.04%, 95% CI: −0.10 to 0.02) (Table 1, Figure 3B). Among Latin American regions, Central Latin America, Tropical Latin America, and the Caribbean exhibited steady increases in ASDR (AAPCs ranging from 1.28% to 1.43%). Similarly, Andean Latin America showed an AAPC of 1.16% (95% CI: 0.61–1.71). Meanwhile, Oceania reported a lower AAPC of 0.69% (95% CI: 0.61–0.77) and moderate increase in ASDR (Table 1, Figure 3B).

At national levels, the highest ASRs for cancer-related DALYs attributable to high BMI were observed in United Arab Emirates in 2021, followed by Mongolia and Slovakia (Figure 3C, Supplementary Table S7). Conversely, the lowest ASDRs were recorded in Bangladesh, Timor-Leste, and Nepal. From 1990 to 2021, the AAPC in ASDRs revealed significant increases in several countries, with Zimbabwe exhibiting the highest increase (AAPC: 3.97%, 95% CI: 3.39–4.55), followed by Lesotho (AAPC: 3.70%, 95% CI: 3.49–3.92) and Kenya (AAPC: 3.35%, 95% CI: 3.17–3.53) (Figure 3D, Supplementary Table S7). In contrast, notable declines were observed in San Marino (AAPC: −1.32%, 95% CI: −1.60 to −1.32), Czechia (AAPC: −1.05%, 95% CI: −1.17 to −0.93), and Austria (AAPC: −0.96%, 95% CI: −1.19 to −0.74).

### 3.2 Burden of 11 specific cancer types attributable to high BMI among older people

**Figure 4:**
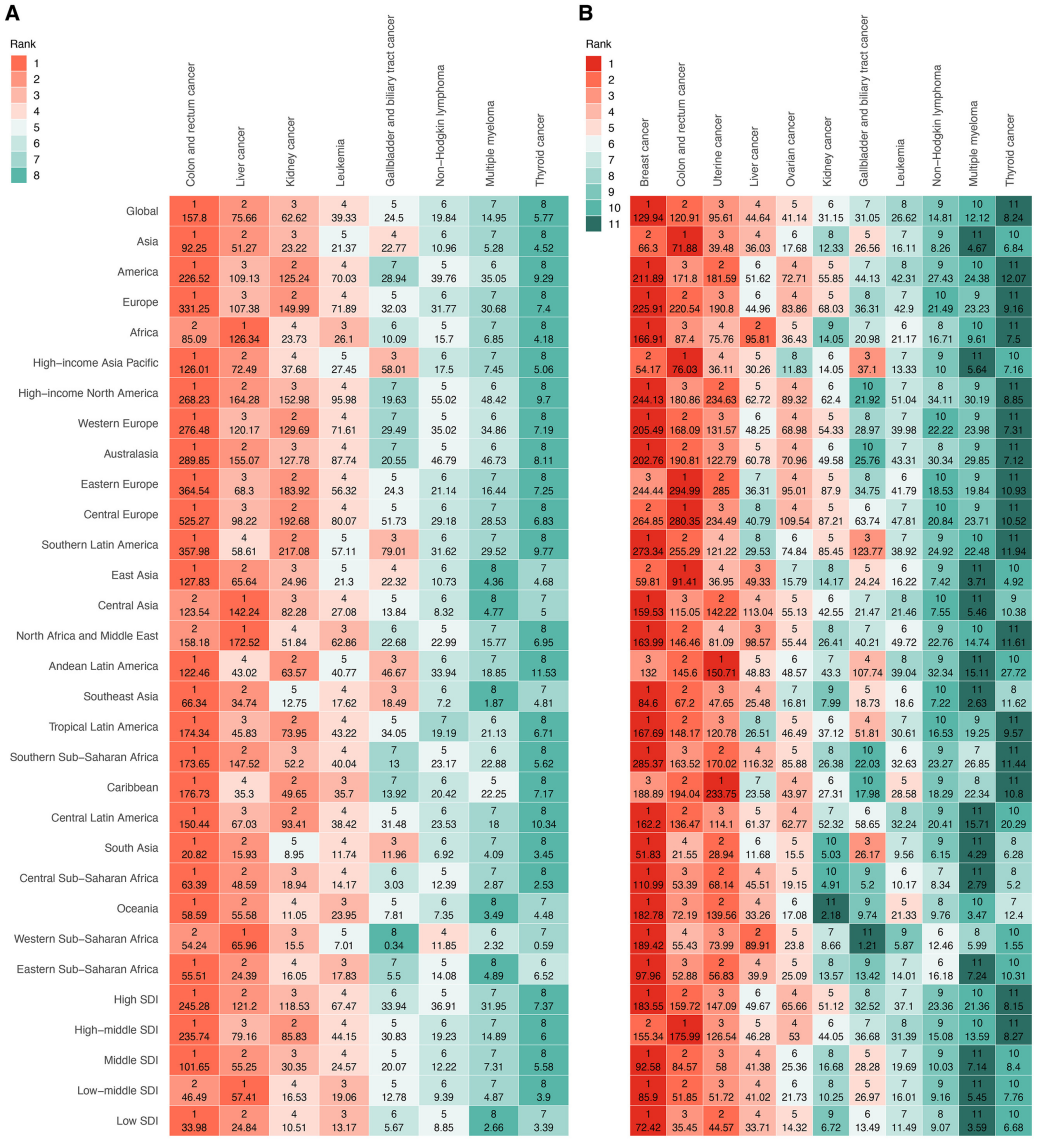
Ranking of ASDR for specific cancer types attributable to high BMI among older people globally and regionally in 2021, by sex. **(A)** Eight cancer types in male. **(B)** 11 cancer types in female. ASDR, age-standardized DALY rate; DALYs, disability-adjusted life years; SDI, socio-demographic index.

**Figure 5:**
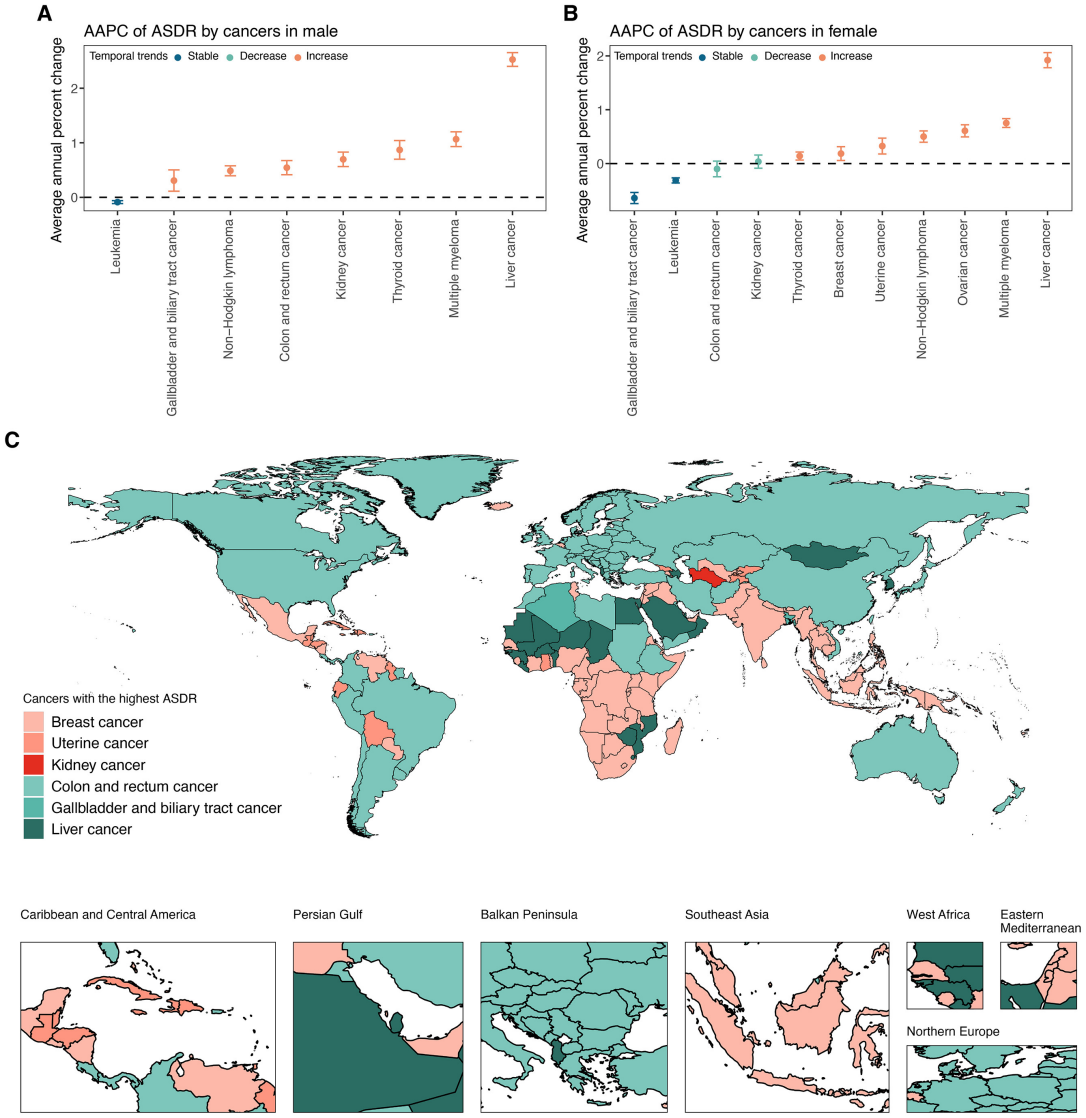
**(A)** AAPC of ASDR for specific cancer types attributable to high BMI among elderly men at global from 1990 to 2021. **(B)** AAPC of ASDR for specific cancer types attributable to high BMI among elderly women at global from 1990 to 2021. **(C)** Map of the cancers with the highest ASDR attributable to high BMI among the elderly across 204 countries and territories in 2021. ASDR, age-standardized DALY rate; DALYs, disability-adjusted life years; SDI, socio-demographic index.

In 2021, the global ASDR for 11 investigated cancer types varied substantially by site and sex (Figure 4, Supplementary Table S8). Among men, colon and rectum cancer ranked first, followed by liver cancer and kidney cancer (Figure 4A, Supplementary Table S8). Among women, breast cancer had the highest global ASDR, followed by colon and rectum cancer and uterine cancer (Figure 4B, Supplementary Table S8). Thyroid cancer exhibited the lowest global ASDR in both sexes. From 1990 to 2021, the global ASDR for most cancers showed an upward trend, although the extent varied by site and gender (Figures 5A, B; Supplementary Table S8). Among men, liver cancer recorded the most pronounced increase, followed by multiple myeloma, and thyroid cancer (Figure 5A, Supplementary Table S8). Colon and rectum cancer, non-Hodgkin lymphoma, and leukemia showed smaller changes, with leukemia exhibiting a slight decline. In women, the largest increases were seen in liver cancer, followed by multiple myeloma, and ovarian cancer (Figure 5B, Supplementary Table S8). Kidney cancer and colon and rectum cancer remained stable, while leukemia and gallbladder and biliary tract cancer decreased steadily.

Marked disparities in ASDRs were observed across the four continents, with Europe and the Americas consistently exhibiting the highest ASDRs for most solid tumors, particularly colorectal, breast, uterine, and kidney cancers, whereas Africa disproportionately bore the burden of liver cancer in both sexes (Figure 4, Supplementary Table S8). Hematologic malignancies, including leukemia, non-Hodgkin lymphoma, and multiple myeloma, showed a more balanced but still geographically variable distribution. Across five SDI levels, high and high-middle SDI regions consistently demonstrated the greatest ASDR for most sites, while low SDI regions had the lowest (Figure 4, Supplementary Table S8). For example, among men, the ASDR for colon and rectal cancer in high SDI regions was more than sevenfold higher than in low SDI regions. Among women, ovarian cancer-related ASDR in high SDI regions was nearly fivefold higher than low SDI regions. Within 21 GBD regions, the most extreme ASDRs were observed in high-income areas for many solid tumors, particularly colon, rectal, breast, uterine, and kidney cancers, as well as in selected middle-income regions for specific cancers, such as gallbladder cancer in Andean Latin America (Figure 4, Supplementary Table S8). In contrast, sub-Saharan Africa generally exhibited lower ASDRs, except for breast cancer in women. Overall, substantial sex disparities were noted in several cancers (Figure 4, Supplementary Table S8). Female-specific cancers (breast, ovarian, and uterine) dominated the ASDR profile in women. In contrast, liver, kidney, and colorectal cancers disproportionately affected men, particularly in Europe, the Americas, and high-income Asian regions.

Across 204 countries and territories, the leading cancer type in terms of ASDR attributable to high BMI among older adults varied substantially (Figure 5C, Supplementary Table S8). Breast cancer emerged as the predominant contributor, representing the highest ASDR in 87 countries and territories across all continents. Colon and rectum cancer ranked second, leading in 74 countries, particularly in Europe, the Americas, and parts of Asia-Pacific. Liver cancer was the leading type in 22 countries, with a concentration in East Asia, the Pacific Islands, and selected African settings. Uterine cancer was the most burdensome in 18 countries, largely in Latin America, the Caribbean, and parts of Africa. Gallbladder and biliary tract cancer and kidney cancer were less common as the leading types, observed in only two and one countries, respectively.

### 3.3 Across different age groups

**Figure 6:**
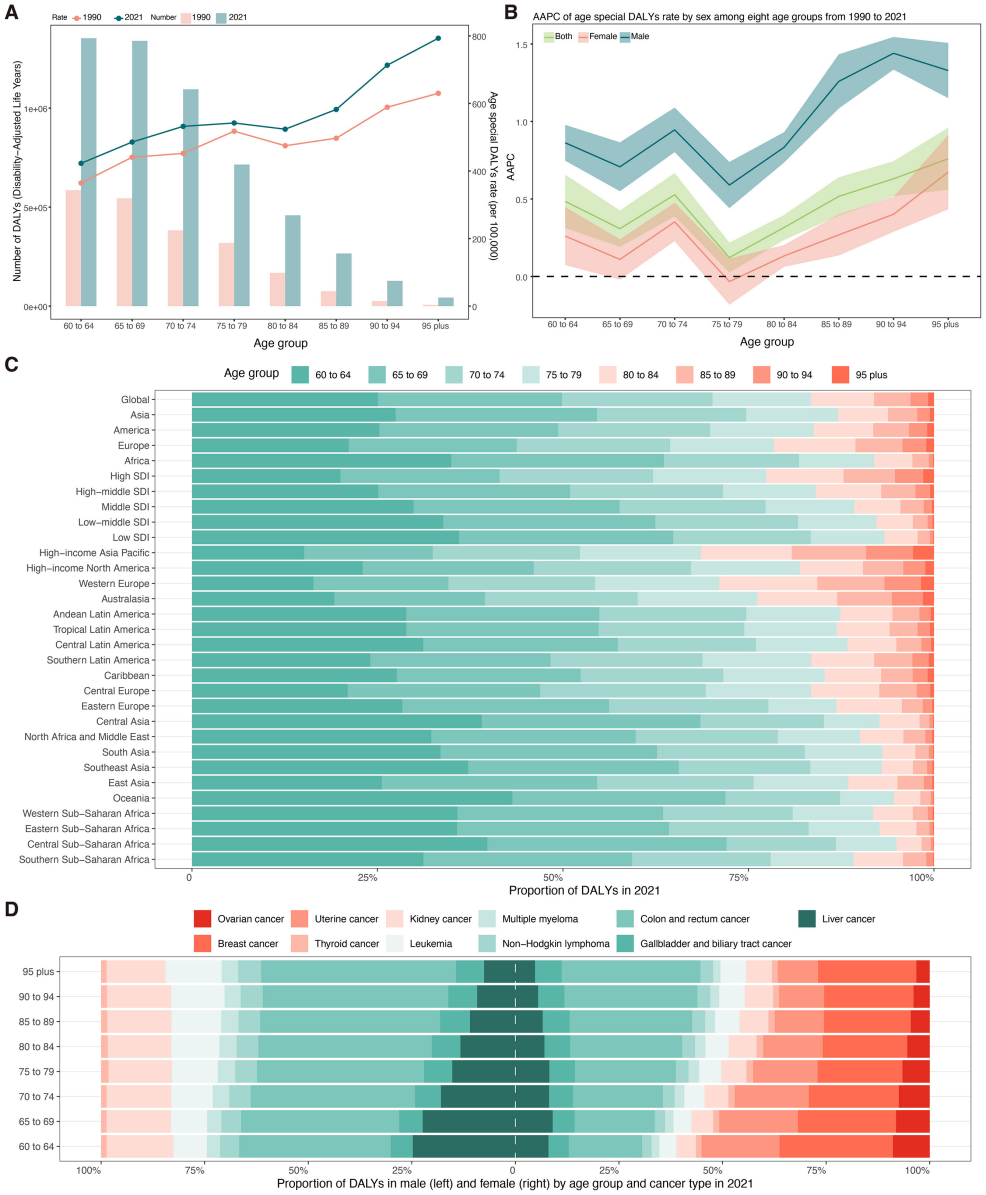
**(A)** Number and age-specific rates of DALYs for total cancer attributable to high BMI among older people by age groups in 2021. **(B)** AAPC of age-specific DALYs rates for total cancer attributable to high BMI among older people by age groups from 1990 to 2021. **(C)** Proportion of DALYs for total cancer attributable to high body mass index from 1990 to 2021 by age groups and regions. **(D)** Proportion of DALYs attributable to high body mass index from 1990 to 2021 by sex, cancer subtypes, and age groups. DALYs, disability-adjusted life years; AAPC, average annual percent change; SDI, socio-demographic index.

In 2021, the global DALYs of total cancer attributable to high BMI among older adults showed substantial increases across all age groups compared with 1990 (Figure 6A, Supplementary Table S9). For those aged 60 to 64 years, DALYs increased from 585,316 (95% UI: 236,528–964,786) to 1,353,072 (95% UI: 523,619–2,202,283), with the age-specific DALYs rate rising from 364.43 to 422.77 per 100,000 population, corresponding to an AAPC of 0.48% (95% CI: 0.31–0.66) (Figures 6A, B; Supplementary Table S9). The highest age-specific DALYs rates were observed in the oldest age groups, peaking at 793.04 per 100,000 (95% UI: 285.65–1360.39) in those aged 95 years and older, with an AAPC of 0.76% (95% CI: 0.56–0.96). Sex-stratified analysis revealed that males experienced greater relative increases in DALYs rates compared to females across all age groups (Figure 6B, Supplementary Table S9). For example, males aged 70 to 74 showed an increase from 327.76 to 437.70 per 100,000, with an AAPC of 0.95% (95% CI: 0.80–1.09), while females in the same group had a smaller increase (AAPC: 0.35%, 95% CI: 0.23 to 0.48). Males aged 85 to 89 and older cohorts exhibited the steepest rises, with AAPCs exceeding 1.20%. Conversely, females maintained higher absolute rates but lower annual increases.

Figure 6C presents the proportion of DALYs attributable to BMI in 2021 across age groups stratified by global, continental, SDI level, and regional levels. Globally, the burden was most concentrated in the 60–64 (25.06%) and 65–69 (24.82%) age groups (Supplementary Table S10). Africa, South Asia, and Southeast Asia exhibited a higher proportion of DALYs among those aged 60–64 years (34.96%−37.25%), while Western Europe, Australasia, and High-income Asia Pacific demonstrated a more balanced distribution, with elevated proportions in older groups (Figure 6C, Supplementary Table S10). Among males, colorectal cancer consistently accounted for the largest proportion of DALYs across all age groups, rising from 36.60% (60–64 years) to 47.04% (≥95 years) (Figure 6D, Supplementary Table S11). This was followed by liver and kidney cancer. Breast cancer was the leading contributor among women aged 60–64 years (27.37%), but its relative contribution declined with age, while the proportion of DALYs due to colorectal cancer increased steadily from 17.72% (60–64 years) to 33.49% (≥95 years) (Figure 6D, Supplementary Table S10).

### 3.4 Decomposition of the changes in DALYs for total cancer

**Figure 7:**
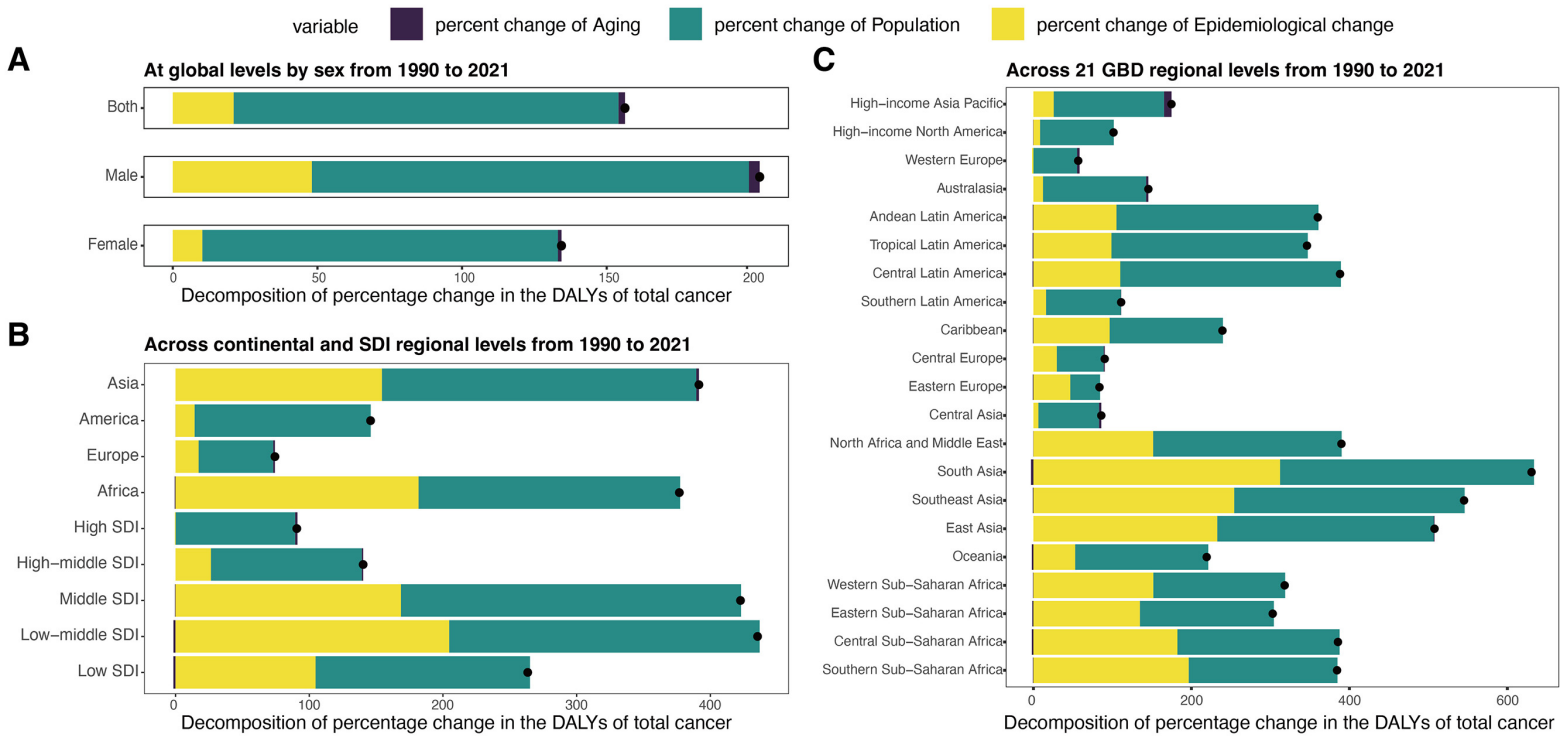
Decomposition analysis of DALYs for total cancer attributable to high BMI among older people by region from 1990 to 2021. **(A)** Decomposition analysis of DALYs at global levels by sex from 1990 to 2021. **(B)** Decomposition analysis of DALYs across continental and SDl regional levels from 1990 to 2021. **(C)** Decomposition analysis of DALYs across 21 GBD regional levels from 1990 to 2021. DALYs, disability-adjusted life years; ASDR, age-standardized DALYs rate; SDI, socio-demographic index.

From 1990 to 2021, the global DALYs for total cancer attributable to high BMI among older adults increased by 3.29 million, corresponding to a 156.31% rise. This increase was largely driven by population growth (133.04%) and, to a lesser extent, epidemiological change (21.04%), with population aging contributing modestly (2.23%) (Figure 7A, Supplementary Table S12). Males experienced a greater relative increase (202.87%), largely attributed to demographic expansion (151.11%) and an epidemiological shift (48.05%), with population aging accounting for 3.71%. Among females, the rise was slightly lower (134.36%) mainly driven by demographic expansion (122.93%), with only 10.20% attributable to epidemiologic changes and 1.24% to aging.

Among four continental regions, the greatest relative increases were observed in Asia (391.46%) and Africa (376.82%), mainly due to population growth and changes in epidemiology (Figure 7B, Supplementary Table S12). Europe experienced a modest increase (74.32%), with epidemiological change (17.19%) contributing more than aging (1.37%). By SDI level, low-middle and middle SDI regions experienced the highest proportional increases (435.33% and 422.55%, respectively). In contrast, high SDI regions showed a smaller increase (90.68%), mainly explained by population growth (89.44%), with minimal contributions from aging (1.72%) and a slight decline from epidemiological change (−0.48%). Across the 21 GBD regions, the most pronounced DALYs increases occurred in South Asia, Southeast Asia, and East Asia, each exceeding 500%, primarily driven by population growth and epidemiologic shifts (Figure 7C, Supplementary Table S12). In contrast, regions like Western Europe, Central Asia, and Central Europe experienced more moderate growth (< 100%), primarily driven by population growth, with limited or negative input from aging and epidemiologic shifts.

### 3.5 Cross-country inequalities analysis of ASDR for total cancer

**Figure 8:**
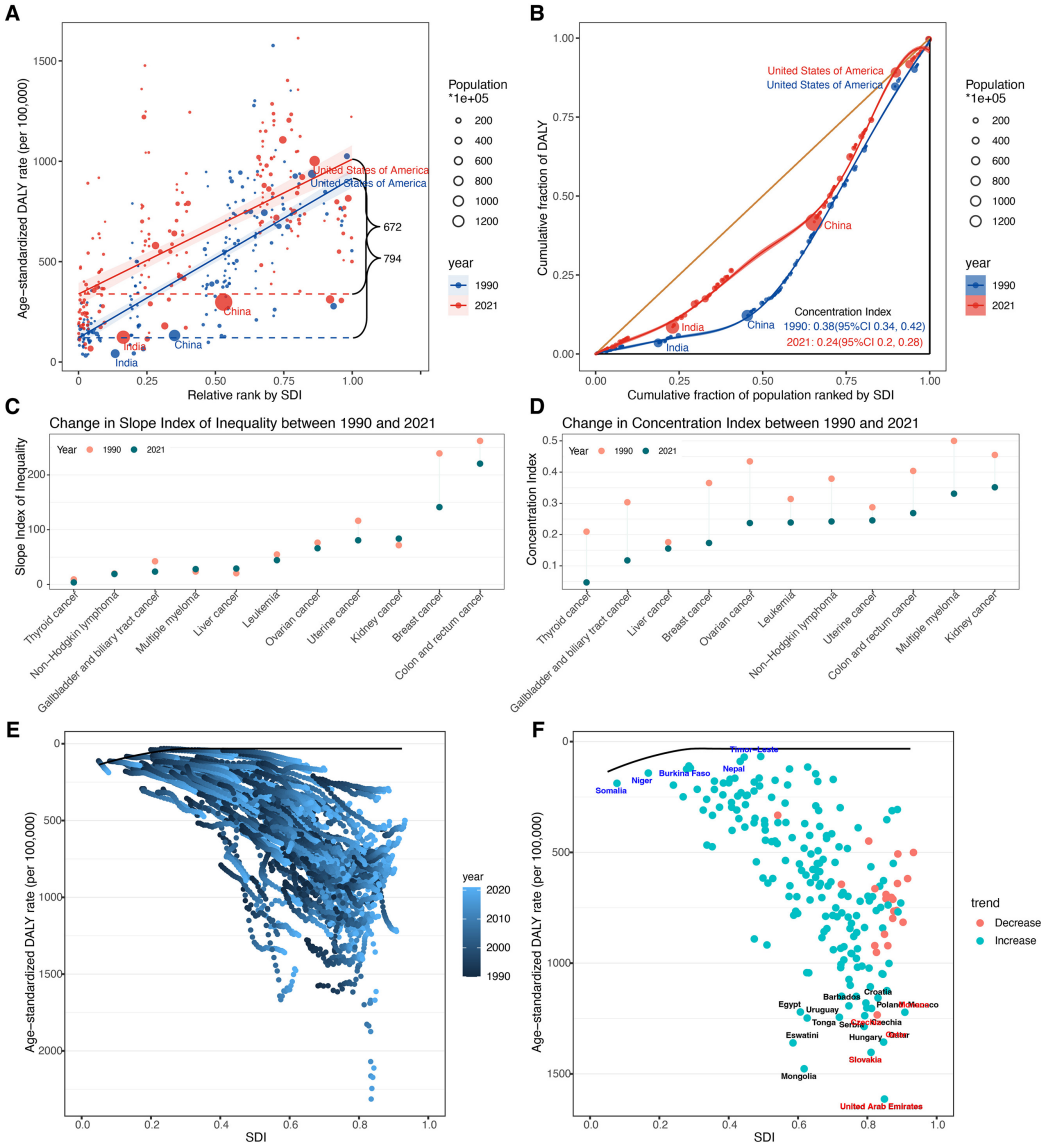
**(A)** Health inequality regression curves in 1990 and 2021. **(B)** Concentration curves in 1990 and 2021. **(C)** Absolute cross-country inequalities across 11 cancer types in 1990 and 2021. **(D)** Relative cross-country inequalities across 11 cancer types in 1990 and 2021. **(E)** Frontier analysis based on SDI and total cancer related ASDR in 204 countries and territories from 1990 to 2021. **(F)** Frontier analysis based on SDI and total cancer related ASDR in 204 countries and territories in 2021. The top 15 countries or regions with the largest effective difference were labeled in black (United Arab Emirates, Mongolia, Slovakia, Eswatini, Qatar, Hungary, Tonga, Uruguay, Serbia, Czechia, Monaco, Egypt, Poland, Croatia, and Barbados). Countries or regions with low SDI (<0.4658) and a low effective difference were labeled in blue (Timor-Leste, Nepal, Niger, Somalia, and Burkina Faso), conversely, those countries or regions with high SDI (>0.8103) and relatively high effective difference were labeled in red (United Arab Emirates, Slovakia, Qatar, Czechia, and Monaco). ASDR, age-standardized DALY rate; DALYs, disability-adjusted life years; SDI, socio-demographic index; SII, slope index of inequality; CIX, concentration index.

Globally, the DALY burden of total cancer attributable to high BMI among older adults was predominantly concentrated in higher SDI countries in both 1990 and 2021 (Figures 8A, B). During the study period, the SII for ASDR declined from 794.26 (95% CI: 714.86–873.65) to 671.51 (95% CI: 565.04–777.98) per 100,000 population, reflecting a reduction in absolute inequality. Similarly, the CIX decreased from 0.3772 (95% CI: 0.3373–0.4171) to 0.2393 (95% CI: 0.1972–0.2813), indicating a narrowing of relative inequality while the burden remained disproportionately higher in high-SDI settings.

Significant absolute and relative SDI-related inequalities in ASDR were also observed across 11 cancer types (Figures 8C, D; Supplementary Table S13). Breast and colorectal cancers showed the largest absolute inequalities in both 1990 and 2021. From 1990 to 2021, the SII of breast cancer declined from 239.24 (95% CI: 209.91–268.58) to 141.23 (95% CI: 102.14–180.33) and that of colorectal cancer from 262.09 (95% CI: 236.91–287.26) to 220.55 (95% CI: 193.09–248.02), indicating a narrowing gap between the highest and lowest SDI countries. The CIX for both cancers also decreased, from 0.3652 (95% CI: 0.3182–0.4122) to 0.1732 (95% CI: 0.1259–0.2206) and from 0.4038 (95% CI: 0.3615–0.4461) to 0.2688 (95% CI: 0.2287–0.3089), respectively. Notably, kidney cancer showed a slight increase in absolute inequality [SII: 71.73 (95% CI: 63.39–80.08) to 83.75 (95% CI: 73.25 to 94.25)], despite a decrease in relative inequality, and exhibited the largest relative inequality in 2021 (CIX: 0.3515, 95% CI: 0.2943–0.4088). Multiple myeloma also ranked high in relative inequality in 2021 (CIX: 0.3310, 95% CI: 0.2707–0.3913). Conversely, thyroid cancer showed the smallest inequalities in 2021, with both the lowest absolute (SII: 3.82, 95% CI: 1.98–5.66) and relative (CIX: 0.0469, 95% CI: 0.0113–0.0826) measures.

### 3.6 Frontier analysis of ASDR for total cancer

In the frontier analysis, we compared each country's ASDR of total cancers attributable to high BMI among older adults with the optimal rate predicted for its corresponding SDI in 2021 (Figures 8E, F; Supplementary Table S14). The largest effective differences occurred in several high- and high–middle SDI countries, including the United Arab Emirates (1,581.21), Mongolia (1,444.51), Slovakia (1,370.57), Eswatini (1,327.68), and Qatar (1,324.10). The smallest effective differences were found in Bangladesh (34.85), Timor-Leste (38.04), Nepal (56.88), Niger (57.59), and Viet Nam (82.80).

## 4 Discussion

Our study provides the first comprehensive global analysis of high BMI-attributable cancer burden specifically among older adults, revealing several critical patterns and disparities. We observed a substantial overall rise in cancer burden linked to high BMI in adults aged 60 and above since 1990, with total DALYs more than doubling and the global ASDR increasing modestly. Notably, the relative increase was steeper in males than in females, even though older women still experienced higher absolute ASDRs. Consistent with these sex differences, we found colorectal cancer contributed the highest ASDR in men, while breast cancer contributed the most in women by 2021, aligning with known obesity-related cancer patterns in each sex. Geographically, our results highlighted stark disparities. The steepest ASDR increases occurred in Asia and Africa, whereas Europe and the Americas currently still bear the highest ASDR despite more modest growth. Importantly, we documented persisting global inequalities. The burden remains disproportionately concentrated in higher SDI countries in 2021, but the gap has narrowed over time as low- and middle-SDI regions' share of DALYs grew.

The rising trend of high BMI-related cancer burden in older adults could be interpreted in light of both demographic shifts and epidemiological changes. Our findings align with other recent analyses that have documented rising cancer burdens attributable to obesity. For instance, Tan et al. reported a 34% increase in cancer DALYs due to high BMI from 2010 to 2019 globally, reflecting a worsening trend worldwide (17). Additionally, Zheng et al. revealed that the economic burden associated with high BMI—related cancers, as measured by total DALYs, has surged by 193% globally (24). The primary driver we identified through decomposition analysis was population expansion, which accounted for the majority of the DALY rise. In brief, more elderly individuals are now at risk of developing cancer due to high BMI. The rising prevalence of overweight is another critical driver. Data from the WHO recently demonstrated that the prevalence of overweight among adults has surged from 25% in 1990 to 43% in 2022 (6). Obesity is associated with chronic inflammation, hormonal imbalances (notably increased estrogen), and metabolic dysfunction (such as insulin resistance, hyperglycemia, and dyslipidemia). These biological effects collectively facilitate the development of cancer in multiple organs (36, 37). Today's older adults, having endured decades of the rising obesity epidemic, enter their 60s and beyond with a greater cumulative exposure to high BMI than previous generations. As a result, older adults now have a greater intrinsic risk of developing cancers linked to high BMI than those in earlier cohorts. Our finding of increased age-specific DALY rates, especially pronounced in males, supports this notion. Additionally, the past three decades of advancements in cancer detection and treatment provide context for this trend. Improvements in imaging, endoscopy, and screening programs may have increased cancer diagnoses in aging and obese populations (38, 39), thereby initially raising incidence rates and DALYs. Concurrently, modern therapies, including targeted treatments, immunotherapy, and advanced radiotherapy, have extended survival for many cancer patients (39). Longer survival means patients live more years with cancer (contributing to YLDs), which can paradoxically increase DALYs even as mortality is delayed. Our finding that men exhibited steeper rises in ASDRs than women may be partly explained by historically lower male obesity rates that are now converging with female rates. Additionally, men tend to accumulate more visceral adipose tissue, which is metabolically active and linked to cancers of the colon, liver, and kidney (40–42). These drivers underscore that the observed global trends may not solely a function of demographic change but also reflect lifestyle transitions, technological progress and evolving risk exposures.

Our detailed cancer subtype analysis revealed that not all high BMI-related cancers were rising equally, underscoring the need to consider cancer-specific and sex-specific patterns. We found that breast cancer and colorectal cancer stood out as the leading contributors to high BMI-attributable DALYs in women and men respectively, consistent with established epidemiology. Obesity is a well-known risk factor for postmenopausal breast cancer, likely due to higher estrogen levels from adipose tissue after menopause and other metabolic changes (36, 43), which explains why breast cancer remains the top contributor among older women. Similarly, excess body weight significantly raises the risk of colorectal cancer, as well as cancers of the uterus and kidney (41, 44); this is reflected in the high DALY contributions of these sites in our 2021 data. We further identified a pattern that is worth highlighting. Liver cancer exhibited one of the most substantial relative increases in DALY rates from 1990 to 2021 in both sexes. This sharp rise likely reflects the compounding impact of the global obesity epidemic, particularly through the obesity–non-alcoholic fatty liver disease (NAFLD)–hepatocellular carcinoma (HCC) pathway. NAFLD, now the most common chronic liver disease worldwide, could progress to non-alcoholic steatohepatitis (NASH), cirrhosis, and ultimately HCC, even in the absence of viral hepatitis (45–47). In regions such as East Asia and sub-Saharan Africa, where chronic HBV infection remains endemic, the rising prevalence of obesity and diabetes has further intensified the liver cancer burden among older adults. Previous cohort studies have demonstrated that obesity significantly increases the risk of HCC, especially in the presence of metabolic syndrome and chronic hepatitis (48–50). Another important observation was the marked increase in the BMI-attributable burden of multiple myeloma, which, alongside liver cancer, was among the fastest-growing cancer subtypes in older adults. Accumulating evidence suggests that obesity influences hematologic malignancies not only through systemic inflammation but also via the bone marrow microenvironment. Adipocytes within the bone marrow can modulate plasma cell proliferation and survival by secreting adipokines, pro-inflammatory cytokines, and free fatty acids, thereby creating a tumor-permissive niche (51, 52). Moreover, recent work has highlighted that obesity may accelerate the progression from monoclonal gammopathy of undetermined significance (MGUS) to myeloma by disrupting immune surveillance and promoting metabolic stress within the marrow niche (51–54). On the other hand, a few cancers showed stable or declining trends in high BMI-attributable ASDR. For instance, leukemia in males and gallbladder and biliary tract cancer in females exhibited modest decreases from 1990 to 2021. These patterns may reflect multifactorial influences. Improved treatment protocols and earlier detection may have contributed to reduced leukemia-related mortality and DALYs, potentially offsetting obesity-related risks. In the case of gallbladder and biliary tract cancer, better management of gallstone disease and reductions in other etiologic exposures, such as chronic infections (e.g., Salmonella typhi, liver flukes, or hepatitis viruses), in certain regions may have played a protective role. In addition, these trends may partly result from diagnostic uncertainty or data limitations. Gallbladder cancer, for example, is often underdiagnosed or misclassified due to overlapping symptoms with other hepatobiliary diseases and low diagnostic capacity in many low-income regions. Therefore, while the observed declines could reflect real epidemiologic shifts, they should be interpreted cautiously given the potential for underestimation and incomplete registry coverage. Our cross-country inequality analysis by cancer type further highlighted that certain cancers were contributing most to global disparities. Breast and colorectal cancers had the largest absolute inequalities in ASDR between high- and low-SDI countries, although these inequalities narrowed from 1990 to 2021 as developing countries caught up. This may indicate that the high incidence of breast and colon cancer, historically prevalent in high-income regions due to lifestyle factors such as diet, sedentary behavior, and better screening, is now becoming more common in transitioning countries as lifestyles change. Meanwhile, cancers such as kidney cancer and multiple myeloma exhibited some of the highest relative inequalities (concentrated in higher-SDI regions) even in 2021. This suggests that these high BMI-related cancers remain more prevalent in wealthier countries, possibly due to dietary patterns, such as high animal protein intake, or disparities in diagnostic capacity linked to economic development. In contrast, thyroid cancer attributable to high BMI showed minimal inequality and low burden, indicating a more uniform contribution globally. These patterns suggest that prevention strategies should be tailored by sex and cancer type. Weight management and colorectal cancer screening are priorities for both sexes, while attention to obesity's role in breast and uterine cancers is crucial for older women. The sharp rise in liver and myeloma burdens suggests a need for research into targeted interventions, such as aggressive management of metabolic syndrome and NAFLD in midlife, to curb the future burden of these cancers.

We found striking geographic and socioeconomic disparities in high BMI-related cancer burden among older adults, which have important implications for global health equity. High-income regions, particularly Europe and parts of the Americas, had the highest ASDRs in 2021, reflecting long-standing obesity prevalence and older population structures. In contrast, the fastest growth in ASDR occurred in Asia and Africa, where obesity prevalence and life expectancy have increased rapidly in recent decades. Many countries in these continents historically had lower obesity and cancer rates, but are now experiencing an upsurge as diets change, physical activity declines, and populations live longer. For instance, South Asia and parts of Sub-Saharan Africa showed some of the steepest ASDR increases, despite the low initial baseline. This means that regions which once faced primarily infectious diseases and undernutrition are now confronting the dual challenge of rising obesity-related cancers in their senior populations. Meanwhile, some high-income regions, such as high SDI countries in the Asia-Pacific or Western Europe, may be reaching a saturation point in obesity-related cancer incidence, potentially due to early interventions like public health campaigns and better primary prevention. Our analysis of the five SDI regions underscores that in 2021, the high SDI region had the highest ASDR, while the low SDI region exhibited the fastest relative growth, with its ASDR more than doubling since 1990. This indicates that socioeconomic development in countries is frequently accompanied by modern lifestyle changes, including sedentary behavior and high-calorie diets. These behaviors, coupled with an aging population, significantly increase the cancer burden among older adults with high BMI (55–58). Indeed, overweight and obesity are rising quickly in many low- and middle-income countries, effectively globalizing what was once thought to be a rich-country problem (59). Our analysis of health inequities indicates that global disparities in this burden have narrowed, with absolute inequities shrinking and the concentration index declining from 0.38 to 0.24. However, this convergence was largely driven by worsening trends in lower SDI countries rather than dramatic improvements in higher SDI countries. In other words, the narrowing of disparities was due to the rising ASDR of high BMI-related cancers in many lower SDI countries, rather than to the resolution of the issue in higher SDI countries. Therefore, despite the reduced disparity, significant inequities remained, with the ASDR in high-SDI countries still being much higher than in low-SDI countries. This cross-countries healthy inequities underscore that no country is immune to this issue. Achieving equitable healthy aging requires both sustained improvements in higher SDI countries and proactive obesity and cancer control strategies to help lower SDI countries “bend the curve” and avoid replicating the high cancer burden observed in wealthier countries (60, 61). Global initiatives should recognize that obesity and its cancer consequences are now a worldwide problem, and resources must be allocated accordingly to prevent widening disparities in the coming decades.

The frontier analysis compared each country's ASDR with the optimal ASDR predicted for its SDI level, revealing substantial variability beyond socio-economic development. Several higher SDI countries (e.g., United Arab Emirates, Slovakia, Qatar) had burdens far above expectations. However, it is important to note that countries above the frontier does not imply these countries are “causing” the global burden. Instead, such deviations highlight regions where outcomes could be improved. These disparities may reflect multiple underlying factors, including exceptionally high obesity prevalence, dietary patterns, insufficient screening, or health system limitations. For instance, in Gulf states, cultural and lifestyle factors, such as high-caloric diets and low physical activity in affluent settings, have led to some of the world's highest obesity rates, which likely contributes to their elevated cancer burden (62, 63). Conversely, Bangladesh, Timor-Leste, Nepal, Niger, and Viet Nam were much closer to the frontier, exhibiting lower-than-expected burdens. This could be attributable to traditionally lower obesity rates or unmeasured protective lifestyle factors, such as dietary components and increased physical activity in daily life. It might also reflect younger age structure within the older-adult category or underdiagnosis where health systems were less developed. Nevertheless, studying these lower-burden countries could provide lessons on protective factors or successful interventions. For instance, Bangladesh has a high prevalence of underweight in older ages (64), which might partially protect against some obesity-related cancers, though malnutrition carries its own risks. Countries that exceed the frontier should be prioritized for in-depth investigation and targeted interventions, such as integrating weight management into elder care, enhancing cancer screening, and improving physical activity infrastructure. Meanwhile, low-burden countries offer opportunities to study cultural or policy factors that may help reduce the impact of overweight and obesity. Crucially, our frontier analysis demonstrates that economic development alone does not guarantee low ASDR of cancer attributable to high BMI among older adults. Without effective public health measures, even resource-rich nations can underperform. Setting realistic benchmark goals and promoting cross-country learning could accelerate progress toward equitable cancer prevention.

Because high BMI is modifiable, the substantial burden we identified represents a major prevention opportunity. Diet plays a foundational role, as excessive intake of energy-dense, ultra-processed foods rich in saturated fats and added sugars contributes significantly to obesity. Replacing such diets with nutrient-dense, high-fiber, plant-based alternatives has shown promise in reducing cancer risk. Physical activity, even of low to moderate intensity, plays a pivotal role in maintaining energy balance, preserving muscle mass, and modulating inflammatory and hormonal pathways linked to cancer development. Weight management should go beyond short-term goals. It requires sustained, structured interventions including personalized counseling, regular weight monitoring, and community-driven support systems. These strategies should be embedded in routine care for older adults, integrated with national cancer screening programs. Furthermore, screening programs for breast and colorectal cancers could incorporate BMI as a stratification factor and link overweight participants with weight-loss resources. High SDI countries with persistent high ASDRs and those identified as frontier outliers should implement aggressive obesity-prevention campaigns among seniors. Low- and middle-SDI countries experiencing rapid increases need early interventions tailored to their contexts, drawing on successful strategies from high-income countries but adapted to local diets, resources, and cultural norms. International collaboration would be essential to avert a surge of high BMI-related cancers in emerging economies. Beyond cancer, managing high BMI in older adults would also reduce the burden of cardiovascular disease, diabetes, and other chronic conditions, aligning with the broader goal of healthy aging.

Several limitations should be acknowledged. First, GBD estimates are based on modeled data, not direct observation, and data quality varies widely, especially in low-income countries. Incomplete cancer registries and BMI misclassification may bias results. We interpreted findings from data-sparse regions with caution and emphasized patterns that were consistent across multiple geographic areas. Second, due to the ecological nature of the GBD data, we were unable to establish causality or adjust for individual-level confounders, such as diet, physical activity, alcohol consumption, and smoking. Nonetheless, the comparative risk assessment framework of GBD integrates extensive meta-analytic evidence and global exposure distributions to strengthen the interpretability of risk-outcome associations at the population level. Third, we did not distinguish between subpopulations within countries (e.g., urban vs. rural, or different socioeconomic strata), which may mask important within-country disparities. Future research using disaggregated subnational data could help uncover these inequalities. Fourth, although BMI is a widely used and practical proxy for adiposity in large-scale epidemiological research, it does not differentiate between lean mass and fat mass, nor does it capture visceral fat distribution, which may be more relevant to cancer risk. Incorporating alternative obesity metrics, such as waist circumference or waist-to-hip ratio, might improve future risk estimations. Fifth, although we focused on 11 high-BMI-related cancer types with robust evidence from the GBD 2021 risk factor framework, obesity may contribute to other malignancies not yet definitively established. As evidence evolves, future iterations of GBD may expand the scope of attributable cancer types. Despite these limitations, our study provides a comprehensive global assessment of high-BMI-related cancer burden among older adults using standardized methods, with robust stratification by location, sex, and cancer type. The integration of decomposition and inequality analyses further enhances the policy relevance of our findings by identifying key drivers of burden and highlighting cross-national disparities.

## 5 Conclusion

In conclusion, our study demonstrates that the global burden of cancer attributable to high BMI among older adults has risen dramatically since 1990, with considerable variation by sex, cancer site, geography, and socio-economic development. These insights underscore the necessity of weight management in later life, the importance of tailored prevention and screening by cancer type and sex, and the urgency of addressing emerging hotspots in low- and middle-SDI countries. The narrowing of global inequalities reflects a concerning convergence toward higher burdens rather than meaningful reductions in high-income countries. As the world strives to reduce the burden of non-communicable diseases and promote healthy aging, investing in high BMI control throughout the life course can significantly decrease future cancer-related ASDR, narrow observed inequalities between countries, and advance the global agenda of equitable and healthy aging.

## Data Availability

The original contributions presented in the study are included in the article/Supplementary material, further inquiries can be directed to the corresponding author.
