# Supplementary material for: Global burden, trends, and inequalities in cancer and subtypes attributable to high BMI among older adults, 1990–2021: a secondary analysis of the global burden of disease study 2021
Source: Front Nutr. 2025 Nov 11;12:1683893. doi: 10.3389/fnut.2025.1683893 (PMC12645832; doi:10.3389/fnut.2025.1683893)
Supplement: Supplementary file 1 [file Data_Sheet_1.pdf]

Global burden, trends, and inequalities in cancer and subtypes attributable to high BMI among older adults, 1990-2021: a secondary analysis of the global burden of disease study 2021

Yitong Huang[1], Di Qiu[2], Feng Xuan[3] *

[1] Department of Internal Medicine, Zhuji Maternal and Child Health Hospital, Shaoxing, Zhejiang, China

[2] Department of Hematology, Zhuji Affiliated Hospital of Wenzhou Medical University, Shaoxing, China

[3] Department of Radiation Oncology, Zhuji Affiliated Hospital of Wenzhou Medical University, Shaoxing, Zhejiang Province, China.

*** Correspondence:** Feng Xuan

Email: xfeng8901@outlook.com

Table S1: The Socio-demographic Index (SDI) reference values from the Global Burden of Disease data in 2021. 4

Table S2. The Socio-demographic Index (SDI) values of 204 countries/territories in 2021. 5

Table S3. International Classification of Diseases (ICD) diagnostic criteria for 11 types of cancers in the Global Burden of Disease (GBD) data. 14

Table S4: The correspondence of Global Burden of Disease (GBD) regions with 204 countries/territories in 2021. 15

Table S5. Proportion of DALYs for total cancer attributable to high body mass index from 1990 to 2021 across four continental levels. 17

Table S6. Proportion of DALYs for total cancer attributable to high body mass index from 1990 to 2021 across five SDI regional levels. 20

Table S7. Cancer-related disability-adjusted life years and age-standardized DALYs rates attributable to high BMI among older people among 204 countries and territories in 1990 and 2021, with average annual percent change from 1990 to 2021. 23

Table S8. ASDR for specific cancer types attributable to high BMI among older people globally and regionally in 2021, by sex. 37

Table S9. Number and age-specific rates of DALYs for total cancer attributable to high BMI among older people by age groups in 2021, with average annual percent change from 1990 to 2021. 80

Table S10. Proportion of DALYs for total cancer attributable to high body mass index from 1990 to 2021 by age groups and regions. 82

Table S11. Proportion of DALYs attributable to high body mass index from 1990 to 2021 by sex, cancer subtypes, and age groups. 84

Table S12. Decomposition analysis of DALYs for total cancer attributable to high BMI among older people by region, 1990 to 2021. 85

Table S13. Slope index of inequality and concentration index in global ASDR of 11 cancer subtypes in 1990 and 2021. 87

Table S14. Frontier analysis based on SDI and total cancer related ASDR attributable to high BMI among older people in 204 countries and territories in 2021. 88

Figure S1 Joinpoint regression analysis of ASDR for total cancer attributable to high BMI among older people from 1990 to 2021 across 21 GBD regional levels. 97

Table S1: The Socio-demographic Index (SDI) reference values from the Global Burden of Disease data in 2021.

Location name	Lower bound	Upper bound
Low SDI	0	0.46581580319161997
Low-middle SDI	0.46581580319161997	0.6188294452454329
Middle SDI	0.6188294452454329	0.7119746219361235
High-middle SDI	0.7119746219361235	0.8102959891918925
High SDI	0.8102959891918925	1

Table S2. The Socio-demographic Index (SDI) values of 204 countries/territories in 2021.

Location	SDI	SDI level
Somalia	0.077688109	Low SDI
Niger	0.168072774	Low SDI
Chad	0.240436019	Low SDI
Mali	0.268579941	Low SDI
South Sudan	0.278371125	Low SDI
Burkina Faso	0.285118402	Low SDI
Burundi	0.289374365	Low SDI
Central African Republic	0.30916769	Low SDI
Mozambique	0.326462614	Low SDI
Guinea	0.336401293	Low SDI
Afghanistan	0.337199998	Low SDI
Liberia	0.352442452	Low SDI
Guinea-Bissau	0.353109621	Low SDI
Sierra Leone	0.358665881	Low SDI
Ethiopia	0.358823295	Low SDI
Benin	0.373486574	Low SDI
Democratic Republic of the Congo	0.383179849	Low SDI
Malawi	0.384553634	Low SDI
Madagascar	0.400246943	Low SDI
Eritrea	0.403863943	Low SDI
Senegal	0.408054193	Low SDI
Togo	0.408533695	Low SDI

Location	SDI	SDI level
Gambia	0.40971416	Low SDI
Papua New Guinea	0.417797443	Low SDI
Uganda	0.423261181	Low SDI
Coted'Ivoire	0.425941883	Low SDI
Solomon Islands	0.429360316	Low SDI
Nepal	0.433174635	Low SDI
Rwanda	0.435588706	Low SDI
Timor-Leste	0.444667619	Low SDI
United Republic of Tanzania	0.446568273	Low SDI
Haiti	0.448278285	Low SDI
Yemen	0.450376375	Low SDI
Angola	0.453721949	Low SDI
Bhutan	0.473062378	Low-middle SDI
Vanuatu	0.473100706	Low-middle SDI
Cambodia	0.473621491	Low-middle SDI
Zimbabwe	0.473819486	Low-middle SDI
Comoros	0.475978688	Low-middle SDI
Cameroon	0.479691223	Low-middle SDI
Djibouti	0.487958371	Low-middle SDI
Lao People's Democratic Republic	0.489136091	Low-middle SDI
Bangladesh	0.492420885	Low-middle SDI
Mauritania	0.4989451	Low-middle SDI
Nigeria	0.503390833	Low-middle SDI

Location	SDI	SDI level
Pakistan	0.504028689	Low-middle SDI
Sao Tome and Principe	0.505413747	Low-middle SDI
Zambia	0.505948954	Low-middle SDI
Lesotho	0.510393066	Low-middle SDI
Honduras	0.513037248	Low-middle SDI
Kenya	0.523768077	Low-middle SDI
Nicaragua	0.523958472	Low-middle SDI
Kiribati	0.527186583	Low-middle SDI
Cabo Verde	0.533534539	Low-middle SDI
Myanmar	0.53390084	Low-middle SDI
Guatemala	0.539972424	Low-middle SDI
Tajikistan	0.541511187	Low-middle SDI
Sudan	0.541949735	Low-middle SDI
Morocco	0.562698301	Low-middle SDI
El Salvador	0.563775188	Low-middle SDI
Ghana	0.56493039	Low-middle SDI
Democratic People's Republic of Korea	0.569854634	Low-middle SDI
Marshall Islands	0.574091128	Low-middle SDI
India	0.575401649	Low-middle SDI
Tuvalu	0.576620529	Low-middle SDI
Congo	0.583075236	Low-middle SDI
Eswatini	0.585459713	Low-middle SDI
Micronesia (Federated States of)	0.587534967	Low-middle SDI

Location	SDI	SDI level
Samoa	0.593392769	Low-middle SDI
Venezuela (Bolivarian Republic of)	0.596513059	Low-middle SDI
Bolivia (Plurinational State of)	0.599010799	Low-middle SDI
Kyrgyzstan	0.603979328	Low-middle SDI
Egypt	0.606787094	Low-middle SDI
Belize	0.610229002	Low-middle SDI
Namibia	0.617564872	Low-middle SDI
Mongolia	0.617621565	Low-middle SDI
Dominican Republic	0.619388201	Middle SDI
Syrian Arab Republic	0.623004075	Middle SDI
Nauru	0.625177834	Middle SDI
Tonga	0.626349936	Middle SDI
Viet Nam	0.627933721	Middle SDI
Palestine	0.631011665	Middle SDI
Suriname	0.633665739	Middle SDI
Gabon	0.634691393	Middle SDI
Paraguay	0.635718099	Middle SDI
Saint Vincent and the Grenadines	0.637195963	Middle SDI
Botswana	0.642721629	Middle SDI
Guyana	0.650812335	Middle SDI
Maldives	0.650886627	Middle SDI
Philippines	0.651219329	Middle SDI
Brazil	0.653043887	Middle SDI

Location	SDI	SDI level
Colombia	0.655442913	Middle SDI
Indonesia	0.656868336	Middle SDI
Equatorial Guinea	0.657857456	Middle SDI
Algeria	0.659500924	Middle SDI
Ecuador	0.661017053	Middle SDI
Peru	0.662054037	Middle SDI
Uzbekistan	0.662621694	Middle SDI
Iraq	0.662626231	Middle SDI
Mexico	0.664575304	Middle SDI
Cuba	0.668729864	Middle SDI
Grenada	0.668993028	Middle SDI
Saint Lucia	0.672509735	Middle SDI
Fiji	0.675051631	Middle SDI
South Africa	0.679626598	Middle SDI
Turkmenistan	0.682160776	Middle SDI
Tunisia	0.682432216	Middle SDI
Thailand	0.682547933	Middle SDI
Jamaica	0.683263064	Middle SDI
Tokelau	0.686425621	Middle SDI
Azerbaijan	0.694851274	Middle SDI
Iran (Islamic Republic of)	0.697207398	Middle SDI
Costa Rica	0.700340477	Middle SDI
Sri Lanka	0.701534935	Middle SDI

Location	SDI	SDI level
Armenia	0.701833194	Middle SDI
Albania	0.706849791	Middle SDI
Panama	0.708864828	Middle SDI
Turkey	0.712692673	High-middle SDI
Mauritius	0.718260446	High-middle SDI
Uruguay	0.719283445	High-middle SDI
China	0.72162976	High-middle SDI
Bosnia and Herzegovina	0.723077893	High-middle SDI
Argentina	0.723122973	High-middle SDI
American Samoa	0.723727533	High-middle SDI
Kazakhstan	0.725144495	High-middle SDI
Jordan	0.725307227	High-middle SDI
Libya	0.725771399	High-middle SDI
Niue	0.72622205	High-middle SDI
Seychelles	0.730150775	High-middle SDI
Republic of Moldova	0.732214875	High-middle SDI
Georgia	0.732473604	High-middle SDI
Malaysia	0.742523828	High-middle SDI
Portugal	0.744151851	High-middle SDI
Lebanon	0.744746351	High-middle SDI
Barbados	0.746748764	High-middle SDI
Dominica	0.746967185	High-middle SDI
Antigua and Barbuda	0.749886887	High-middle SDI

Location	SDI	SDI level
North Macedonia	0.750629703	High-middle SDI
Bahrain	0.753043204	High-middle SDI
Palau	0.754046931	High-middle SDI
Saint Kitts and Nevis	0.754987055	High-middle SDI
Ukraine	0.760773913	High-middle SDI
Bulgaria	0.768150939	High-middle SDI
Romania	0.768453864	High-middle SDI
Trinidad and Tobago	0.768763254	High-middle SDI
Spain	0.769283698	High-middle SDI
Chile	0.771514716	High-middle SDI
Northern Mariana Islands	0.771535213	High-middle SDI
Oman	0.773391602	High-middle SDI
Cook Islands	0.779109955	High-middle SDI
Belarus	0.784484711	High-middle SDI
Hungary	0.790754768	High-middle SDI
Greece	0.791854408	High-middle SDI
Serbia	0.792416294	High-middle SDI
Montenegro	0.795800584	High-middle SDI
Croatia	0.798341027	High-middle SDI
Malta	0.801585034	High-middle SDI
Guam	0.803982203	High-middle SDI
Bahamas	0.805020668	High-middle SDI
Italy	0.805773534	High-middle SDI

Location	SDI	SDI level
Russian Federation	0.808536005	High-middle SDI
Israel	0.809011652	High-middle SDI
Brunei Darussalam	0.810234367	High-middle SDI
Slovakia	0.81061053	High SDI
Poland	0.812042809	High SDI
Saudi Arabia	0.815143493	High SDI
Bermuda	0.821365422	High SDI
United States Virgin Islands	0.821830853	High SDI
Puerto Rico	0.825525847	High SDI
Greenland	0.826210336	High SDI
Czechia	0.828450433	High SDI
Latvia	0.830663516	High SDI
Cyprus	0.835630545	High SDI
France	0.838364875	High SDI
Slovenia	0.842430731	High SDI
Australia	0.844252814	High SDI
Estonia	0.844917787	High SDI
Kuwait	0.846651055	High SDI
Qatar	0.846860584	High SDI
United Arab Emirates	0.849317734	High SDI
New Zealand	0.849442499	High SDI
Belgium	0.853654016	High SDI
Austria	0.853837004	High SDI

**Location**	**SDI**	**SDI level**
Singapore	0.856097766	High SDI
Lithuania	0.856484049	High SDI
United Kingdom	0.859000182	High SDI
Finland	0.859831368	High SDI
United States of America	0.862448354	High SDI
Andorra	0.869444113	High SDI
Japan	0.871241813	High SDI
Canada	0.87317068	High SDI
Ireland	0.87375385	High SDI
Taiwan (Province of China)	0.874747053	High SDI
Iceland	0.87636168	High SDI
Luxembourg	0.884428955	High SDI
Republic of Korea	0.886675267	High SDI
Sweden	0.886880299	High SDI
San Marino	0.888005474	High SDI
Netherlands	0.888464256	High SDI
Denmark	0.896424204	High SDI
Germany	0.902957091	High SDI
Monaco	0.908262831	High SDI
Norway	0.91613281	High SDI
Switzerland	0.933059111	High SDI

Table S3. International Classification of Diseases (ICD) diagnostic criteria for 11 types of cancers in the Global Burden of Disease (GBD) data.

Cancers	ICD10	ICD9
Breast cancer	C50-C50.9, D05-D05.9, D24-D24.9, D48.6, D49.3	174-175.9, 217-217.8, 233.0, 238.3, 239.3, 610-610.9
Colon and rectum cancer	C18-C21.9, D01.0-D01.3, D12-D12.9, D37.3-D37.5	153-154.9, 209.1, 209.5, 211.3-211.4, 230.3-230.6, 569.0
Gallbladder and biliary tract cancer	C23-C24.9, D13.5	156-156.9
Kidney cancer	C64-C65.9, D30.0-D30.1, D41.0-D41.1	189.0-189.1, 189.5-189.6, 223.0-223.1
Leukemia	C91-C93.7, C93.9-C95.2, C95.7-C95.92	204-208.92, V10.59-V10.69, V16.6
Liver cancer	C22-C22.8, D13.4	155-155.1, 155.3-155.9, 211.5
Multiple myeloma	C88-C90.32	203-203.9
Non-Hodgkin lymphoma	C82-C85.29, C85.7-C86.6, C96-C96.9	200-200.9, 202-202.98
Ovarian cancer	C56-C56.9, D27-D27.9, D39.1	183-183.0, 220-220.9, 236.2
Thyroid cancer	C73-C73.9, D09.3, D09.8, D34-D34.9, D44.0	193-193.9, 226-226.9
Uterine cancer	C54-C54.9, D07.0-D07.2, D26.1-D26.9	182-182.9, 233.2

List of International Classification of Diseases (ICD) codes mapped to the Global Burden of Diseases cause list for causes of death.

Table S4: The correspondence of Global Burden of Disease (GBD) regions with 204 countries/territories in 2021.

21 GBD regions	204 countries/territories
High-income Asia Pacific	Brunei Darussalam, Singapore, Japan, Republic of Korea
High-income North America	Greenland, United States of America, Canada
Western Europe	Portugal, Spain, Greece, Malta, Italy, Israel, Cyprus, France, Belgium, Austria, United Kingdom, Finland, Andorra, Ireland, Iceland, Luxembourg, Sweden, San Marino, Netherlands, Denmark, Germany, Monaco, Norway, Switzerland
Australasia	Australia, New Zealand
Eastern Europe	Republic of Moldova, Ukraine, Belarus, Russian Federation, Latvia, Estonia, Lithuania
Central Europe	Albania, Bosnia and Herzegovina, North Macedonia, Bulgaria, Romania, Hungary, Serbia, Montenegro, Croatia, Slovakia, Poland, Czechia, Slovenia
Southern Latin America	Uruguay, Argentina, Chile
East Asia	China, Democratic People's Republic of Korea, Taiwan (Province of China)
Central Asia	Tajikistan, Kyrgyzstan, Mongolia, Uzbekistan, Turkmenistan, Azerbaijan, Armenia, Kazakhstan, Georgia
North Africa and Middle East	Afghanistan, Yemen, Sudan, Morocco, Egypt, Syrian Arab Republic, Palestine, Algeria, Iraq, Tunisia, Iran (Islamic Republic of), Turkey, Jordan, Libya, Lebanon, Bahrain, Oman, Saudi Arabia, Kuwait, Qatar, United Arab Emirates
Andean Latin America	Bolivia (Plurinational State of), Ecuador, Peru
Southeast Asia	Timor-Leste, Cambodia, Lao People's Democratic Republic, Myanmar, Viet Nam, Maldives, Philippines, Indonesia, Thailand, Sri Lanka, Mauritius, Seychelles, Malaysia
Tropical Latin America	Paraguay, Brazil
Southern Sub-Saharan Africa	Zimbabwe, Lesotho, Eswatini, Namibia, Botswana, South Africa
Caribbean	Haiti, Belize, Dominican Republic, Suriname, Saint Vincent and the Grenadines, Guyana, Cuba, Grenada, Saint Lucia, Jamaica, Barbados, Dominica, Antigua and Barbuda, Saint Kitts and Nevis, Trinidad and Tobago, Bahamas, Bermuda, United States Virgin Islands, Puerto Rico
Central Latin America	Honduras, Nicaragua, Guatemala, El Salvador, Venezuela (Bolivarian Republic of), Colombia, Mexico, Costa Rica, Panama
South Asia	Nepal, Bhutan, Bangladesh, Pakistan, India

21 GBD regions	204 countries/territories
Central Sub-Saharan Africa	Central African Republic, Democratic Republic of the Congo, Angola, Congo, Gabon, Equatorial Guinea
Oceania	Papua New Guinea, Solomon Islands, Vanuatu, Kiribati, Marshall Islands, Tuvalu, Micronesia (Federated States of), Samoa, Nauru, Tonga, Fiji, Tokelau, American Samoa, Niue, Palau, Northern Mariana Islands, Cook Islands, Guam
Western Sub-Saharan Africa	Niger, Chad, Mali, Burkina Faso, Guinea, Liberia, Guinea-Bissau, Sierra Leone, Benin, Senegal, Togo, Coted'Ivoire, Gambia, Cameroon, Mauritania, Nigeria, Sao Tome and Principe, Cabo Verde, Ghana
Eastern Sub-Saharan Africa	Somalia, South Sudan, Burundi, Mozambique, Ethiopia, Malawi, Madagascar, Eritrea, Uganda, Rwanda, United Republic of Tanzania, Comoros, Djibouti, Zambia, Kenya

Table S5. Proportion of DALYs for total cancer attributable to high body mass index from 1990 to 2021 across four continental levels.

Year	Asia		America		Europe		Africa	
	DALYs	Percent (%)	DALYs	Percent (%)	DALYs	Percent (%)	DALYs	Percent (%)
1990	346,110 (167,848 to 552,443)	16.49	585,379 (219,787 to 976,001)	27.88	1,099,516 (435,642 to 1,817,419)	52.37	68,365 (26,184 to 111,419)	3.26
1991	363,764 (174,528 to 576,276)	16.74	602,546 (226,105 to 1,003,197)	27.73	1,134,914 (449,483 to 1,874,448)	52.24	71,362 (27,268 to 116,274)	3.28
1992	382,198 (183,881 to 608,567)	17.03	619,371 (233,239 to 1,029,833)	27.60	1,167,966 (462,189 to 1,928,973)	52.04	74,875 (28,856 to 122,195)	3.34
1993	401,663 (193,493 to 640,408)	17.27	639,166 (240,669 to 1,063,990)	27.49	1,206,204 (476,423 to 1,991,763)	51.87	78,397 (30,105 to 128,491)	3.37
1994	423,059 (200,674 to 674,328)	17.66	657,132 (247,454 to 1,094,258)	27.43	1,232,759 (486,238 to 2,035,044)	51.46	82,486 (31,421 to 134,094)	3.44
1995	446,487 (210,257 to 704,619)	18.21	672,899 (253,690 to 1,121,170)	27.44	1,247,255 (490,447 to 2,057,305)	50.86	85,529 (32,528 to 139,548)	3.49
1996	468,760 (218,494 to 741,142)	18.72	683,759 (258,143 to 1,137,857)	27.31	1,261,088 (494,980 to 2,079,810)	50.37	90,093 (34,400 to 146,614)	3.60
1997	490,547 (227,047 to 778,490)	19.14	695,750 (263,859 to 1,157,710)	27.15	1,281,043 (502,715 to 2,111,677)	49.98	95,668 (36,534 to 155,528)	3.73
1998	515,960 (241,217 to 809,849)	19.57	714,118 (271,631 to 1,186,426)	27.09	1,306,492 (512,309 to 2,151,056)	49.55	99,918 (38,209 to 163,018)	3.79
1999	540,614 (246,860 to 858,116)	19.83	737,238 (281,377 to 1,222,441)	27.04	1,344,214 (527,974 to 2,210,583)	49.31	104,055 (39,958 to 169,517)	3.82

Year	Asia		America		Europe		Africa	
	DALYs	Percent (%)	DALYs	Percent (%)	DALYs	Percent (%)	DALYs	Percent (%)
2000	567,896 (258,704 to 904,111)	20.27	755,755 (288,098 to 1,251,523)	26.97	1,370,025 (535,755 to 2,256,256)	48.89	108,615 (41,674 to 176,659)	3.88
2001	593,485 (266,884 to 935,566)	20.69	773,117 (295,587 to 1,279,808)	26.95	1,387,418 (540,303 to 2,283,322)	48.37	114,266 (43,929 to 185,670)	3.98
2002	618,820 (277,095 to 979,194)	21.05	793,657 (304,019 to 1,314,283)	27.00	1,406,149 (546,659 to 2,315,644)	47.84	120,802 (46,534 to 196,371)	4.11
2003	647,480 (292,013 to 1,020,417)	21.51	814,751 (312,423 to 1,349,641)	27.07	1,420,242 (551,825 to 2,339,710)	47.18	127,738 (48,870 to 207,280)	4.24
2004	676,446 (301,469 to 1,070,304)	22.15	825,005 (317,015 to 1,368,243)	27.02	1,417,860 (549,064 to 2,339,601)	46.44	133,991 (50,948 to 218,295)	4.39
2005	704,617 (311,534 to 1,115,017)	22.65	841,762 (323,110 to 1,397,278)	27.05	1,425,278 (550,332 to 2,357,462)	45.81	139,909 (52,892 to 228,608)	4.50
2006	726,906 (320,434 to 1,149,880)	23.04	861,516 (331,208 to 1,430,030)	27.31	1,419,231 (545,503 to 2,351,206)	44.99	146,881 (55,617 to 240,359)	4.66
2007	765,158 (338,192 to 1,214,234)	23.62	886,480 (341,512 to 1,470,728)	27.36	1,435,637 (549,864 to 2,380,847)	44.31	152,814 (57,276 to 250,306)	4.72
2008	811,877 (353,120 to 1,291,370)	24.16	916,486 (353,723 to 1,522,896)	27.27	1,471,350 (562,418 to 2,442,254)	43.78	160,822 (60,071 to 262,896)	4.79
2009	864,406 (369,328 to 1,372,623)	24.86	949,070 (367,779 to 1,574,550)	27.29	1,493,734 (570,642 to 2,481,482)	42.95	170,279 (63,840 to 280,226)	4.90
2010	918,794 (394,534 to 1,471,175)	25.47	978,932 (380,101 to 1,624,428)	27.14	1,529,424 (583,923 to 2,543,112)	42.40	179,570 (67,046 to 294,484)	4.98

Year	Asia		America		Europe		Africa	
	DALYs	Percent (%)	DALYs	Percent (%)	DALYs	Percent (%)	DALYs	Percent (%)
2011	976,234 (418,524 to 1,558,105)	26.13	1,014,046 (394,445 to 1,681,408)	27.14	1,555,641 (595,145 to 2,586,285)	41.64	190,102 (70,513 to 312,128)	5.09
2012	1,028,804 (441,089 to 1,644,832)	26.57	1,049,264 (409,211 to 1,742,379)	27.10	1,592,320 (610,477 to 2,646,495)	41.13	201,252 (74,595 to 332,300)	5.20
2013	1,085,768 (465,107 to 1,731,056)	27.07	1,086,829 (425,493 to 1,804,737)	27.10	1,626,782 (625,172 to 2,705,819)	40.57	210,851 (77,845 to 346,988)	5.26
2014	1,148,632 (486,867 to 1,840,286)	27.62	1,127,085 (442,443 to 1,870,265)	27.10	1,657,445 (636,045 to 2,759,537)	39.86	225,102 (83,245 to 369,274)	5.41
2015	1,222,961 (510,802 to 1,952,689)	28.16	1,169,374 (458,818 to 1,940,041)	26.92	1,709,998 (657,824 to 2,847,348)	39.37	240,885 (89,225 to 398,090)	5.55
2016	1,302,007 (557,125 to 2,090,728)	28.74	1,227,049 (483,230 to 2,032,280)	27.08	1,748,005 (672,793 to 2,909,444)	38.58	253,432 (94,510 to 416,661)	5.59
2017	1,380,265 (583,160 to 2,251,822)	29.40	1,267,367 (500,143 to 2,097,041)	26.99	1,783,425 (687,452 to 2,966,845)	37.98	264,450 (98,124 to 435,462)	5.63
2018	1,462,254 (604,889 to 2,399,594)	29.95	1,307,760 (517,457 to 2,161,143)	26.79	1,833,141 (708,853 to 3,042,064)	37.55	279,207 (103,809 to 459,653)	5.72
2019	1,544,804 (642,390 to 2,550,387)	30.56	1,353,023 (536,984 to 2,230,862)	26.77	1,863,129 (725,273 to 3,097,262)	36.86	293,237 (108,381 to 482,894)	5.80
2020	1,612,126 (661,369 to 2,636,482)	31.05	1,392,294 (553,077 to 2,292,551)	26.82	1,875,812 (725,209 to 3,130,454)	36.13	310,978 (114,369 to 513,662)	5.99
2021	1,700,997 (707,180 to 2,763,100)	31.60	1,439,257 (572,914 to 2,374,024)	26.74	1,916,666 (740,491 to 3,195,552)	35.61	325,975 (120,380 to 544,055)	6.06

Table S6. Proportion of DALYs for total cancer attributable to high body mass index from 1990 to 2021 across five SDI regional levels.

Year	High SDI		High-middle SDI		Middle SDI		Low-middle SDI		Low SDI	
	DALYs	Percent (%)	DALYs	Percent (%)	DALYs	Percent (%)	DALYs	Percent (%)	DALYs	Percent (%)
1990	1,095,387 (417,054 to 1,824,885)	52.10	681,967 (285,637 to 1,116,198)	32.44	212,357 (97,144 to 342,250)	10.10	83,933 (38,268 to 132,586)	3.99	28,651 (12,374 to 45,954)	1.36
1991	1,123,303 (426,840 to 1,868,007)	51.63	711,424 (297,068 to 1,160,435)	32.70	223,100 (101,814 to 354,963)	10.25	88,113 (39,927 to 140,034)	4.05	29,657 (12,604 to 47,614)	1.36
1992	1,146,898 (436,178 to 1,906,350)	51.03	741,158 (309,887 to 1,207,631)	32.98	236,002 (107,528 to 377,550)	10.50	92,819 (42,053 to 147,492)	4.13	30,702 (13,006 to 49,148)	1.37
1993	1,172,672 (444,145 to 1,949,993)	50.36	778,890 (323,984 to 1,268,494)	33.45	247,834 (112,243 to 395,750)	10.64	97,491 (44,149 to 155,069)	4.19	31,881 (13,423 to 50,940)	1.37
1994	1,197,638 (452,470 to 1,992,679)	49.92	804,143 (332,428 to 1,309,522)	33.52	261,045 (115,862 to 418,385)	10.88	102,936 (46,425 to 163,711)	4.29	33,133 (13,913 to 53,384)	1.38
1995	1,222,143 (460,901 to 2,030,766)	49.76	818,667 (337,739 to 1,332,951)	33.34	273,917 (121,191 to 438,111)	11.15	106,886 (47,809 to 170,763)	4.35	34,256 (14,214 to 54,838)	1.39
1996	1,237,592 (467,580 to 2,054,897)	49.36	835,135 (344,580 to 1,360,367)	33.31	288,001 (126,510 to 460,846)	11.49	111,438 (49,924 to 179,363)	4.44	35,360 (14,688 to 57,125)	1.41
1997	1,253,414 (473,843 to 2,080,558)	48.83	856,781 (353,856 to 1,390,180)	33.38	302,836 (132,117 to 487,125)	11.80	117,259 (52,592 to 188,261)	4.57	36,532 (15,157 to 58,374)	1.42
1998	1,279,146 (483,510 to 2,122,217)	48.44	882,075 (364,414 to 1,429,321)	33.40	317,992 (139,086 to 510,044)	12.04	123,513 (55,014 to 198,302)	4.68	37,864 (15,644 to 61,250)	1.43
1999	1,306,954 (493,616 to 2,170,026)	47.87	922,551 (381,422 to 1,494,854)	33.79	332,581 (143,514 to 537,407)	12.18	129,218 (57,013 to 207,217)	4.73	38,998 (16,030 to 62,827)	1.43

Year	High SDI		High-middle SDI		Middle SDI		Low-middle SDI		Low SDI	
	DALYs	Percent (%)	DALYs	Percent (%)	DALYs	Percent (%)	DALYs	Percent (%)	DALYs	Percent (%)
2000	1,329,970 (502,291 to 2,209,859)	47.39	951,254 (392,418 to 1,544,806)	33.89	349,247 (149,878 to 563,763)	12.44	135,962 (59,683 to 218,882)	4.84	40,298 (16,478 to 65,628)	1.44
2001	1,352,589 (510,682 to 2,246,131)	47.08	968,798 (396,327 to 1,576,547)	33.72	365,494 (155,833 to 586,391)	12.72	144,471 (63,374 to 232,264)	5.03	41,608 (16,970 to 67,164)	1.45
2002	1,376,634 (519,241 to 2,286,597)	46.76	986,466 (403,500 to 1,598,721)	33.51	383,452 (163,419 to 618,683)	13.02	154,348 (67,589 to 247,499)	5.24	43,159 (17,589 to 70,130)	1.47
2003	1,399,997 (528,160 to 2,323,021)	46.43	1,002,854 (410,395 to 1,625,517)	33.26	402,724 (172,570 to 646,117)	13.36	164,672 (71,381 to 265,202)	5.46	44,842 (18,188 to 72,335)	1.49
2004	1,411,993 (532,991 to 2,345,473)	46.17	1,006,093 (409,228 to 1,640,671)	32.90	420,378 (178,138 to 675,738)	13.75	173,421 (74,929 to 278,626)	5.67	46,476 (18,693 to 74,465)	1.52
2005	1,428,802 (539,182 to 2,373,036)	45.84	1,019,656 (417,046 to 1,661,176)	32.72	437,240 (184,740 to 705,963)	14.03	182,943 (78,678 to 292,433)	5.87	48,082 (19,583 to 77,061)	1.54
2006	1,447,570 (546,420 to 2,402,801)	45.81	1,015,122 (411,263 to 1,659,909)	32.13	453,855 (190,109 to 731,708)	14.36	193,329 (83,249 to 309,663)	6.12	49,925 (20,163 to 80,317)	1.58
2007	1,480,334 (559,981 to 2,456,295)	45.61	1,033,009 (414,037 to 1,684,302)	31.83	476,248 (200,231 to 766,785)	14.67	204,078 (87,217 to 329,003)	6.29	51,900 (20,822 to 82,980)	1.60
2008	1,521,252 (575,777 to 2,527,128)	45.19	1,066,017 (428,031 to 1,740,497)	31.67	508,164 (211,006 to 821,398)	15.10	216,637 (92,239 to 350,474)	6.44	54,113 (21,883 to 86,161)	1.61
2009	1,557,739 (591,377 to 2,584,812)	44.72	1,096,183 (437,135 to 1,789,160)	31.47	542,866 (222,322 to 878,990)	15.58	230,096 (97,986 to 372,989)	6.61	56,561 (22,797 to 90,840)	1.62
2010	1,595,499 (607,429 to 2,644,396)	44.16	1,138,671 (455,430 to 1,864,727)	31.52	576,001 (236,152 to 939,937)	15.94	243,594 (103,036 to 396,148)	6.74	58,849 (23,682 to 94,347)	1.63

Year	High SDI		High-middle SDI		Middle SDI		Low-middle SDI		Low SDI	
	DALYs	Percent (%)	DALYs	Percent (%)	DALYs	Percent (%)	DALYs	Percent (%)	DALYs	Percent (%)
2011	1,638,188 (624,784 to 2,711,639)	43.77	1,173,029 (467,671 to 1,911,204)	31.34	609,811 (251,699 to 987,615)	16.29	259,286 (109,897 to 421,175)	6.93	62,087 (24,900 to 99,916)	1.66
2012	1,676,673 (640,784 to 2,773,173)	43.24	1,214,518 (486,446 to 1,978,954)	31.32	645,504 (266,929 to 1,047,630)	16.65	275,624 (116,239 to 447,774)	7.11	65,556 (26,225 to 104,609)	1.69
2013	1,716,848 (658,045 to 2,839,072)	42.74	1,254,115 (501,307 to 2,047,234)	31.22	686,913 (286,109 to 1,113,219)	17.10	289,669 (123,235 to 465,301)	7.21	69,225 (27,683 to 110,982)	1.72
2014	1,757,609 (674,267 to 2,907,042)	42.20	1,295,515 (511,907 to 2,116,048)	31.11	731,545 (300,861 to 1,186,308)	17.56	307,209 (130,439 to 496,547)	7.38	73,027 (28,844 to 118,556)	1.75
2015	1,813,620 (694,960 to 3,000,811)	41.69	1,347,264 (535,430 to 2,200,638)	30.97	781,973 (319,545 to 1,262,237)	17.98	329,987 (137,757 to 534,909)	7.59	76,899 (30,223 to 125,022)	1.77
2016	1,872,113 (719,715 to 3,095,386)	41.26	1,397,163 (558,859 to 2,294,934)	30.79	836,909 (347,674 to 1,360,691)	18.44	350,251 (150,009 to 571,738)	7.72	80,916 (31,742 to 132,961)	1.78
2017	1,918,340 (739,194 to 3,169,539)	40.79	1,441,133 (578,402 to 2,359,643)	30.65	888,659 (364,699 to 1,443,735)	18.90	368,967 (155,717 to 601,744)	7.85	85,423 (33,241 to 141,077)	1.82
2018	1,963,980 (756,644 to 3,248,924)	40.17	1,498,378 (595,195 to 2,478,810)	30.65	944,799 (380,350 to 1,565,636)	19.32	392,146 (164,424 to 637,936)	8.02	90,000 (35,340 to 148,403)	1.84
2019	2,002,981 (772,911 to 3,308,646)	39.57	1,546,915 (618,138 to 2,561,279)	30.56	1,003,488 (408,247 to 1,653,022)	19.83	413,258 (172,662 to 675,159)	8.16	94,864 (37,232 to 155,912)	1.87
2020	2,025,214 (780,079 to 3,346,940)	38.96	1,584,020 (624,174 to 2,619,920)	30.47	1,056,814 (428,320 to 1,730,995)	20.33	432,824 (177,573 to 710,235)	8.33	99,780 (38,447 to 166,117)	1.92
2021	2,088,693 (806,778 to 3,457,569)	38.75	1,638,810 (653,959 to 2,701,854)	30.40	1,109,676 (453,532 to 1,834,919)	20.59	449,316 (184,706 to 736,548)	8.34	104,123 (40,372 to 172,587)	1.93

Table S7. Cancer-related disability-adjusted life years and age-standardized DALYs rates attributable to high BMI among older people among 204 countries and territories in 1990 and 2021, with average annual percent change from 1990 to 2021.

Location	DALYs in 1990 (95%CI)	DALYs in 2021 (95%CI)	ASDR in 1990 (per 100,000 population,95%CI)	ASDR in 2021 (per 100,000 population, 95%CI)	AAPC of ASDR (95%CI)
Afghanistan	2,647 (1,084 to 4,746)	3,784 (1,630 to 6,401)	293.12(121.96 to 526.16)	467.07(200.1 to 794.22)	1.17(1.07 to 1.28)
Albania	1,262 (556 to 2,152)	3,234 (1,249 to 5,694)	529.02(233.38 to 901.29)	545.21(211.05 to 960.63)	0.11(-0.33 to 0.56)
Algeria	2,578 (1,155 to 4,329)	12,610 (5,220 to 21,379)	185.65(84.63 to 312.48)	311.13(130.63 to 526.85)	1.5(1.45 to 1.55)
American Samoa	17 (6 to 29)	58 (22 to 96)	664.29(246.85 to 1122.53)	1005.68(379.02 to 1683.23)	1.52(1.38 to 1.66)
Andorra	58 (21 to 108)	137 (50 to 261)	786.68(283.88 to 1460.66)	703.32(257.56 to 1340.05)	-0.51(-0.88 to -0.13)
Angola	483 (161 to 960)	3,220 (1,005 to 6,456)	106.65(35.65 to 212.09)	241.61(74.67 to 487.88)	2.5(2.34 to 2.66)
Antigua and Barbuda	32 (11 to 55)	113 (40 to 190)	462.99(163.98 to 797.5)	843.63(298.14 to 1424.97)	1.5(1.06 to 1.95)
Argentina	37,621 (14,646 to 64,079)	73,609 (28,447 to 122,584)	899.98(350.51 to 1533.73)	1020.27(394.31 to 1698.11)	0.42(0.29 to 0.54)
Armenia	2,623 (981 to 4,371)	5,608 (2,171 to 9,383)	781.98(294.52 to 1306.12)	939.57(364.68 to 1570.84)	0.34(-0.21 to 0.9)
Australia	19,690 (7,024 to 33,404)	50,522 (18,693 to 84,808)	762.45(271.53 to 1293.96)	841.41(311.43 to 1410.76)	0.22(0.01 to 0.44)
Austria	14,016 (5,266 to 23,846)	16,317 (5,931 to 28,691)	887.48(332.59 to 1511.07)	689.87(251.05 to 1210.32)	-0.96(-1.19 to -0.74)
Azerbaijan	2,877 (1,172 to 4,759)	8,275 (3,233 to 14,919)	490.27(198.83 to 811.84)	633.03(246.99 to 1148.04)	0.52(0.27 to 0.78)
Bahamas	121 (38 to 212)	505 (156 to 881)	681.03(214.04 to 1188.91)	1022.41(315.64 to 1784.49)	1.22(0.91 to 1.52)
Bahrain	128 (48 to 219)	844 (287 to 1,458)	716.19(272.68 to 1221.84)	955.98(331.86 to 1641.78)	0.6(0.39 to 0.8)

Location	DALYs in 1990 (95%CI)	DALYs in 2021 (95%CI)	ASDR in 1990 (per 100,000 population,95%CI)	ASDR in 2021 (per 100,000 population, 95%CI)	AAPC of ASDR (95%CI)
Bangladesh	1,770 (935 to 2,956)	11,527 (5,172 to 20,796)	32.46(17.23 to 54.11)	67(30.18 to 120.61)	2.6(2.33 to 2.86)
Barbados	273 (100 to 471)	830 (312 to 1,438)	727.26(267.01 to 1255.36)	1192.72(447.41 to 2066.61)	1.43(1.18 to 1.67)
Belarus	10,342 (4,227 to 16,962)	22,313 (9,090 to 38,526)	604.74(247.13 to 994.34)	1013.43(413.53 to 1750.65)	1.52(1.08 to 1.96)
Belgium	15,010 (5,142 to 25,429)	21,934 (7,549 to 38,184)	735.27(251.03 to 1246.81)	709.15(244.18 to 1232.34)	-0.24(-0.48 to 0.01)
Belize	39 (17 to 63)	204 (89 to 325)	343.09(148.69 to 555.44)	581.53(253.24 to 930.17)	1.8(1.51 to 2.09)
Benin	512 (167 to 1,005)	1,726 (565 to 3,295)	211.05(68.74 to 414.23)	296.09(96.82 to 565.26)	0.85(0.64 to 1.06)
Bermuda	82 (28 to 144)	169 (63 to 289)	1055.42(356.17 to 1846.58)	920.9(346.21 to 1572.57)	-0.48(-0.61 to -0.35)
Bhutan	39 (17 to 69)	164 (68 to 311)	136.27(58.54 to 240.57)	223.02(93.13 to 421.7)	1.31(1.23 to 1.38)
Bolivia (Plurinational State of)	1,921 (877 to 3,373)	8,752 (3,627 to 15,362)	500.35(229.96 to 877.75)	775.65(322.39 to 1360.76)	1.18(1.08 to 1.28)
Bosnia and Herzegovina	3,056 (1,326 to 5,048)	7,920 (3,191 to 13,265)	620.12(271.74 to 1022.26)	932.97(375.88 to 1562.59)	1.18(0.94 to 1.42)
Botswana	187 (58 to 363)	921 (279 to 1,666)	282.34(88.52 to 547.82)	577.16(176.23 to 1035.4)	2.19(1.33 to 3.07)
Brazil	41,209 (16,863 to 68,142)	183,321 (70,230 to 307,786)	388.42(159.55 to 642.23)	580.12(222.21 to 973.81)	1.26(1.14 to 1.39)
Brunei Darussalam	41 (17 to 70)	246 (94 to 418)	366.89(153.63 to 633.11)	564.85(218.2 to 960.79)	1.37(1.25 to 1.49)
Bulgaria	13,605 (5,646 to 22,585)	22,213 (9,051 to 38,463)	805.28(334.12 to 1337.44)	1149.97(468.14 to 1989.07)	0.93(0.48 to 1.39)
Burkina Faso	437 (144 to 870)	1,162 (344 to 2,329)	82.65(26.79 to 164.31)	109.72(31.98 to 219.38)	0.98(0.77 to 1.19)

Location	DALYs in 1990 (95%CI)	DALYs in 2021 (95%CI)	ASDR in 1990 (per 100,000 population,95%CI)	ASDR in 2021 (per 100,000 population, 95%CI)	AAPC of ASDR (95%CI)
Burundi	278 (99 to 534)	653 (217 to 1,272)	100.44(35.89 to 193.13)	118.83(39.69 to 232.1)	0.38(0.18 to 0.57)
Cabo Verde	47 (16 to 89)	236 (87 to 434)	162.27(56.32 to 307.85)	448.64(164.8 to 824.51)	3.26(3.09 to 3.44)
Cambodia	555 (240 to 1,021)	2,843 (1,036 to 5,178)	101.35(44.2 to 185.51)	182.67(67.16 to 333.39)	1.67(1.6 to 1.74)
Cameroon	1,850 (651 to 3,451)	7,452 (2,442 to 14,055)	355.79(125.14 to 663.86)	542.72(177.88 to 1024.04)	1.2(1.11 to 1.3)
Canada	36,071 (13,184 to 61,556)	78,026 (30,144 to 130,674)	847.44(309.88 to 1446.78)	797.91(308.5 to 1335.11)	-0.21(-0.39 to -0.03)
Central African Republic	155 (51 to 290)	532 (173 to 989)	112.25(36.69 to 212.21)	215.28(69.28 to 405.61)	2(1.86 to 2.14)
Chad	407 (127 to 849)	1,283 (428 to 2,488)	119.33(37.3 to 248.64)	196.4(65.26 to 383.02)	1.5(1.43 to 1.56)
Chile	11,265 (5,347 to 18,117)	31,007 (13,298 to 50,373)	916.99(435.27 to 1475.59)	934.91(400.82 to 1518.71)	-0.05(-0.27 to 0.17)
China	133,171 (65,760 to 214,727)	807,134 (330,206 to 1,379,115)	132.16(65.06 to 213.01)	298.22(122.23 to 509.52)	2.33(2.17 to 2.5)
Colombia	7,852 (3,418 to 13,096)	38,142 (14,826 to 66,124)	385.22(167.76 to 640.87)	550.12(214.13 to 953.08)	1.16(0.89 to 1.42)
Comoros	34 (12 to 65)	194 (63 to 369)	150.95(54.67 to 281.73)	346.03(112.71 to 657.35)	2.63(2.41 to 2.86)
Congo	308 (101 to 582)	1,292 (396 to 2,383)	226.37(74.49 to 428.47)	433.51(130.66 to 807.65)	1.96(1.78 to 2.14)
Cook Islands	9 (3 to 16)	24 (7 to 43)	612.63(186.46 to 1096.85)	720.65(213.15 to 1271.71)	0.49(0.3 to 0.69)
Costa Rica	950 (417 to 1,592)	5,477 (2,157 to 9,362)	454.24(199.35 to 760.81)	784.62(308.84 to 1341.13)	1.92(1.66 to 2.18)
Coted'Ivoire	638 (162 to 1,233)	2,984 (677 to 5,913)	146.37(38.22 to 282.87)	245.55(56.24 to 485.12)	1.38(1.17 to 1.6)
Croatia	7,230 (2,897 to 12,133)	14,561 (5,668 to 24,481)	949.16(380.92 to 1594.83)	1201.96(468.06 to 2019.99)	0.54(0.24 to 0.84)

Location	DALYs in 1990 (95%CI)	DALYs in 2021 (95%CI)	ASDR in 1990 (per 100,000 population,95%CI)	ASDR in 2021 (per 100,000 population, 95%CI)	AAPC of ASDR (95%CI)
Cuba	5,024 (1,973 to 8,224)	17,112 (6,815 to 29,129)	391.61(153.62 to 640.99)	704.21(280.31 to 1198.83)	1.61(1.25 to 1.98)
Cyprus	556 (224 to 956)	1,736 (629 to 3,104)	555.28(222.94 to 953.85)	652.76(236.23 to 1166.12)	0.5(0.23 to 0.76)
Czechia	29,209 (12,481 to 47,361)	35,806 (14,988 to 60,324)	1576.55(674.07 to 2558.51)	1233.71(513.83 to 2076.87)	-1.05(-1.17 to -0.93)
Democratic People's Republic of Korea	1,730 (741 to 3,115)	7,674 (2,924 to 14,745)	97.98(42.08 to 176.44)	195.59(73.92 to 377.11)	1.72(1.68 to 1.76)
Democratic Republic of the Congo	1,994 (667 to 3,642)	9,676 (3,087 to 18,230)	104.94(35 to 193.6)	240.21(76.27 to 456.44)	2.74(2.68 to 2.8)
Denmark	7,467 (2,646 to 12,548)	11,590 (4,110 to 19,926)	702.32(247.66 to 1183.26)	728.48(258.82 to 1250.48)	-0.24(-0.48 to 0.01)
Djibouti	12 (4 to 23)	111 (36 to 220)	85.27(30.06 to 166.46)	164.5(52.86 to 324.57)	2.17(2.07 to 2.27)
Dominica	60 (21 to 102)	114 (42 to 196)	766.37(274.6 to 1303.32)	1073.47(397.7 to 1844.39)	1.09(0.98 to 1.2)
Dominican Republic	755 (314 to 1,324)	4,496 (1,726 to 8,032)	173.29(71.96 to 303.9)	370.1(142.03 to 661.37)	2.33(1.85 to 2.81)
Ecuador	2,466 (1,238 to 3,950)	13,167 (5,385 to 21,878)	398.59(200.12 to 639.24)	652.37(267.08 to 1084.43)	1.47(0.98 to 1.96)
Egypt	15,240 (6,136 to 28,087)	90,186 (35,882 to 151,134)	500.46(200.84 to 924.1)	1220.54(490.87 to 2040.39)	2.59(2.26 to 2.93)
El Salvador	1,110 (545 to 1,763)	4,076 (1,799 to 6,839)	311.6(153.17 to 494.88)	536.54(236.95 to 900.54)	1.8(1.24 to 2.36)
Equatorial Guinea	39 (13 to 74)	263 (74 to 512)	167.24(56.97 to 316.9)	492.62(140.67 to 952.86)	3.2(2.77 to 3.63)
Eritrea	98 (36 to 196)	501 (159 to 1,040)	83.72(30.14 to 167.12)	169.79(54.29 to 351.57)	2.27(2.2 to 2.34)
Estonia	2,352 (948 to 3,869)	3,767 (1,399 to 6,473)	880.25(355.29 to 1446.69)	1050.09(390.12 to 1801.7)	0.09(-0.19 to 0.37)
Eswatini	179 (67 to 309)	830 (275 to 1,586)	593.49(219.11 to 1030.59)	1359.93(451.08 to 2575.64)	3.03(2.8 to 3.27)

Location	DALYs in 1990 (95%CI)	DALYs in 2021 (95%CI)	ASDR in 1990 (per 100,000 population,95%CI)	ASDR in 2021 (per 100,000 population, 95%CI)	AAPC of ASDR (95%CI)
Ethiopia	3,771 (1,944 to 6,161)	8,589 (3,694 to 14,532)	163.48(83.17 to 267.4)	178.3(76.56 to 301.57)	-0.14(-0.24 to -0.05)
Fiji	196 (75 to 341)	788 (290 to 1,346)	513.11(194.07 to 892.45)	857.78(312.06 to 1469.82)	1.38(1.19 to 1.58)
Finland	6,890 (2,596 to 11,810)	12,727 (4,654 to 21,934)	729.11(274.1 to 1250.17)	737.62(269.16 to 1271.37)	-0.11(-0.39 to 0.16)
France	73,025 (27,004 to 122,237)	140,759 (50,313 to 244,283)	673.74(248.75 to 1129.7)	748.37(266.13 to 1299.73)	0.32(0.18 to 0.46)
Gabon	287 (91 to 526)	871 (269 to 1,577)	395.78(124.84 to 728.15)	714.68(220.53 to 1296.71)	1.8(1.6 to 1.99)
Gambia	87 (30 to 160)	450 (156 to 864)	210.69(72.96 to 388.4)	400.77(138.27 to 771.08)	2.12(1.04 to 3.21)
Georgia	5,135 (2,214 to 8,315)	7,437 (2,910 to 12,437)	621.5(268.35 to 1007.97)	925.68(362.09 to 1548.26)	1.11(0.29 to 1.94)
Germany	169,408 (64,639 to 284,560)	207,239 (73,260 to 362,192)	1025.23(389.37 to 1724.4)	815.43(289.1 to 1426.6)	-0.86(-1.09 to -0.62)
Ghana	964 (321 to 1,884)	6,915 (2,330 to 12,721)	133.49(44.77 to 260.6)	364.98(123.07 to 673.91)	2.61(2.49 to 2.73)
Greece	11,845 (4,497 to 20,390)	26,059 (9,500 to 45,126)	593.35(225.38 to 1021.98)	839.15(306.17 to 1447.22)	1.19(0.9 to 1.49)
Greenland	48 (16 to 82)	83 (29 to 147)	1352.1(464.34 to 2318.73)	951.3(336 to 1678.3)	-0.93(-1.34 to -0.52)
Grenada	44 (16 to 76)	124 (42 to 216)	480.02(170.95 to 835.65)	884.86(298.63 to 1542.28)	1.61(1.44 to 1.79)
Guam	42 (17 to 73)	125 (48 to 216)	492.36(188.24 to 850.09)	448.95(172.16 to 775.53)	0.75(0.5 to 1)
Guatemala	1,451 (746 to 2,281)	6,130 (2,791 to 10,033)	371.35(192.01 to 586.23)	460.23(209.42 to 753.55)	0.9(0.31 to 1.51)

Location	DALYs in 1990 (95%CI)	DALYs in 2021 (95%CI)	ASDR in 1990 (per 100,000 population,95%CI)	ASDR in 2021 (per 100,000 population, 95%CI)	AAPC of ASDR (95%CI)
Guinea	716 (225 to 1,404)	1,741 (534 to 3,303)	167.87(52.77 to 329.19)	260.52(80.02 to 496.78)	1.31(1.19 to 1.42)
Guinea-Bissau	94 (32 to 187)	265 (92 to 497)	196.4(66.3 to 390.08)	332.79(115.69 to 623.19)	1.36(1.17 to 1.55)
Guyana	171 (67 to 290)	494 (197 to 842)	377.68(148.33 to 643.23)	612.91(244.77 to 1045.34)	1.75(1.33 to 2.18)
Haiti	643 (274 to 1,146)	2,397 (903 to 4,345)	163.43(69.81 to 290.74)	284.78(107.88 to 517.29)	1.72(1.61 to 1.83)
Honduras	678 (325 to 1,124)	4,918 (2,192 to 8,184)	281.18(134.58 to 466.86)	638.61(284.33 to 1063.43)	2.33(1.83 to 2.84)
Hungary	25,187 (10,431 to 40,284)	33,779 (14,312 to 56,224)	1277.66(529.69 to 2044)	1285.82(547.32 to 2137.9)	-0.1(-0.2 to 0)
Iceland	293 (105 to 501)	590 (209 to 1,031)	789.27(281.21 to 1350.53)	765.28(271.04 to 1337.08)	-0.17(-0.44 to 0.1)
India	22,425 (10,676 to 37,884)	183,017 (75,213 to 307,812)	41.73(20.01 to 70.23)	123.44(51.14 to 208.09)	3.3(3.18 to 3.42)
Indonesia	7,626 (3,530 to 13,345)	53,074 (20,055 to 98,048)	66.35(31 to 116.12)	179.78(68.37 to 333.39)	2.98(2.9 to 3.06)
Iran (Islamic Republic of)	5,721 (2,499 to 9,185)	37,878 (14,525 to 61,788)	183.1(80.64 to 294.69)	416.09(160.11 to 678.57)	2.22(2.07 to 2.36)
Iraq	3,449 (1,410 to 5,840)	15,470 (6,166 to 27,124)	378.61(155.12 to 641.16)	586.75(235.24 to 1028.71)	0.93(0.76 to 1.09)
Ireland	4,240 (1,478 to 7,329)	7,363 (2,545 to 12,826)	786.04(272.4 to 1358.87)	711.8(245.1 to 1240.22)	-0.54(-0.86 to -0.22)
Israel	5,206 (1,878 to 8,838)	11,518 (4,126 to 19,835)	817.42(294.31 to 1390.28)	706.75(252.22 to 1216.85)	-0.63(-0.82 to -0.44)
Italy	80,350 (28,903 to 138,804)	138,473 (51,239 to 237,894)	676.32(242.53 to 1169.33)	719.05(267.12 to 1233)	-0.06(-0.16 to 0.05)
Jamaica	925 (325 to 1,588)	3,271 (1,261 to 5,615)	404.86(141.56 to 693.66)	844.07(326.3 to 1447.35)	2.62(1.59 to 3.66)

Location	DALYs in 1990 (95%CI)	DALYs in 2021 (95%CI)	ASDR in 1990 (per 100,000 population,95%CI)	ASDR in 2021 (per 100,000 population, 95%CI)	AAPC of ASDR (95%CI)
Japan	60,560 (30,320 to 94,764)	149,865 (65,429 to 246,936)	278.04(139.51 to 435.17)	311.88(132.79 to 514.66)	-0.03(-0.12 to 0.05)
Jordan	793 (339 to 1,306)	5,553 (2,098 to 9,484)	552.73(239.09 to 910.5)	691.96(263.97 to 1179.07)	0.41(0.01 to 0.82)
Kazakhstan	11,611 (4,739 to 19,278)	14,532 (5,733 to 24,259)	740.1(303.53 to 1229.91)	644.09(254.03 to 1073.28)	-0.48(-1.12 to 0.17)
Kenya	833 (282 to 1,542)	6,735 (2,085 to 12,115)	85.91(29.57 to 158.66)	251.41(77.69 to 453.14)	3.35(3.17 to 3.53)
Kiribati	17 (6 to 30)	52 (19 to 92)	378.45(139.85 to 673.34)	617.83(216.92 to 1102.89)	1.29(1.22 to 1.36)
Kuwait	263 (108 to 439)	1,717 (718 to 2,817)	452.51(187.01 to 757.54)	627.4(262.03 to 1032.34)	0.9(-0.5 to 2.31)
Kyrgyzstan	2,030 (786 to 3,403)	3,262 (1,282 to 5,395)	537.73(208.48 to 903.06)	551.97(217.71 to 916.13)	-0.08(-0.33 to 0.17)
Lao People's Democratic Republic	283 (127 to 531)	1,097 (416 to 2,048)	111.85(50.52 to 208.74)	200.05(76.07 to 373.91)	1.8(1.78 to 1.83)
Latvia	3,837 (1,542 to 6,338)	6,085 (2,384 to 10,268)	821.34(330.3 to 1356.16)	1156.87(452.74 to 1951.09)	0.99(0.62 to 1.36)
Lebanon	1,549 (559 to 2,828)	5,932 (1,988 to 10,069)	594.96(215.41 to 1080.72)	792.08(265.14 to 1345.27)	0.73(0.47 to 0.98)
Lesotho	334 (113 to 611)	1,194 (419 to 2,256)	327.99(110.88 to 602.82)	917.8(319.34 to 1734.52)	3.7(3.49 to 3.92)
Liberia	436 (149 to 818)	1,064 (369 to 1,971)	296.12(100.78 to 555.3)	474.54(164.55 to 882.15)	1.27(0.99 to 1.54)
Libya	1,187 (503 to 2,005)	6,323 (2,523 to 10,848)	545.37(231.29 to 922.93)	1149.47(459.23 to 1972.58)	2.41(2.22 to 2.6)
Lithuania	4,165 (1,740 to 6,947)	8,496 (3,342 to 14,422)	720.04(301.13 to 1198.06)	1123.99(441.05 to 1908.87)	1.28(0.79 to 1.78)
Luxembourg	634 (240 to 1,066)	1,038 (396 to 1,784)	892.52(336.11 to 1501.9)	762.22(291.02 to 1310.98)	-0.82(-0.96 to -0.68)

Location	DALYs in 1990 (95%CI)	DALYs in 2021 (95%CI)	ASDR in 1990 (per 100,000 population,95%CI)	ASDR in 2021 (per 100,000 population, 95%CI)	AAPC of ASDR (95%CI)
Madagascar	581 (201 to 1,150)	2,242 (692 to 4,226)	96.36(33.34 to 190.21)	185.91(57.91 to 354.9)	1.96(1.83 to 2.09)
Malawi	358 (126 to 639)	1,567 (500 to 2,979)	79.56(28.32 to 141.31)	186.97(59.68 to 353.82)	2.8(2.62 to 2.98)
Malaysia	2,931 (1,194 to 4,963)	17,290 (6,615 to 28,966)	271.56(110.81 to 460.06)	482.65(184.81 to 808.73)	1.74(1.59 to 1.89)
Maldives	12 (5 to 21)	55 (19 to 99)	102.65(44.24 to 181.27)	145.34(51.53 to 264.25)	0.44(0.29 to 0.6)
Mali	886 (292 to 1,720)	2,610 (803 to 5,097)	179.26(59.21 to 347.71)	249.19(76.87 to 487.22)	1.18(1.11 to 1.24)
Malta	347 (115 to 597)	932 (340 to 1,633)	629.52(208.53 to 1083.42)	697.87(254.89 to 1223.51)	0.17(0 to 0.34)
Marshall Islands	9 (4 to 16)	28 (11 to 50)	477.61(197.2 to 826.98)	698.81(264.81 to 1223.15)	1.35(1.3 to 1.4)
Mauritania	605 (189 to 1,266)	1,546 (509 to 2,830)	516.85(159.57 to 1089.4)	615.85(203.48 to 1125.25)	0.19(0.06 to 0.31)
Mauritius	244 (116 to 393)	1,187 (420 to 2,043)	263.15(124.31 to 424.55)	474.92(168.1 to 815.92)	2.06(1.62 to 2.5)
Mexico	19,692 (8,653 to 32,233)	99,898 (41,239 to 167,381)	399.91(176.27 to 654.84)	640.34(264.36 to 1072.85)	1.67(1.57 to 1.78)
Micronesia (Federated States of)	33 (13 to 60)	73 (26 to 127)	533.12(211.55 to 961.17)	783.78(274.71 to 1370.39)	1.09(1.07 to 1.12)
Monaco	84 (27 to 154)	160 (55 to 288)	889.19(279.06 to 1631.62)	1221.69(415.09 to 2203.37)	1(0.94 to 1.07)
Mongolia	1,196 (493 to 2,168)	3,729 (1,519 to 6,569)	946.76(390.82 to 1718.68)	1476.78(599.59 to 2607.59)	1.12(0.68 to 1.56)
Montenegro	586 (220 to 984)	1,556 (609 to 2,609)	758.15(284.38 to 1273.31)	1179.78(460.88 to 1983.49)	1.27(1.06 to 1.49)
Morocco	2,290 (826 to 3,996)	11,854 (3,772 to 21,021)	132.78(48.11 to 231.64)	277.65(89.61 to 492.25)	2.23(2.13 to 2.33)
Mozambique	864 (322 to 1,627)	3,888 (1,270 to 8,030)	124.42(46.89 to 234.16)	309.13(100.7 to 637.48)	3.26(3.15 to 3.38)
Myanmar	3,307 (1,388 to 5,904)	10,859 (4,123 to 19,501)	111.29(47.4 to 198.44)	174.57(66.62 to 315.16)	0.98(0.92 to 1.03)
Namibia	143 (46 to 269)	748 (202 to 1,402)	183.81(59.08 to 346.29)	481.07(131.11 to 897.57)	2.93(2.71 to 3.15)
Nauru	4 (2 to 7)	7 (3 to 13)	704.06(273.39 to 1306.91)	1042.98(379.39 to 1875.57)	0.99(0.93 to 1.06)

Location	DALYs in 1990 (95%CI)	DALYs in 2021 (95%CI)	ASDR in 1990 (per 100,000 population,95%CI)	ASDR in 2021 (per 100,000 population, 95%CI)	AAPC of ASDR (95%CI)
Nepal	447 (208 to 832)	2,656 (1,141 to 4,774)	39.21(18.44 to 72.48)	89.2(38.62 to 160.05)	2.63(2.5 to 2.75)
Netherlands	20,055 (6,671 to 34,910)	36,266 (12,835 to 63,011)	766.95(254.84 to 1335.99)	768.56(271.38 to 1334.76)	-0.13(-0.28 to 0.02)
New Zealand	4,860 (1,722 to 8,287)	9,744 (3,692 to 16,410)	937.61(331.62 to 1599.02)	869.54(329.72 to 1462.55)	-0.31(-0.44 to -0.19)
Nicaragua	501 (232 to 801)	2,631 (1,098 to 4,433)	283.93(131.75 to 454.32)	451.33(188.78 to 760.74)	1.22(0.74 to 1.71)
Niger	371 (118 to 751)	1,379 (441 to 2,650)	113.17(36.09 to 229.35)	141.88(45.62 to 273.81)	0.35(0.2 to 0.5)
Nigeria	7,732 (1,950 to 14,504)	35,309 (7,619 to 66,082)	148.71(37.32 to 279.5)	356.38(78.79 to 662.97)	2.94(2.82 to 3.05)
Niue	1 (1 to 2)	2 (1 to 4)	493.19(185.33 to 883.01)	773.08(277.92 to 1334.17)	1.34(1.21 to 1.47)
North Macedonia	1,844 (768 to 3,040)	4,768 (1,954 to 8,175)	796.79(334.08 to 1313.19)	1099.87(449.91 to 1885.51)	0.74(0.53 to 0.95)
Northern Mariana Islands	9 (4 to 17)	63 (26 to 101)	632.91(244.01 to 1112.88)	1018.49(411.42 to 1662.53)	1.32(1.08 to 1.56)
Norway	6,229 (2,165 to 10,731)	8,269 (2,965 to 14,207)	680.51(236.41 to 1171.41)	618.46(220.53 to 1062.97)	-0.5(-0.76 to -0.23)
Oman	133 (57 to 232)	764 (288 to 1,304)	180.57(77.63 to 314.33)	388.85(148.13 to 664.99)	2.09(1.86 to 2.33)
Pakistan	9,428 (4,487 to 16,029)	51,849 (20,799 to 89,476)	140.13(66.78 to 238.33)	374.02(150.49 to 644.88)	3.03(2.9 to 3.17)
Palau	8 (2 to 14)	22 (6 to 41)	642.62(172.06 to 1187.87)	820.21(216.01 to 1538.8)	0.6(0.43 to 0.77)
Palestine	820 (339 to 1,416)	2,864 (1,105 to 4,678)	789.66(328.3 to 1362.11)	1043.61(405.09 to 1703.63)	0.6(0.48 to 0.72)
Panama	870 (358 to 1,437)	4,105 (1,679 to 6,920)	489.49(201.22 to 809.12)	750.16(306.81 to 1264.34)	1.76(1.61 to 1.9)
Papua New Guinea	298 (114 to 558)	976 (345 to 1,800)	130.04(51.12 to 242.08)	165.18(59.82 to 303.94)	0.83(0.73 to 0.93)
Paraguay	878 (393 to 1,484)	4,520 (1,854 to 7,954)	330.78(148.2 to 559.83)	636.63(261.18 to 1120)	2.13(1.99 to 2.26)

Location	DALYs in 1990 (95%CI)	DALYs in 2021 (95%CI)	ASDR in 1990 (per 100,000 population,95%CI)	ASDR in 2021 (per 100,000 population, 95%CI)	AAPC of ASDR (95%CI)
Peru	5,229 (2,417 to 8,760)	22,316 (10,040 to 38,092)	373.39(173.15 to 626.1)	549.14(247.08 to 937.3)	1.14(0.31 to 1.98)
Philippines	4,369 (1,699 to 7,418)	29,109 (10,476 to 50,499)	130.3(50.62 to 221.71)	289.87(104.61 to 504.33)	2.17(2.05 to 2.29)
Poland	54,106 (23,380 to 87,984)	118,907 (48,953 to 197,802)	943.38(407.62 to 1536.15)	1204.4(495.54 to 2003.87)	0.39(0.31 to 0.47)
Portugal	10,888 (4,254 to 18,662)	22,693 (8,629 to 39,120)	589.33(229.55 to 1011.59)	698.55(265.74 to 1204.26)	0.45(0.27 to 0.62)
Puerto Rico	2,694 (993 to 4,568)	7,142 (2,726 to 12,096)	582.46(214.66 to 987.38)	762.75(289.07 to 1288.96)	0.84(0.64 to 1.05)
Qatar	75 (30 to 132)	924 (341 to 1,593)	906.9(364.8 to 1580.74)	1356.46(512.21 to 2318.08)	0.98(0.52 to 1.45)
Republic of Korea	8,699 (4,728 to 14,204)	37,987 (16,448 to 65,652)	260.95(141.67 to 426.07)	306.51(133.03 to 529.42)	-0.02(-0.17 to 0.13)
Republic of Moldova	4,255 (1,733 to 6,977)	8,007 (3,013 to 13,163)	733.18(299.18 to 1203.44)	985.33(371.56 to 1620.88)	0.72(-0.04 to 1.5)
Romania	18,572 (7,351 to 30,881)	48,836 (17,928 to 83,661)	504.06(200.2 to 837.4)	978.6(358.62 to 1678.23)	1.88(1.48 to 2.28)
Russian Federation	178,033 (74,898 to 288,457)	364,481 (144,955 to 590,065)	744.06(313.45 to 1202.86)	1106.6(440.76 to 1790.43)	0.99(0.22 to 1.76)
Rwanda	502 (186 to 987)	1,712 (507 to 3,375)	146.13(54 to 285.82)	230.73(68.96 to 455.86)	1.1(0.95 to 1.25)
Saint Kitts and Nevis	36 (12 to 63)	84 (32 to 141)	680.41(227.23 to 1183.87)	957.03(365.79 to 1624.01)	0.7(0.35 to 1.06)
Saint Lucia	48 (17 to 84)	160 (55 to 281)	430.11(147.81 to 750.57)	531.55(182.6 to 934.27)	0.58(0.47 to 0.69)
Saint Vincent and the Grenadines	34 (12 to 60)	94 (34 to 163)	371.51(133.14 to 645.93)	507.23(183.03 to 879.83)	1.3(0.79 to 1.8)

Location	DALYs in 1990 (95%CI)	DALYs in 2021 (95%CI)	ASDR in 1990 (per 100,000 population,95%CI)	ASDR in 2021 (per 100,000 population, 95%CI)	AAPC of ASDR (95%CI)
Samoa	63 (27 to 106)	134 (56 to 224)	593.5(253.18 to 997.08)	765.45(320.52 to 1289.23)	0.83(0.75 to 0.91)
San Marino	35 (14 to 61)	49 (18 to 91)	756.15(291 to 1300.35)	507.1(182.3 to 945.48)	-1.32(-1.6 to -1.04)
Sao Tome and Principe	17 (7 to 29)	50 (19 to 86)	203.48(82.06 to 351.55)	399.34(152.74 to 696.79)	2.2(2.05 to 2.36)
Saudi Arabia	2,035 (858 to 3,445)	12,404 (5,102 to 20,498)	325.42(137.86 to 550.13)	699.81(291.03 to 1156.3)	2.27(2.18 to 2.37)
Senegal	619 (214 to 1,115)	2,505 (853 to 4,556)	159.03(55.1 to 287.38)	274.87(93.92 to 500.25)	1.37(1.18 to 1.57)
Serbia	12,393 (4,756 to 21,143)	28,009 (10,853 to 46,669)	872.45(338.77 to 1495.44)	1237.08(478.46 to 2061.45)	0.99(0.62 to 1.37)
Seychelles	30 (12 to 51)	98 (36 to 169)	417.92(165.03 to 708.27)	680.7(252.49 to 1175.73)	1.32(1.19 to 1.45)
Sierra Leone	327 (98 to 683)	923 (298 to 1,786)	131.07(39.26 to 273.6)	215.88(69.26 to 418.37)	1.55(1.41 to 1.68)
Singapore	604 (248 to 1,016)	3,938 (1,461 to 6,774)	229.98(94.86 to 386.82)	352.39(130.79 to 607.64)	0.88(0.53 to 1.23)
Slovakia	10,370 (4,534 to 16,853)	18,179 (7,617 to 30,139)	1301.39(568.69 to 2115.14)	1402.76(587.82 to 2328.27)	-0.1(-0.41 to 0.22)
Slovenia	3,149 (1,255 to 5,201)	6,227 (2,478 to 10,489)	997.65(398.09 to 1647.99)	1047.56(418.03 to 1766.2)	-0.27(-0.76 to 0.21)
Solomon Islands	36 (14 to 68)	133 (48 to 241)	218.58(84.78 to 408.68)	347.66(125.71 to 628.83)	1.5(1.14 to 1.86)
Somalia	270 (106 to 515)	1,310 (488 to 2,487)	111.46(44.16 to 211.17)	188.18(69.93 to 357.78)	1.48(1.42 to 1.54)
South Africa	9,122 (2,996 to 15,685)	43,615 (14,680 to 73,271)	377.58(123.87 to 649.8)	788.45(265.38 to 1326.49)	2.05(1.55 to 2.54)
South Sudan	229 (95 to 435)	509 (187 to 973)	73.27(30.15 to 139.31)	124.2(45.99 to 236.62)	1.75(1.65 to 1.86)
Spain	54,600 (21,458 to 92,045)	108,273 (41,012 to 182,772)	747.06(293.2 to 1259.8)	861.86(329.09 to 1447.72)	0.25(0.06 to 0.44)
Sri Lanka	1,606 (711 to 2,726)	6,193 (2,241 to 11,861)	126.91(56.62 to 215.03)	170.75(61.78 to 327.28)	0.72(0.39 to 1.05)

Location	DALYs in 1990 (95%CI)	DALYs in 2021 (95%CI)	ASDR in 1990 (per 100,000 population,95%CI)	ASDR in 2021 (per 100,000 population, 95%CI)	AAPC of ASDR (95%CI)
Sudan	2,122 (964 to 3,590)	7,787 (3,193 to 13,266)	192.03(87.14 to 325.66)	365.65(150.15 to 621.45)	1.84(1.73 to 1.95)
Suriname	79 (28 to 139)	333 (113 to 595)	258.52(92.1 to 456.13)	423.36(143.24 to 756.22)	1.7(1.26 to 2.14)
Sweden	14,732 (5,545 to 25,152)	18,555 (6,929 to 32,167)	737.56(276.15 to 1260.76)	640.49(237.39 to 1109)	-0.73(-0.84 to -0.62)
Switzerland	7,477 (2,743 to 12,665)	11,960 (4,169 to 20,895)	554.37(202.38 to 941.91)	500.43(173.78 to 873.66)	-0.66(-1.09 to -0.24)
Syrian Arab Republic	2,072 (870 to 3,490)	9,492 (3,695 to 16,148)	344.16(145.9 to 579.98)	602.33(237.79 to 1021.75)	1.3(1.11 to 1.49)
Taiwan (Province of China)	4,280 (1,805 to 6,940)	31,113 (11,722 to 53,118)	209.61(88.73 to 340.48)	549.83(207.68 to 937.36)	2.89(2.76 to 3.01)
Tajikistan	1,185 (470 to 2,008)	2,250 (897 to 3,931)	364.49(143.84 to 618.61)	332.4(132.97 to 578.57)	-0.42(-0.73 to -0.11)
Thailand	7,375 (3,658 to 12,569)	60,646 (25,623 to 105,284)	180.14(89.93 to 306.96)	424.29(179.25 to 737)	2.58(2.39 to 2.77)
Timor-Leste	11 (5 to 19)	78 (34 to 142)	38.53(18.34 to 67.51)	69.75(30.24 to 127.83)	2.09(1.95 to 2.22)
Togo	193 (62 to 366)	1,223 (367 to 2,296)	139.28(45.15 to 264.43)	296.25(88.78 to 556.13)	2.22(2.07 to 2.37)
Tokelau	1 (0 to 1)	1 (0 to 2)	429.96(164.92 to 750.89)	651.61(237.82 to 1137.76)	1.22(1.14 to 1.3)
Tonga	62 (21 to 108)	121 (43 to 215)	875.42(298.26 to 1545.81)	1247.76(446.07 to 2216.68)	0.92(0.81 to 1.02)
Trinidad and Tobago	608 (228 to 1,015)	2,053 (788 to 3,492)	587.66(220.04 to 981.27)	785.41(300.69 to 1335.93)	1.11(0.86 to 1.36)
Tunisia	1,454 (599 to 2,517)	7,367 (2,812 to 13,502)	236.74(98.7 to 409.2)	437.3(167.85 to 799.32)	1.93(1.81 to 2.06)

Location	DALYs in 1990 (95%CI)	DALYs in 2021 (95%CI)	ASDR in 1990 (per 100,000 population,95%CI)	ASDR in 2021 (per 100,000 population, 95%CI)	AAPC of ASDR (95%CI)
Turkey	22,801 (10,183 to 37,212)	93,109 (35,474 to 156,559)	562.23(252.68 to 916.8)	790.46(301.76 to 1331.15)	0.57(0.35 to 0.8)
Turkmenistan	918 (401 to 1,509)	2,141 (734 to 3,771)	389.56(170.5 to 639.49)	425.11(146.5 to 748.76)	0.5(0.1 to 0.91)
Tuvalu	4 (2 to 7)	9 (3 to 15)	420.86(169.09 to 726.94)	650.71(237.88 to 1133.57)	1.21(1.18 to 1.24)
Uganda	1,058 (363 to 1,996)	5,112 (1,564 to 9,420)	137.26(47.19 to 259.01)	308.85(95.24 to 570.7)	2.73(2.5 to 2.97)
Ukraine	72,960 (28,870 to 118,229)	93,421 (36,369 to 159,637)	749.59(297.22 to 1214.34)	879.57(342.11 to 1504.31)	0.56(-0.18 to 1.3)
United Arab Emirates	328 (135 to 582)	4,030 (1,573 to 6,738)	915.48(379.23 to 1616.61)	1613.44(644.38 to 2657.44)	1.25(0.17 to 2.34)
United Kingdom	111,278 (36,440 to 192,444)	158,468 (58,096 to 268,988)	925.93(301.9 to 1602.48)	921.27(338.72 to 1559.55)	-0.08(-0.24 to 0.08)
United Republic of Tanzania	2,399 (837 to 4,485)	10,927 (3,530 to 19,895)	184.61(64.05 to 343.69)	385.81(123.78 to 705.14)	2.19(2.11 to 2.26)
United States Virgin Islands	81 (30 to 137)	166 (57 to 291)	842.75(311.41 to 1428.36)	663.98(228.21 to 1163.56)	-0.42(-0.72 to -0.12)
United States of America	395,545 (141,941 to 667,080)	791,098 (307,011 to 1,296,472)	937.13(335.73 to 1579.86)	1001.21(388.56 to 1639.75)	0.18(0.06 to 0.3)
Uruguay	4,922 (1,793 to 8,401)	8,930 (3,334 to 15,272)	949.33(345.52 to 1620.21)	1244.28(465.01 to 2122.3)	0.76(0.66 to 0.86)
Uzbekistan	4,525 (1,731 to 7,496)	12,446 (4,823 to 21,668)	327.32(125.74 to 544.11)	382.59(148.4 to 664.46)	0.53(0.11 to 0.95)
Vanuatu	16 (6 to 28)	74 (28 to 128)	218.75(88.44 to 389.63)	366.67(138.06 to 639.68)	1.59(1.33 to 1.86)
Venezuela (Bolivarian Republic of)	6,253 (2,760 to 10,147)	26,749 (10,119 to 46,707)	553.02(244.59 to 898.63)	699.95(265.87 to 1221.33)	0.8(0.27 to 1.34)

Location	DALYs in 1990 (95%CI)	DALYs in 2021 (95%CI)	ASDR in 1990 (per 100,000 population,95%CI)	ASDR in 2021 (per 100,000 population, 95%CI)	AAPC of ASDR (95%CI)
Viet Nam	2,183 (1,004 to 3,746)	14,482 (5,671 to 26,816)	42.89(19.83 to 73.6)	114.83(44.87 to 213.45)	3.34(3.2 to 3.48)
Yemen	666 (319 to 1,170)	3,856 (1,591 to 6,594)	116.16(55.41 to 204.47)	243.92(100.92 to 418.29)	2.04(1.81 to 2.26)
Zambia	474 (181 to 892)	3,331 (997 to 6,708)	147.99(56.51 to 277.35)	445.36(133.15 to 893.57)	3.33(3.16 to 3.51)
Zimbabwe	1,287 (442 to 2,384)	7,196 (2,456 to 12,863)	264.31(90.95 to 489.87)	890.69(303.98 to 1599.17)	3.97(3.39 to 4.55)

Table S8. ASDR for specific cancer types attributable to high BMI among older people globally and regionally in 2021, by sex.

Cause	Sex	Location	DALYs (95%CI)	ASDR (per 100,000 population, 95%CI)	AAPC (1990 – 2021, 95%CI)
Colon and rectum cancer	Male	Global	776,838 (333,602 to 1,238,879)	157.8(67.68 to 251.99)	0.54(0.41 to 0.67)
Colon and rectum cancer	Male	High SDI	307,579 (130,892 to 488,362)	245.28(104.47 to 389.33)	-0.28(-0.43 to -0.12)
Colon and rectum cancer	Male	High-middle SDI	264,516 (113,618 to 429,283)	235.74(101.18 to 383.04)	0.92(0.69 to 1.14)
Colon and rectum cancer	Male	Middle SDI	156,009 (65,366 to 254,885)	101.65(42.44 to 166.34)	2.67(2.54 to 2.8)
Colon and rectum cancer	Male	Low-middle SDI	38,050 (15,478 to 61,013)	46.49(18.84 to 74.63)	3.12(3.02 to 3.22)
Colon and rectum cancer	Male	Low SDI	9,300 (3,389 to 15,208)	33.98(12.31 to 55.64)	1.99(1.92 to 2.05)
Colon and rectum cancer	Male	High-income Asia Pacific	33,989 (12,761 to 55,855)	126.01(47.47 to 207.24)	0.53(0.34 to 0.73)
Colon and rectum cancer	Male	High-income North America	108,013 (48,252 to 168,097)	268.23(119.74 to 417.72)	-0.22(-0.49 to 0.05)
Colon and rectum cancer	Male	Western Europe	152,417 (64,721 to 249,018)	276.48(117.68 to 451.13)	-0.49(-0.63 to -0.35)
Colon and rectum cancer	Male	Australasia	9,684 (4,240 to 15,643)	289.85(127.19 to 468.11)	-0.6(-0.8 to -0.4)
Colon and rectum cancer	Male	Andean Latin America	4,160 (1,723 to 7,213)	122.46(50.65 to 212.48)	1.84(1.31 to 2.37)
Colon and rectum cancer	Male	Tropical Latin America	24,804 (10,442 to 40,183)	174.34(73.28 to 282.76)	2.55(2.23 to 2.87)
Colon and rectum cancer	Male	Central Latin America	21,201 (8,927 to 34,499)	150.44(63.23 to 245.1)	3.05(2.98 to 3.12)
Colon and rectum cancer	Male	Southern Latin America	17,524 (7,531 to 29,112)	357.98(153.7 to 595.17)	0.86(0.64 to 1.08)
Colon and rectum cancer	Male	Caribbean	5,492 (2,280 to 9,155)	176.73(73.34 to 294.57)	1.83(1.63 to 2.03)
Colon and rectum cancer	Male	Central Europe	66,956 (29,162 to 108,452)	525.27(228.55 to 851.45)	0.93(0.83 to 1.03)
Colon and rectum cancer	Male	Eastern Europe	63,172 (26,331 to 102,769)	364.54(152.16 to 593.03)	1.18(0.56 to 1.81)
Colon and rectum cancer	Male	Central Asia	5,227 (2,195 to 8,440)	123.54(51.64 to 200.17)	-0.05(-0.21 to 0.12)
Colon and rectum cancer	Male	North Africa and Middle East	40,669 (17,306 to 65,225)	158.18(67.13 to 254.6)	1.9(1.82 to 1.98)
Colon and rectum cancer	Male	South Asia	18,416 (6,811 to 30,794)	20.82(7.68 to 34.83)	3.26(3.2 to 3.33)
Colon and rectum cancer	Male	Southeast Asia	24,487 (9,089 to 41,054)	66.34(24.33 to 111.42)	3.3(3.24 to 3.36)

Cause	Sex	Location	DALYs (95%CI)	ASDR (per 100,000 population, 95%CI)	AAPC (1990 – 2021, 95%CI)
Colon and rectum cancer	Male	East Asia	164,152 (63,919 to 285,837)	127.83(49.68 to 222.06)	2.87(2.72 to 3.01)
Colon and rectum cancer	Male	Oceania	244 (103 to 404)	58.59(24.58 to 97.54)	0.48(0.32 to 0.63)
Colon and rectum cancer	Male	Western Sub-Saharan Africa	5,292 (2,017 to 8,912)	54.24(20.65 to 91.37)	2.52(2.42 to 2.62)
Colon and rectum cancer	Male	Eastern Sub-Saharan Africa	4,710 (1,643 to 8,008)	55.51(19.06 to 94.63)	1.94(1.82 to 2.05)
Colon and rectum cancer	Male	Central Sub-Saharan Africa	1,604 (574 to 2,920)	63.39(22.32 to 117.67)	2.71(2.63 to 2.8)
Colon and rectum cancer	Male	Southern Sub-Saharan Africa	4,624 (1,897 to 7,502)	173.65(71.15 to 282.27)	2.51(1.99 to 3.04)
Colon and rectum cancer	Male	Four World Regions	774,101 (332,364 to 1,234,436)	157.54(67.55 to 251.57)	0.54(0.41 to 0.67)
Colon and rectum cancer	Male	Asia	268,572 (107,821 to 441,549)	92.25(37.02 to 151.58)	2.26(2.15 to 2.36)
Colon and rectum cancer	Male	America	179,678 (80,189 to 281,923)	226.52(100.94 to 355.75)	0.25(0.05 to 0.44)
Colon and rectum cancer	Male	Europe	297,045 (127,090 to 479,832)	331.25(141.76 to 535)	0.19(0.06 to 0.32)
Colon and rectum cancer	Male	Africa	28,805 (11,656 to 46,624)	85.09(34.34 to 138.04)	2.52(2.35 to 2.69)
Gallbladder and biliary tract cancer	Male	Global	120,077 (78,080 to 170,633)	24.5(15.94 to 34.83)	0.31(0.11 to 0.5)
Gallbladder and biliary tract cancer	Male	High SDI	42,831 (28,562 to 59,949)	33.94(22.63 to 47.5)	-0.53(-0.76 to -0.31)
Gallbladder and biliary tract cancer	Male	High-middle SDI	34,497 (21,516 to 50,601)	30.83(19.27 to 45.21)	0.27(0.18 to 0.36)
Gallbladder and biliary tract cancer	Male	Middle SDI	30,750 (18,589 to 44,762)	20.07(12.18 to 29.25)	1.28(1.14 to 1.43)
Gallbladder and biliary tract cancer	Male	Low-middle SDI	10,306 (6,395 to 14,933)	12.78(7.93 to 18.57)	2.17(2.05 to 2.29)
Gallbladder and biliary tract cancer	Male	Low SDI	1,551 (830 to 2,309)	5.67(3.04 to 8.44)	2.06(1.92 to 2.2)

Cause	Sex	Location	DALYs (95%CI)	ASDR (per 100,000 population, 95%CI)	AAPC (1990 – 2021, 95%CI)
Gallbladder and biliary tract cancer	Male	High-income Asia Pacific	16,236 (10,615 to 22,987)	58.01(37.84 to 82.29)	-0.89(-1.05 to -0.73)
Gallbladder and biliary tract cancer	Male	High-income North America	7,914 (5,238 to 10,827)	19.63(12.99 to 26.89)	-0.22(-0.61 to 0.18)
Gallbladder and biliary tract cancer	Male	Western Europe	16,333 (10,856 to 22,939)	29.49(19.64 to 41.36)	-0.87(-1.02 to -0.71)
Gallbladder and biliary tract cancer	Male	Australasia	689 (445 to 983)	20.55(13.28 to 29.29)	-0.24(-1.76 to 1.3)
Gallbladder and biliary tract cancer	Male	Andean Latin America	1,591 (947 to 2,508)	46.67(27.77 to 73.52)	0.42(-0.06 to 0.9)
Gallbladder and biliary tract cancer	Male	Tropical Latin America	4,831 (3,249 to 6,743)	34.05(22.87 to 47.59)	0.92(0.47 to 1.38)
Gallbladder and biliary tract cancer	Male	Central Latin America	4,412 (2,918 to 6,277)	31.48(20.84 to 44.8)	-0.57(-1.11 to -0.02)
Gallbladder and biliary tract cancer	Male	Southern Latin America	3,873 (2,529 to 5,550)	79.01(51.57 to 113.27)	-0.27(-0.57 to 0.03)
Gallbladder and biliary tract cancer	Male	Caribbean	433 (285 to 621)	13.92(9.16 to 19.96)	0(-0.48 to 0.48)
Gallbladder and biliary tract cancer	Male	Central Europe	6,587 (4,403 to 9,339)	51.73(34.56 to 73.31)	-0.28(-0.77 to 0.21)
Gallbladder and biliary tract cancer	Male	Eastern Europe	4,211 (2,697 to 5,942)	24.3(15.59 to 34.3)	0.54(-1.18 to 2.29)
Gallbladder and biliary tract cancer	Male	Central Asia	583 (387 to 851)	13.84(9.23 to 20.11)	-0.24(-1.01 to 0.54)

Cause	Sex	Location	DALYs (95%CI)	ASDR (per 100,000 population, 95%CI)	AAPC (1990 – 2021, 95%CI)
Gallbladder and biliary tract cancer	Male	North Africa and Middle East	5,798 (3,204 to 8,862)	22.68(12.57 to 34.67)	1.44(1.37 to 1.51)
Gallbladder and biliary tract cancer	Male	South Asia	10,341 (5,436 to 15,545)	11.96(6.28 to 18.01)	2.86(2.68 to 3.05)
Gallbladder and biliary tract cancer	Male	Southeast Asia	6,639 (2,976 to 10,625)	18.49(8.29 to 29.65)	2.21(2.06 to 2.35)
Gallbladder and biliary tract cancer	Male	East Asia	28,636 (14,955 to 46,695)	22.32(11.7 to 36.18)	1.38(1.19 to 1.56)
Gallbladder and biliary tract cancer	Male	Oceania	34 (17 to 53)	7.81(4.01 to 12.2)	0.53(0.44 to 0.63)
Gallbladder and biliary tract cancer	Male	Western Sub-Saharan Africa	35 (20 to 63)	0.34(0.2 to 0.62)	1.37(1.27 to 1.47)
Gallbladder and biliary tract cancer	Male	Eastern Sub-Saharan Africa	473 (238 to 718)	5.5(2.78 to 8.32)	1.21(1.15 to 1.27)
Gallbladder and biliary tract cancer	Male	Central Sub-Saharan Africa	75 (33 to 131)	3.03(1.31 to 5.33)	2.21(2.14 to 2.28)
Gallbladder and biliary tract cancer	Male	Southern Sub-Saharan Africa	352 (181 to 529)	13(6.67 to 19.61)	1.92(1.63 to 2.2)
Gallbladder and biliary tract cancer	Male	Four World Regions	119,860 (77,915 to 170,323)	24.51(15.93 to 34.83)	0.31(0.11 to 0.51)
Gallbladder and biliary tract cancer	Male	Asia	64,788 (37,120 to 94,312)	22.77(13.18 to 33.1)	1.04(0.96 to 1.12)
Gallbladder and biliary tract cancer	Male	America	22,965 (15,520 to 31,601)	28.94(19.54 to 39.86)	0.05(-0.15 to 0.25)

Cause	Sex	Location	DALYs (95%CI)	ASDR (per 100,000 population, 95%CI)	AAPC (1990 – 2021, 95%CI)
Gallbladder and biliary tract cancer	Male	Europe	28,741 (19,227 to 40,269)	32.03(21.43 to 44.86)	-0.46(-0.75 to -0.16)
Gallbladder and biliary tract cancer	Male	Africa	3,366 (1,794 to 5,196)	10.09(5.38 to 15.57)	1.98(1.83 to 2.12)
Liver cancer	Male	Global	387,234 (157,408 to 655,880)	75.66(30.74 to 128.05)	2.53(2.4 to 2.65)
Liver cancer	Male	High SDI	150,331 (62,051 to 251,643)	121.2(50.05 to 202.83)	2.31(2.19 to 2.42)
Liver cancer	Male	High-middle SDI	92,112 (35,165 to 163,896)	79.16(30.25 to 140.67)	2.24(1.86 to 2.62)
Liver cancer	Male	Middle SDI	88,316 (35,028 to 150,270)	55.25(21.93 to 93.97)	3.24(3.03 to 3.45)
Liver cancer	Male	Low-middle SDI	48,929 (19,222 to 85,791)	57.41(22.55 to 100.52)	3.41(2.98 to 3.85)
Liver cancer	Male	Low SDI	7,189 (2,650 to 12,841)	24.84(9.18 to 44.26)	2.08(1.96 to 2.2)
Liver cancer	Male	High-income Asia Pacific	18,796 (7,281 to 34,403)	72.49(28.03 to 132.96)	-0.38(-0.59 to -0.16)
Liver cancer	Male	High-income North America	66,983 (28,214 to 109,198)	164.28(69.19 to 267.89)	3.92(3.74 to 4.1)
Liver cancer	Male	Western Europe	64,348 (25,121 to 116,330)	120.17(46.99 to 217.2)	1.94(1.56 to 2.32)
Liver cancer	Male	Australasia	5,070 (1,972 to 9,117)	155.07(60.54 to 278.68)	4.72(4.44 to 5.01)
Liver cancer	Male	Andean Latin America	1,482 (569 to 2,734)	43.02(16.49 to 79.39)	1.95(1.28 to 2.62)
Liver cancer	Male	Tropical Latin America	6,671 (2,634 to 11,761)	45.83(18.07 to 80.87)	2.89(2.44 to 3.35)
Liver cancer	Male	Central Latin America	9,512 (3,831 to 16,690)	67.03(26.96 to 117.66)	2.26(1.41 to 3.11)
Liver cancer	Male	Southern Latin America	2,915 (1,142 to 5,225)	58.61(22.95 to 105.09)	3.98(3.37 to 4.6)
Liver cancer	Male	Caribbean	1,106 (439 to 1,950)	35.3(14 to 62.26)	2.11(1.74 to 2.48)
Liver cancer	Male	Central Europe	12,717 (5,131 to 22,482)	98.22(39.62 to 173.88)	0.73(0.34 to 1.13)
Liver cancer	Male	Eastern Europe	12,465 (4,958 to 22,268)	68.3(27.25 to 121.96)	2.41(1.85 to 2.98)
Liver cancer	Male	Central Asia	6,461 (2,606 to 11,770)	142.24(57.33 to 258.97)	0.16(-0.21 to 0.53)

Cause	Sex	Location	DALYs (95%CI)	ASDR (per 100,000 population, 95%CI)	AAPC (1990 – 2021, 95%CI)
Liver cancer	Male	North Africa and Middle East	47,231 (18,566 to 84,229)	172.52(67.97 to 307.56)	2.86(2.65 to 3.07)
Liver cancer	Male	South Asia	14,556 (5,421 to 26,284)	15.93(5.94 to 28.85)	5.24(5.01 to 5.46)
Liver cancer	Male	Southeast Asia	13,583 (4,872 to 25,808)	34.74(12.53 to 66.07)	2.83(2.65 to 3)
Liver cancer	Male	East Asia	88,682 (33,435 to 164,426)	65.64(24.81 to 121.6)	3.57(3.1 to 4.03)
Liver cancer	Male	Oceania	257 (99 to 501)	55.58(21.44 to 107.76)	0.58(0.3 to 0.86)
Liver cancer	Male	Western Sub-Saharan Africa	6,718 (2,635 to 11,975)	65.96(25.92 to 116.93)	1.72(1.56 to 1.89)
Liver cancer	Male	Eastern Sub-Saharan Africa	2,261 (764 to 4,896)	24.39(8.19 to 52.47)	3.35(3.24 to 3.46)
Liver cancer	Male	Central Sub-Saharan Africa	1,321 (366 to 3,503)	48.59(13.2 to 130.75)	2.62(2.34 to 2.9)
Liver cancer	Male	Southern Sub-Saharan Africa	4,100 (1,598 to 7,321)	147.52(56.85 to 264.56)	4.02(3.64 to 4.4)
Liver cancer	Male	Four World Regions	386,417 (157,049 to 654,433)	75.63(30.72 to 127.99)	2.53(2.4 to 2.65)
Liver cancer	Male	Asia	156,240 (60,657 to 270,375)	51.27(19.94 to 88.63)	2.69(2.37 to 3.02)
Liver cancer	Male	America	88,194 (37,052 to 145,343)	109.13(45.83 to 180.03)	3.42(3.29 to 3.55)
Liver cancer	Male	Europe	96,282 (38,113 to 170,958)	107.38(42.5 to 190.68)	1.86(1.58 to 2.14)
Liver cancer	Male	Africa	45,702 (17,919 to 80,791)	126.34(49.59 to 222.61)	3.14(3.01 to 3.27)
Multiple myeloma	Male	Global	73,499 (-28,976 to 187,926)	14.95(-5.86 to 38.28)	1.07(0.93 to 1.2)
Multiple myeloma	Male	High SDI	40,339 (-17,192 to 100,642)	31.95(-13.64 to 79.71)	0.44(0.26 to 0.61)
Multiple myeloma	Male	High-middle SDI	16,780 (-6,767 to 43,285)	14.89(-5.96 to 38.43)	1.47(1.31 to 1.63)
Multiple myeloma	Male	Middle SDI	11,512 (-4,050 to 30,029)	7.31(-2.55 to 19.1)	3.56(3.29 to 3.82)
Multiple myeloma	Male	Low-middle SDI	4,017 (-1,215 to 10,581)	4.87(-1.46 to 12.83)	4.05(3.77 to 4.34)
Multiple myeloma	Male	Low SDI	751 (-145 to 2,044)	2.66(-0.51 to 7.24)	3.23(3.13 to 3.34)
Multiple myeloma	Male	High-income Asia Pacific	2,047 (-463 to 5,355)	7.45(-1.72 to 19.53)	0.36(0.16 to 0.57)
Multiple myeloma	Male	High-income North America	19,527 (-8,738 to 48,751)	48.42(-21.63 to 120.99)	0.28(0.14 to 0.43)

Cause	Sex	Location	DALYs (95%CI)	ASDR (per 100,000 population, 95%CI)	AAPC (1990 – 2021, 95%CI)
Multiple myeloma	Male	Western Europe	19,354 (-7,915 to 48,884)	34.86(-14.32 to 87.98)	0.67(0.54 to 0.81)
Multiple myeloma	Male	Australasia	1,570 (-685 to 4,025)	46.73(-20.41 to 119.71)	1.18(0.38 to 1.98)
Multiple myeloma	Male	Andean Latin America	650 (-262 to 1,752)	18.85(-7.6 to 50.87)	2.23(1.12 to 3.36)
Multiple myeloma	Male	Tropical Latin America	3,027 (-1,223 to 7,750)	21.13(-8.5 to 54.09)	2.62(2.1 to 3.13)
Multiple myeloma	Male	Central Latin America	2,583 (-1,107 to 6,639)	18(-7.69 to 46.32)	2.42(1.71 to 3.14)
Multiple myeloma	Male	Southern Latin America	1,457 (-633 to 3,715)	29.52(-12.82 to 75.29)	0.91(0.48 to 1.34)
Multiple myeloma	Male	Caribbean	694 (-268 to 1,838)	22.25(-8.57 to 58.9)	1.99(1.76 to 2.22)
Multiple myeloma	Male	Central Europe	3,669 (-1,625 to 9,384)	28.53(-12.59 to 72.95)	1.49(1.21 to 1.78)
Multiple myeloma	Male	Eastern Europe	3,010 (-1,266 to 7,709)	16.44(-6.88 to 42.1)	2.15(1.72 to 2.59)
Multiple myeloma	Male	Central Asia	216 (-89 to 550)	4.77(-1.93 to 12.19)	2.01(1.35 to 2.67)
Multiple myeloma	Male	North Africa and Middle East	4,132 (-1,789 to 11,004)	15.77(-6.77 to 42.14)	2.8(2.72 to 2.89)
Multiple myeloma	Male	South Asia	3,592 (-908 to 9,912)	4.09(-1.04 to 11.3)	4.49(4.37 to 4.61)
Multiple myeloma	Male	Southeast Asia	702 (-175 to 2,034)	1.87(-0.46 to 5.45)	3.86(3.71 to 4.01)
Multiple myeloma	Male	East Asia	5,877 (-1,594 to 16,071)	4.36(-1.18 to 11.95)	6.75(6.43 to 7.07)
Multiple myeloma	Male	Oceania	15 (-6 to 37)	3.49(-1.33 to 8.95)	0.65(0.37 to 0.94)
Multiple myeloma	Male	Western Sub-Saharan Africa	229 (-68 to 633)	2.32(-0.69 to 6.4)	3.57(3.39 to 3.74)
Multiple myeloma	Male	Eastern Sub-Saharan Africa	436 (-87 to 1,256)	4.89(-0.95 to 14.05)	3.42(3.37 to 3.47)
Multiple myeloma	Male	Central Sub-Saharan Africa	76 (-20 to 221)	2.87(-0.75 to 8.45)	2.88(2.75 to 3.02)
Multiple myeloma	Male	Southern Sub-Saharan Africa	636 (-254 to 1,653)	22.88(-9.14 to 59.69)	2.84(2.49 to 3.2)
Multiple myeloma	Male	Four World Regions	73,228 (-28,854 to 187,217)	14.93(-5.85 to 38.21)	1.07(0.93 to 1.2)
Multiple myeloma	Male	Asia	15,558 (-4,445 to 41,398)	5.28(-1.5 to 14.03)	3.59(3.36 to 3.82)
Multiple myeloma	Male	America	27,744 (-12,280 to 69,383)	35.05(-15.48 to 87.75)	0.64(0.54 to 0.74)

Cause	Sex	Location	DALYs (95%CI)	ASDR (per 100,000 population, 95%CI)	AAPC (1990 – 2021, 95%CI)
Multiple myeloma	Male	Europe	27,552 (-11,591 to 70,000)	30.68(-12.91 to 77.94)	1.15(1.02 to 1.27)
Multiple myeloma	Male	Africa	2,374 (-825 to 6,168)	6.85(-2.36 to 17.86)	3.25(3.12 to 3.39)
Non-Hodgkin lymphoma	Male	Global	97,872 (32,405 to 169,787)	19.84(6.58 to 34.42)	0.49(0.4 to 0.58)
Non-Hodgkin lymphoma	Male	High SDI	46,540 (15,459 to 80,726)	36.91(12.26 to 63.99)	-0.32(-0.42 to -0.23)
Non-Hodgkin lymphoma	Male	High-middle SDI	21,849 (6,986 to 38,701)	19.23(6.16 to 34.01)	0.73(0.57 to 0.89)
Non-Hodgkin lymphoma	Male	Middle SDI	19,173 (6,575 to 32,862)	12.22(4.19 to 20.96)	1.73(1.64 to 1.82)
Non-Hodgkin lymphoma	Male	Low-middle SDI	7,737 (2,544 to 13,516)	9.39(3.1 to 16.4)	1.95(1.91 to 2)
Non-Hodgkin lymphoma	Male	Low SDI	2,469 (809 to 4,301)	8.85(2.92 to 15.4)	1.14(1.05 to 1.23)
Non-Hodgkin lymphoma	Male	High-income Asia Pacific	4,860 (1,689 to 8,377)	17.5(6.05 to 30.15)	0.12(-0.03 to 0.28)
Non-Hodgkin lymphoma	Male	High-income North America	22,141 (7,107 to 37,927)	55.02(17.67 to 94.28)	-0.84(-0.96 to -0.72)
Non-Hodgkin lymphoma	Male	Western Europe	19,372 (6,407 to 33,807)	35.02(11.6 to 61.07)	-0.1(-0.41 to 0.22)
Non-Hodgkin lymphoma	Male	Australasia	1,570 (514 to 2,881)	46.79(15.31 to 85.9)	-0.34(-0.64 to -0.05)
Non-Hodgkin lymphoma	Male	Andean Latin America	1,164 (367 to 2,187)	33.94(10.7 to 63.82)	1.55(1.01 to 2.11)
Non-Hodgkin lymphoma	Male	Tropical Latin America	2,747 (869 to 4,749)	19.19(6.08 to 33.2)	0.87(0.54 to 1.21)
Non-Hodgkin lymphoma	Male	Central Latin America	3,332 (1,111 to 5,840)	23.53(7.85 to 41.27)	1.75(1.41 to 2.09)
Non-Hodgkin lymphoma	Male	Southern Latin America	1,561 (495 to 2,765)	31.62(10.02 to 56.03)	0.18(-0.07 to 0.43)
Non-Hodgkin lymphoma	Male	Caribbean	638 (211 to 1,113)	20.42(6.75 to 35.6)	0.62(0.14 to 1.1)
Non-Hodgkin lymphoma	Male	Central Europe	3,747 (1,239 to 6,528)	29.18(9.63 to 50.84)	0.69(0.47 to 0.92)
Non-Hodgkin lymphoma	Male	Eastern Europe	3,833 (1,202 to 6,684)	21.14(6.67 to 36.92)	1.67(1.05 to 2.3)
Non-Hodgkin lymphoma	Male	Central Asia	372 (120 to 654)	8.32(2.7 to 14.58)	-0.61(-1.02 to -0.19)
Non-Hodgkin lymphoma	Male	North Africa and Middle East	6,012 (1,827 to 10,901)	22.99(6.99 to 41.64)	1.19(1.05 to 1.33)
Non-Hodgkin lymphoma	Male	South Asia	6,041 (1,969 to 10,561)	6.92(2.27 to 12.1)	2(1.86 to 2.14)

Cause	Sex	Location	DALYs (95%CI)	ASDR (per 100,000 population, 95%CI)	AAPC (1990 – 2021, 95%CI)
Non-Hodgkin lymphoma	Male	Southeast Asia	2,704 (934 to 4,879)	7.2(2.49 to 13.07)	1.74(1.6 to 1.88)
Non-Hodgkin lymphoma	Male	East Asia	14,357 (4,779 to 26,462)	10.73(3.56 to 19.75)	1.82(1.57 to 2.07)
Non-Hodgkin lymphoma	Male	Oceania	30 (9 to 54)	7.35(2.2 to 13.43)	0.66(0.47 to 0.84)
Non-Hodgkin lymphoma	Male	Western Sub-Saharan Africa	1,209 (393 to 2,143)	11.85(3.85 to 21.03)	2.09(2.03 to 2.15)
Non-Hodgkin lymphoma	Male	Eastern Sub-Saharan Africa	1,218 (409 to 2,149)	14.08(4.77 to 24.89)	0.92(0.82 to 1.03)
Non-Hodgkin lymphoma	Male	Central Sub-Saharan Africa	327 (94 to 615)	12.39(3.59 to 23.56)	1.93(1.82 to 2.03)
Non-Hodgkin lymphoma	Male	Southern Sub-Saharan Africa	637 (187 to 1,135)	23.17(6.83 to 41.3)	2.27(1.97 to 2.57)
Non-Hodgkin lymphoma	Male	Four World Regions	97,616 (32,315 to 169,363)	19.83(6.57 to 34.4)	0.49(0.4 to 0.58)
Non-Hodgkin lymphoma	Male	Asia	32,288 (11,067 to 56,187)	10.96(3.76 to 19.07)	1.53(1.46 to 1.6)
Non-Hodgkin lymphoma	Male	America	31,411 (10,273 to 54,315)	39.76(13.01 to 68.77)	-0.32(-0.4 to -0.24)
Non-Hodgkin lymphoma	Male	Europe	28,496 (9,359 to 49,800)	31.77(10.43 to 55.51)	0.39(0.21 to 0.56)
Non-Hodgkin lymphoma	Male	Africa	5,421 (1,745 to 9,550)	15.7(5.07 to 27.62)	1.65(1.59 to 1.72)
Leukemia	Male	Global	191,422 (139,577 to 249,783)	39.33(28.54 to 51.39)	-0.09(-0.12 to -0.06)
Leukemia	Male	High SDI	85,359 (62,490 to 109,920)	67.47(49.39 to 86.87)	-0.6(-0.66 to -0.53)
Leukemia	Male	High-middle SDI	49,205 (35,197 to 65,597)	44.15(31.48 to 58.87)	-0.16(-0.39 to 0.07)
Leukemia	Male	Middle SDI	37,702 (25,753 to 52,733)	24.57(16.75 to 34.42)	0.68(0.53 to 0.83)
Leukemia	Male	Low-middle SDI	15,344 (10,417 to 21,746)	19.06(12.92 to 27.14)	1.51(1.43 to 1.58)
Leukemia	Male	Low SDI	3,560 (2,044 to 5,477)	13.17(7.55 to 20.31)	0.87(0.77 to 0.98)
Leukemia	Male	High-income Asia Pacific	7,508 (5,287 to 10,226)	27.45(19.38 to 37.32)	-0.85(-1.13 to -0.58)
Leukemia	Male	High-income North America	38,439 (28,093 to 49,299)	95.98(69.99 to 123.23)	-0.61(-0.86 to -0.36)
Leukemia	Male	Western Europe	40,207 (28,807 to 53,215)	71.61(51.44 to 94.65)	-0.68(-0.83 to -0.54)
Leukemia	Male	Australasia	2,961 (2,070 to 4,042)	87.74(61.34 to 119.83)	-0.13(-0.56 to 0.31)

Cause	Sex	Location	DALYs (95%CI)	ASDR (per 100,000 population, 95%CI)	AAPC (1990 – 2021, 95%CI)
Leukemia	Male	Andean Latin America	1,386 (884 to 2,030)	40.77(25.98 to 59.75)	0.82(0.33 to 1.32)
Leukemia	Male	Tropical Latin America	6,026 (4,382 to 7,849)	43.22(31.32 to 56.42)	0.39(0.18 to 0.6)
Leukemia	Male	Central Latin America	5,368 (3,857 to 7,091)	38.42(27.57 to 50.73)	0.99(0.86 to 1.11)
Leukemia	Male	Southern Latin America	2,781 (1,987 to 3,721)	57.11(40.76 to 76.42)	-0.15(-0.49 to 0.2)
Leukemia	Male	Caribbean	1,109 (798 to 1,496)	35.7(25.69 to 48.18)	0.5(0.02 to 0.97)
Leukemia	Male	Central Europe	10,181 (7,473 to 13,264)	80.07(58.68 to 104.29)	-0.34(-0.41 to -0.28)
Leukemia	Male	Eastern Europe	9,920 (7,119 to 13,277)	56.32(40.43 to 75.2)	0.02(-0.44 to 0.48)
Leukemia	Male	Central Asia	1,179 (855 to 1,576)	27.08(19.66 to 36.14)	-0.64(-0.99 to -0.29)
Leukemia	Male	North Africa and Middle East	16,059 (10,023 to 23,226)	62.86(39.22 to 91.51)	0.78(0.71 to 0.85)
Leukemia	Male	South Asia	10,035 (6,198 to 14,816)	11.74(7.23 to 17.41)	1.33(1.24 to 1.42)
Leukemia	Male	Southeast Asia	6,324 (4,098 to 9,424)	17.62(11.33 to 26.47)	0.97(0.92 to 1.02)
Leukemia	Male	East Asia	28,249 (16,873 to 43,189)	21.3(12.78 to 32.63)	0.64(0.45 to 0.83)
Leukemia	Male	Oceania	98 (48 to 170)	23.95(11.87 to 41.63)	0.07(-0.04 to 0.18)
Leukemia	Male	Western Sub-Saharan Africa	687 (335 to 1,051)	7.01(3.41 to 10.73)	1.48(1.41 to 1.54)
Leukemia	Male	Eastern Sub-Saharan Africa	1,497 (851 to 2,490)	17.83(10.09 to 29.85)	0.54(0.43 to 0.66)
Leukemia	Male	Central Sub-Saharan Africa	352 (166 to 640)	14.17(6.49 to 26.44)	1.61(1.53 to 1.7)
Leukemia	Male	Southern Sub-Saharan Africa	1,056 (577 to 1,526)	40.04(21.55 to 58.22)	1.12(0.76 to 1.49)
Leukemia	Male	Four World Regions	190,912 (139,191 to 249,126)	39.3(28.52 to 51.35)	-0.09(-0.11 to -0.06)
Leukemia	Male	Asia	62,799 (41,212 to 89,167)	21.37(14.05 to 30.34)	0.54(0.39 to 0.68)
Leukemia	Male	America	54,818 (40,533 to 69,953)	70.03(51.65 to 89.49)	-0.19(-0.26 to -0.12)
Leukemia	Male	Europe	64,476 (47,237 to 84,096)	71.89(52.65 to 93.76)	-0.38(-0.52 to -0.23)
Leukemia	Male	Africa	8,820 (5,353 to 12,662)	26.1(15.85 to 37.75)	1.41(1.26 to 1.55)

Cause	Sex	Location	DALYs (95%CI)	ASDR (per 100,000 population, 95%CI)	AAPC (1990 – 2021, 95%CI)
Kidney cancer	Male	Global	311,042 (125,435 to 503,981)	62.62(25.18 to 101.61)	0.7(0.56 to 0.83)
Kidney cancer	Male	High SDI	148,540 (60,348 to 237,985)	118.53(48.15 to 189.75)	0.16(-0.05 to 0.37)
Kidney cancer	Male	High-middle SDI	98,080 (39,311 to 159,772)	85.83(34.32 to 140.07)	0.98(0.8 to 1.16)
Kidney cancer	Male	Middle SDI	47,254 (19,469 to 78,544)	30.35(12.46 to 50.57)	2.87(2.76 to 2.97)
Kidney cancer	Male	Low-middle SDI	13,699 (5,447 to 22,259)	16.53(6.55 to 26.93)	3.56(3.39 to 3.73)
Kidney cancer	Male	Low SDI	2,970 (1,026 to 5,128)	10.51(3.63 to 18.11)	2.73(2.67 to 2.79)
Kidney cancer	Male	High-income Asia Pacific	10,240 (3,853 to 16,857)	37.68(14.12 to 62.05)	0.94(0.65 to 1.23)
Kidney cancer	Male	High-income North America	61,877 (25,622 to 96,164)	152.98(63.25 to 237.93)	0.22(-0.04 to 0.47)
Kidney cancer	Male	Western Europe	70,932 (28,123 to 115,944)	129.69(51.52 to 211.73)	0.09(-0.02 to 0.2)
Kidney cancer	Male	Australasia	4,248 (1,724 to 6,993)	127.78(51.85 to 210.07)	0.83(0.68 to 0.98)
Kidney cancer	Male	Andean Latin America	2,184 (896 to 3,837)	63.57(26.03 to 111.76)	2.24(1.74 to 2.74)
Kidney cancer	Male	Tropical Latin America	10,630 (4,118 to 17,714)	73.95(28.57 to 123.23)	2.65(2.13 to 3.17)
Kidney cancer	Male	Central Latin America	13,342 (5,643 to 21,759)	93.41(39.42 to 152.44)	2.84(2.68 to 2.99)
Kidney cancer	Male	Southern Latin America	10,738 (4,273 to 17,627)	217.08(86.37 to 356.53)	1.45(1.23 to 1.68)
Kidney cancer	Male	Caribbean	1,552 (608 to 2,596)	49.65(19.45 to 83.1)	2.02(0.9 to 3.15)
Kidney cancer	Male	Central Europe	24,750 (10,045 to 40,394)	192.68(78.14 to 314.8)	0.96(0.82 to 1.1)
Kidney cancer	Male	Eastern Europe	33,561 (13,439 to 55,171)	183.92(73.41 to 302.15)	1.69(1.32 to 2.05)
Kidney cancer	Male	Central Asia	3,676 (1,420 to 6,187)	82.28(31.8 to 138.57)	1.98(1.55 to 2.41)
Kidney cancer	Male	North Africa and Middle East	13,614 (5,810 to 22,873)	51.84(21.99 to 87.39)	2.48(2.42 to 2.54)
Kidney cancer	Male	South Asia	7,920 (2,923 to 13,398)	8.95(3.3 to 15.18)	3.71(3.6 to 3.81)
Kidney cancer	Male	Southeast Asia	4,840 (1,837 to 8,238)	12.75(4.83 to 21.72)	2.67(2.53 to 2.82)
Kidney cancer	Male	East Asia	31,971 (12,027 to 56,701)	24.96(9.39 to 44.24)	3.45(3.1 to 3.8)

Cause	Sex	Location	DALYs (95%CI)	ASDR (per 100,000 population, 95%CI)	AAPC (1990 – 2021, 95%CI)
Kidney cancer	Male	Oceania	46 (16 to 85)	11.05(3.89 to 20.14)	1.09(0.84 to 1.33)
Kidney cancer	Male	Western Sub-Saharan Africa	1,570 (602 to 2,672)	15.5(5.92 to 26.45)	3.29(3.18 to 3.39)
Kidney cancer	Male	Eastern Sub-Saharan Africa	1,433 (475 to 2,539)	16.05(5.32 to 28.37)	2.83(2.77 to 2.9)
Kidney cancer	Male	Central Sub-Saharan Africa	490 (156 to 1,027)	18.94(5.85 to 40.12)	3.16(3.07 to 3.26)
Kidney cancer	Male	Southern Sub-Saharan Africa	1,428 (543 to 2,312)	52.2(19.94 to 85.13)	2.58(2.3 to 2.86)
Kidney cancer	Male	Four World Regions	310,166 (125,077 to 502,566)	62.56(25.15 to 101.51)	0.7(0.56 to 0.83)
Kidney cancer	Male	Asia	67,564 (25,953 to 112,611)	23.22(8.92 to 38.74)	2.71(2.57 to 2.84)
Kidney cancer	Male	America	99,893 (40,930 to 157,220)	125.24(51.23 to 197.3)	0.74(0.57 to 0.91)
Kidney cancer	Male	Europe	134,462 (54,022 to 219,459)	149.99(60.27 to 244.76)	0.67(0.51 to 0.83)
Kidney cancer	Male	Africa	8,248 (3,220 to 13,396)	23.73(9.27 to 38.68)	3.01(2.96 to 3.06)
Thyroid cancer	Male	Global	28,363 (20,945 to 36,561)	5.77(4.24 to 7.45)	0.87(0.7 to 1.04)
Thyroid cancer	Male	High SDI	9,253 (6,852 to 11,762)	7.37(5.46 to 9.36)	0.04(-0.36 to 0.43)
Thyroid cancer	Male	High-middle SDI	6,653 (4,894 to 8,785)	6(4.39 to 7.94)	0.33(0.1 to 0.56)
Thyroid cancer	Male	Middle SDI	8,330 (5,747 to 11,123)	5.58(3.82 to 7.5)	2.09(1.94 to 2.24)
Thyroid cancer	Male	Low-middle SDI	3,162 (2,283 to 4,242)	3.9(2.81 to 5.23)	2.4(2.29 to 2.51)
Thyroid cancer	Male	Low SDI	936 (597 to 1,357)	3.39(2.16 to 4.9)	1.5(1.46 to 1.54)
Thyroid cancer	Male	High-income Asia Pacific	1,389 (968 to 1,908)	5.06(3.53 to 6.96)	-0.05(-0.51 to 0.41)
Thyroid cancer	Male	High-income North America	3,910 (2,931 to 4,956)	9.7(7.26 to 12.31)	0.89(0.36 to 1.42)
Thyroid cancer	Male	Western Europe	3,924 (2,836 to 5,080)	7.19(5.22 to 9.3)	-0.66(-1.48 to 0.16)
Thyroid cancer	Male	Australasia	269 (184 to 368)	8.11(5.54 to 11.11)	1.14(0.61 to 1.67)
Thyroid cancer	Male	Andean Latin America	393 (259 to 585)	11.53(7.59 to 17.14)	1.84(0.97 to 2.73)
Thyroid cancer	Male	Tropical Latin America	929 (677 to 1,211)	6.71(4.87 to 8.77)	0.67(0.08 to 1.27)

Cause	Sex	Location	DALYs (95%CI)	ASDR (per 100,000 population, 95%CI)	AAPC (1990 – 2021, 95%CI)
Thyroid cancer	Male	Central Latin America	1,456 (1,058 to 1,907)	10.34(7.51 to 13.55)	1.2(0.93 to 1.47)
Thyroid cancer	Male	Southern Latin America	479 (339 to 643)	9.77(6.9 to 13.13)	-0.12(-1.24 to 1.02)
Thyroid cancer	Male	Caribbean	223 (158 to 303)	7.17(5.06 to 9.75)	1.82(0.56 to 3.08)
Thyroid cancer	Male	Central Europe	876 (651 to 1,120)	6.83(5.07 to 8.73)	-2.17(-2.85 to -1.5)
Thyroid cancer	Male	Eastern Europe	1,287 (948 to 1,678)	7.25(5.33 to 9.45)	1.01(-0.01 to 2.03)
Thyroid cancer	Male	Central Asia	211 (155 to 278)	5(3.66 to 6.57)	-1.04(-2.82 to 0.77)
Thyroid cancer	Male	North Africa and Middle East	1,812 (1,252 to 2,436)	6.95(4.8 to 9.35)	2.13(2.04 to 2.22)
Thyroid cancer	Male	South Asia	2,996 (2,027 to 4,167)	3.45(2.33 to 4.8)	2.68(2.54 to 2.82)
Thyroid cancer	Male	Southeast Asia	1,778 (1,204 to 2,477)	4.81(3.25 to 6.74)	1.99(1.95 to 2.04)
Thyroid cancer	Male	East Asia	5,570 (3,368 to 8,121)	4.68(2.8 to 6.82)	2.27(2.11 to 2.42)
Thyroid cancer	Male	Oceania	19 (11 to 29)	4.48(2.55 to 7.03)	0.63(0.43 to 0.83)
Thyroid cancer	Male	Western Sub-Saharan Africa	61 (41 to 96)	0.59(0.39 to 0.93)	0.73(0.64 to 0.83)
Thyroid cancer	Male	Eastern Sub-Saharan Africa	566 (319 to 871)	6.52(3.7 to 10.04)	1.41(1.37 to 1.45)
Thyroid cancer	Male	Central Sub-Saharan Africa	63 (35 to 103)	2.53(1.41 to 4.21)	1.38(1.24 to 1.52)
Thyroid cancer	Male	Southern Sub-Saharan Africa	151 (104 to 205)	5.62(3.83 to 7.6)	1.92(1.64 to 2.2)
Thyroid cancer	Male	Four World Regions	28,298 (20,894 to 36,483)	5.77(4.23 to 7.45)	0.87(0.7 to 1.04)
Thyroid cancer	Male	Asia	12,883 (8,731 to 17,562)	4.52(3.05 to 6.17)	2.04(1.85 to 2.22)
Thyroid cancer	Male	America	7,349 (5,547 to 9,225)	9.29(6.99 to 11.67)	0.91(0.53 to 1.3)
Thyroid cancer	Male	Europe	6,625 (4,919 to 8,492)	7.4(5.49 to 9.48)	-0.45(-0.88 to -0.02)
Thyroid cancer	Male	Africa	1,442 (970 to 1,996)	4.18(2.81 to 5.79)	1.75(1.69 to 1.81)
Breast cancer	Female	Global	761,088 (-22,395 to 1,528,907)	129.94(-3.83 to 260.98)	0.19(0.06 to 0.31)

Cause	Sex	Location	DALYs (95%CI)	ASDR (per 100,000 population, 95%CI)	AAPC (1990 – 2021, 95%CI)
Breast cancer	Female	High SDI	274,154 (-8,084 to 543,552)	183.55(-5.49 to 362.24)	-0.57(-0.64 to -0.49)
Breast cancer	Female	High-middle SDI	220,050 (-6,436 to 438,515)	155.34(-4.54 to 309.33)	0.36(0.22 to 0.51)
Breast cancer	Female	Middle SDI	164,507 (-4,859 to 338,017)	92.58(-2.73 to 190.33)	2.06(1.96 to 2.17)
Breast cancer	Female	Low-middle SDI	79,547 (-2,363 to 162,592)	85.9(-2.55 to 175.78)	3.22(3.1 to 3.34)
Breast cancer	Female	Low SDI	21,676 (-619 to 44,535)	72.42(-2.06 to 148.84)	2.09(1.98 to 2.21)
Breast cancer	Female	High-income Asia Pacific	16,474 (-407 to 33,863)	54.17(-1.36 to 110.82)	1.69(1.53 to 1.86)
Breast cancer	Female	High-income North America	117,649 (-3,746 to 229,791)	244.13(-7.86 to 475.62)	-0.91(-1.07 to -0.74)
Breast cancer	Female	Western Europe	137,295 (-3,812 to 279,463)	205.49(-5.74 to 415.97)	-0.64(-0.81 to -0.47)
Breast cancer	Female	Australasia	7,574 (-225 to 15,347)	202.76(-6.04 to 409.42)	-0.62(-1.12 to -0.12)
Breast cancer	Female	Andean Latin America	5,012 (-130 to 10,569)	132(-3.41 to 278.42)	1.94(1.37 to 2.51)
Breast cancer	Female	Tropical Latin America	30,236 (-887 to 60,176)	167.69(-4.91 to 333.78)	0.83(0.7 to 0.96)
Breast cancer	Female	Central Latin America	27,519 (-876 to 55,131)	162.2(-5.16 to 325.28)	1.83(1.59 to 2.07)

Cause	Sex	Location	DALYs (95%CI)	ASDR (per 100,000 population, 95%CI)	AAPC (1990 – 2021, 95%CI)
Breast cancer	Female	Southern Latin America	17,250 (-450 to 34,337)	273.34(-7.13 to 543.25)	-0.12(-0.45 to 0.22)
Breast cancer	Female	Caribbean	6,775 (-195 to 14,067)	188.89(-5.45 to 392.19)	1.11(0.68 to 1.54)
Breast cancer	Female	Central Europe	45,631 (-1,383 to 91,442)	264.85(-8.04 to 530.08)	0.89(0.74 to 1.03)
Breast cancer	Female	Eastern Europe	74,122 (-2,102 to 146,232)	244.44(-6.94 to 481.94)	1.37(0.8 to 1.95)
Breast cancer	Female	Central Asia	9,377 (-272 to 18,818)	159.53(-4.63 to 320.6)	0.04(-0.48 to 0.57)
Breast cancer	Female	North Africa and Middle East	43,655 (-1,351 to 87,006)	163.99(-5.05 to 327.48)	3.4(3.17 to 3.63)
Breast cancer	Female	South Asia	48,879 (-1,521 to 100,795)	51.83(-1.62 to 107.2)	4.11(3.82 to 4.4)
Breast cancer	Female	Southeast Asia	38,259 (-984 to 81,040)	84.6(-2.18 to 179.13)	2.92(2.83 to 3)
Breast cancer	Female	East Asia	87,190 (-2,724 to 182,149)	59.81(-1.87 to 124.82)	2.38(2.26 to 2.49)
Breast cancer	Female	Oceania	737 (-26 to 1,509)	182.78(-6.3 to 374.89)	1.03(0.88 to 1.18)
Breast cancer	Female	Western Sub-Saharan Africa	22,225 (-590 to 45,172)	189.42(-4.97 to 384.56)	2.74(2.66 to 2.82)
Breast cancer	Female	Eastern Sub-Saharan Africa	9,682 (-268 to 20,491)	97.96(-2.69 to 207.55)	2.52(2.45 to 2.59)

Cause	Sex	Location	DALYs (95%CI)	ASDR (per 100,000 population, 95%CI)	AAPC (1990 – 2021, 95%CI)
Breast cancer	Female	Central Sub-Saharan Africa	3,816 (-97 to 8,689)	110.99(-2.81 to 252.04)	2.79(2.71 to 2.87)
Breast cancer	Female	Southern Sub-Saharan Africa	11,729 (-350 to 23,250)	285.37(-8.59 to 567.69)	2.82(2.26 to 3.39)
Breast cancer	Female	Four World Regions	758,766 (-22,329 to 1,524,171)	129.79(-3.82 to 260.67)	0.18(0.06 to 0.31)
Breast cancer	Female	Asia	221,244 (-6,593 to 447,775)	66.3(-1.97 to 134.26)	2.16(1.99 to 2.33)
Breast cancer	Female	America	203,075 (-6,245 to 401,866)	211.89(-6.54 to 418.85)	-0.5(-0.65 to -0.35)
Breast cancer	Female	Europe	269,132 (-7,629 to 540,682)	225.91(-6.43 to 452.21)	0.18(0.08 to 0.28)
Breast cancer	Female	Africa	65,315 (-1,862 to 130,427)	166.91(-4.74 to 333.85)	2.99(2.84 to 3.13)
Ovarian cancer	Female	Global	240,944 (57,492 to 429,629)	41.14(9.81 to 73.34)	0.61(0.5 to 0.72)
Ovarian cancer	Female	High SDI	95,406 (23,747 to 171,682)	65.66(16.47 to 117.73)	-0.38(-0.44 to -0.32)
Ovarian cancer	Female	High-middle SDI	74,828 (18,521 to 132,816)	53(13.1 to 94.02)	0.42(0.16 to 0.69)
Ovarian cancer	Female	Middle SDI	45,437 (10,595 to 82,406)	25.36(5.89 to 46.03)	3.34(3.25 to 3.43)
Ovarian cancer	Female	Low-middle SDI	20,401 (4,116 to 38,171)	21.73(4.36 to 40.7)	4.5(4.37 to 4.62)

Cause	Sex	Location	DALYs (95%CI)	ASDR (per 100,000 population, 95%CI)	AAPC (1990 – 2021, 95%CI)
Ovarian cancer	Female	Low SDI	4,481 (609 to 8,801)	14.32(1.87 to 28.24)	3.54(3.49 to 3.59)
Ovarian cancer	Female	High-income Asia Pacific	3,497 (378 to 7,105)	11.83(1.44 to 23.83)	1.39(1.17 to 1.61)
Ovarian cancer	Female	High-income North America	42,553 (11,563 to 74,395)	89.32(24.32 to 155.67)	-0.52(-0.68 to -0.35)
Ovarian cancer	Female	Western Europe	44,001 (10,187 to 81,551)	68.98(16.02 to 127.45)	-0.52(-0.78 to -0.26)
Ovarian cancer	Female	Australasia	2,624 (668 to 4,916)	70.96(18.16 to 132.55)	-1.27(-2.09 to -0.45)
Ovarian cancer	Female	Andean Latin America	1,845 (451 to 3,567)	48.57(11.85 to 93.94)	3.61(3.24 to 3.98)
Ovarian cancer	Female	Tropical Latin America	8,379 (1,956 to 15,481)	46.49(10.85 to 85.91)	1.43(1.3 to 1.57)
Ovarian cancer	Female	Central Latin America	10,672 (2,875 to 19,597)	62.77(16.88 to 115.33)	2.55(2.2 to 2.9)
Ovarian cancer	Female	Southern Latin America	4,622 (1,173 to 8,329)	74.84(19.08 to 134.46)	0.53(0.26 to 0.81)
Ovarian cancer	Female	Caribbean	1,564 (377 to 2,899)	43.97(10.63 to 81.5)	2.45(2.14 to 2.76)
Ovarian cancer	Female	Central Europe	18,498 (4,931 to 33,583)	109.54(29.29 to 198.85)	0.73(0.65 to 0.81)
Ovarian cancer	Female	Eastern Europe	28,693 (7,489 to 50,461)	95.01(24.74 to 167.07)	0.62(0.06 to 1.19)

Cause	Sex	Location	DALYs (95%CI)	ASDR (per 100,000 population, 95%CI)	AAPC (1990 – 2021, 95%CI)
Ovarian cancer	Female	Central Asia	3,282 (843 to 6,056)	55.13(14.06 to 101.98)	1.84(1.54 to 2.15)
Ovarian cancer	Female	North Africa and Middle East	14,924 (4,284 to 26,628)	55.44(15.79 to 99.12)	2.26(2.2 to 2.32)
Ovarian cancer	Female	South Asia	14,876 (2,234 to 29,861)	15.5(2.29 to 31.23)	5.48(5.4 to 5.56)
Ovarian cancer	Female	Southeast Asia	7,700 (1,276 to 14,846)	16.81(2.73 to 32.52)	4.65(4.55 to 4.76)
Ovarian cancer	Female	East Asia	23,312 (4,436 to 47,577)	15.79(3 to 32.2)	5.37(5.11 to 5.63)
Ovarian cancer	Female	Oceania	71 (17 to 139)	17.08(3.96 to 33.61)	1.85(1.74 to 1.96)
Ovarian cancer	Female	Western Sub-Saharan Africa	2,876 (621 to 5,630)	23.8(5.1 to 46.65)	3.37(3.25 to 3.48)
Ovarian cancer	Female	Eastern Sub-Saharan Africa	2,650 (417 to 5,358)	25.09(3.82 to 50.86)	3.78(3.72 to 3.85)
Ovarian cancer	Female	Central Sub-Saharan Africa	684 (120 to 1,444)	19.15(3.23 to 40.79)	4.67(4.62 to 4.71)
Ovarian cancer	Female	Southern Sub-Saharan Africa	3,622 (962 to 6,516)	85.88(22.75 to 154.91)	2.53(2.34 to 2.72)
Ovarian cancer	Female	Four World Regions	240,232 (57,310 to 428,349)	41.1(9.8 to 73.25)	0.61(0.49 to 0.72)
Ovarian cancer	Female	Asia	59,367 (10,558 to 112,877)	17.68(3.14 to 33.6)	3.86(3.78 to 3.94)

Cause	Sex	Location	DALYs (95%CI)	ASDR (per 100,000 population, 95%CI)	AAPC (1990 – 2021, 95%CI)
Ovarian cancer	Female	America	69,266 (18,498 to 123,208)	72.71(19.44 to 129.21)	0.27(0.15 to 0.4)
Ovarian cancer	Female	Europe	96,881 (24,384 to 172,631)	83.86(21.21 to 148.91)	0.17(-0.05 to 0.39)
Ovarian cancer	Female	Africa	14,718 (3,708 to 26,784)	36.43(9.09 to 66.36)	3.22(3.11 to 3.33)
Uterine cancer	Female	Global	560,805 (399,074 to 735,910)	95.61(68.05 to 125.45)	0.33(0.18 to 0.47)
Uterine cancer	Female	High SDI	215,064 (152,702 to 280,278)	147.09(105.24 to 190.94)	0.86(0.7 to 1.03)
Uterine cancer	Female	High-middle SDI	179,604 (126,547 to 236,328)	126.54(89.2 to 166.42)	-0.12(-0.52 to 0.28)
Uterine cancer	Female	Middle SDI	103,503 (71,191 to 142,220)	58(39.85 to 79.69)	0.97(0.81 to 1.14)
Uterine cancer	Female	Low-middle SDI	47,969 (32,303 to 65,706)	51.72(34.79 to 70.95)	1.8(1.69 to 1.91)
Uterine cancer	Female	Low SDI	13,633 (8,752 to 19,743)	44.57(28.59 to 64.65)	1.4(1.35 to 1.45)
Uterine cancer	Female	High-income Asia Pacific	10,909 (7,024 to 15,375)	36.11(23.7 to 50.62)	0.87(0.68 to 1.06)
Uterine cancer	Female	High-income North America	111,567 (79,895 to 142,957)	234.63(168.49 to 300.06)	1.5(1.2 to 1.8)
Uterine cancer	Female	Western Europe	86,104 (59,298 to 116,591)	131.57(91.53 to 177.09)	0.47(0.27 to 0.67)

Cause	Sex	Location	DALYs (95%CI)	ASDR (per 100,000 population, 95%CI)	AAPC (1990 – 2021, 95%CI)
Uterine cancer	Female	Australasia	4,544 (3,079 to 6,265)	122.79(83.56 to 169.02)	1.07(0.53 to 1.61)
Uterine cancer	Female	Andean Latin America	5,716 (3,523 to 8,794)	150.71(92.91 to 231.85)	0.11(-0.7 to 0.93)
Uterine cancer	Female	Tropical Latin America	21,750 (14,981 to 29,392)	120.78(83.18 to 163.22)	0.36(0.2 to 0.52)
Uterine cancer	Female	Central Latin America	19,383 (13,471 to 26,294)	114.1(79.28 to 154.85)	1.14(0.85 to 1.42)
Uterine cancer	Female	Southern Latin America	7,561 (5,151 to 10,394)	121.22(82.8 to 166.46)	-0.63(-1.02 to -0.24)
Uterine cancer	Female	Caribbean	8,350 (5,717 to 11,510)	233.75(160.14 to 322.1)	1.88(1 to 2.76)
Uterine cancer	Female	Central Europe	40,571 (28,736 to 54,243)	234.49(166.31 to 313.03)	0.31(-0.05 to 0.67)
Uterine cancer	Female	Eastern Europe	86,555 (60,537 to 114,526)	285(199.44 to 377.11)	0.77(0.24 to 1.3)
Uterine cancer	Female	Central Asia	8,298 (5,832 to 11,147)	142.22(99.9 to 191.27)	-0.67(-1.52 to 0.18)
Uterine cancer	Female	North Africa and Middle East	21,368 (14,009 to 29,572)	81.09(52.93 to 112.47)	0.71(0.61 to 0.81)
Uterine cancer	Female	South Asia	27,427 (16,675 to 41,194)	28.94(17.58 to 43.59)	2.63(2.51 to 2.75)
Uterine cancer	Female	Southeast Asia	21,702 (13,189 to 31,108)	47.65(29.02 to 68.39)	2.24(2.11 to 2.37)

Cause	Sex	Location	DALYs (95%CI)	ASDR (per 100,000 population, 95%CI)	AAPC (1990 – 2021, 95%CI)
Uterine cancer	Female	East Asia	54,410 (32,888 to 84,982)	36.95(22.33 to 57.72)	0.68(0.56 to 0.8)
Uterine cancer	Female	Oceania	586 (311 to 910)	139.56(74.59 to 216.07)	1.01(0.86 to 1.15)
Uterine cancer	Female	Western Sub-Saharan Africa	8,709 (5,440 to 12,466)	73.99(46.27 to 105.93)	1.66(1.57 to 1.74)
Uterine cancer	Female	Eastern Sub-Saharan Africa	5,820 (3,465 to 9,000)	56.83(33.83 to 87.87)	1.43(1.36 to 1.51)
Uterine cancer	Female	Central Sub-Saharan Africa	2,388 (1,297 to 4,064)	68.14(36.81 to 117.14)	2.13(2.08 to 2.17)
Uterine cancer	Female	Southern Sub-Saharan Africa	7,088 (4,486 to 9,761)	170.02(107.06 to 234.58)	2.17(1.84 to 2.5)
Uterine cancer	Female	Four World Regions	558,829 (397,687 to 733,368)	95.45(67.94 to 125.25)	0.32(0.18 to 0.47)
Uterine cancer	Female	Asia	132,307 (86,301 to 185,178)	39.48(25.72 to 55.28)	1.13(1.06 to 1.19)
Uterine cancer	Female	America	173,133 (122,933 to 224,391)	181.59(129.11 to 235.16)	0.98(0.84 to 1.12)
Uterine cancer	Female	Europe	223,441 (156,742 to 294,774)	190.8(134.35 to 251.05)	0.39(0.02 to 0.77)
Uterine cancer	Female	Africa	29,947 (19,484 to 41,196)	75.76(49.15 to 104.19)	1.59(1.5 to 1.67)
Colon and rectum cancer	Female	Global	710,664 (303,965 to 1,127,081)	120.91(51.71 to 191.71)	-0.1(-0.24 to 0.05)

Cause	Sex	Location	DALYs (95%CI)	ASDR (per 100,000 population, 95%CI)	AAPC (1990 – 2021, 95%CI)
Colon and rectum cancer	Female	High SDI	252,425 (107,918 to 404,828)	159.72(68.9 to 255)	-0.6(-0.79 to -0.42)
Colon and rectum cancer	Female	High-middle SDI	252,008 (108,767 to 400,976)	175.99(75.98 to 279.94)	-0.01(-0.31 to 0.28)
Colon and rectum cancer	Female	Middle SDI	147,954 (63,616 to 237,478)	84.57(36.27 to 135.8)	1.57(1.46 to 1.68)
Colon and rectum cancer	Female	Low-middle SDI	46,767 (19,768 to 74,612)	51.85(21.87 to 82.85)	2.08(1.94 to 2.22)
Colon and rectum cancer	Female	Low SDI	10,348 (4,024 to 16,876)	35.45(13.7 to 57.91)	1.01(0.91 to 1.1)
Colon and rectum cancer	Female	High-income Asia Pacific	28,821 (10,805 to 48,050)	76.03(28.89 to 125.71)	-0.26(-0.56 to 0.04)
Colon and rectum cancer	Female	High-income North America	89,088 (39,670 to 138,954)	180.86(80.78 to 281.02)	-0.5(-0.82 to -0.17)
Colon and rectum cancer	Female	Western Europe	120,746 (50,857 to 199,247)	168.09(71.28 to 276.02)	-0.84(-0.99 to -0.69)
Colon and rectum cancer	Female	Australasia	7,487 (3,249 to 12,273)	190.81(83.03 to 311.54)	-0.92(-1.09 to -0.76)
Colon and rectum cancer	Female	Andean Latin America	5,508 (2,303 to 9,470)	145.6(60.87 to 250.32)	1.56(0.61 to 2.52)
Colon and rectum cancer	Female	Tropical Latin America	26,625 (11,311 to 42,606)	148.17(62.94 to 237.11)	1.55(1.43 to 1.66)
Colon and rectum cancer	Female	Central Latin America	22,880 (10,160 to 37,143)	136.47(60.53 to 221.72)	1.76(1.48 to 2.04)

Cause	Sex	Location	DALYs (95%CI)	ASDR (per 100,000 population, 95%CI)	AAPC (1990 – 2021, 95%CI)
Colon and rectum cancer	Female	Southern Latin America	16,525 (7,264 to 27,019)	255.29(112.41 to 416.72)	0.55(0.31 to 0.79)
Colon and rectum cancer	Female	Caribbean	7,036 (2,997 to 11,539)	194.04(82.75 to 318.08)	1.4(1.35 to 1.45)
Colon and rectum cancer	Female	Central Europe	49,723 (22,046 to 79,975)	280.35(124.75 to 450.54)	0.11(0.01 to 0.21)
Colon and rectum cancer	Female	Eastern Europe	89,970 (39,345 to 141,381)	294.99(128.91 to 463.15)	0.38(-0.24 to 1.02)
Colon and rectum cancer	Female	Central Asia	6,439 (2,807 to 10,172)	115.05(50.04 to 182.21)	-0.57(-0.75 to -0.39)
Colon and rectum cancer	Female	North Africa and Middle East	36,989 (16,193 to 58,624)	146.46(63.96 to 233)	0.79(0.64 to 0.94)
Colon and rectum cancer	Female	South Asia	20,099 (7,702 to 34,080)	21.55(8.25 to 36.67)	2.31(2.21 to 2.4)
Colon and rectum cancer	Female	Southeast Asia	29,074 (11,580 to 48,813)	67.2(26.59 to 112.97)	2.46(2.37 to 2.55)
Colon and rectum cancer	Female	East Asia	133,846 (53,238 to 228,390)	91.41(36.31 to 155.98)	1.67(1.47 to 1.87)
Colon and rectum cancer	Female	Oceania	279 (117 to 453)	72.19(30.22 to 117.33)	0.33(0.15 to 0.51)
Colon and rectum cancer	Female	Western Sub-Saharan Africa	6,046 (2,309 to 10,048)	55.43(21.26 to 91.94)	1.65(1.58 to 1.71)
Colon and rectum cancer	Female	Eastern Sub-Saharan Africa	5,180 (1,998 to 8,667)	52.88(20.26 to 88.61)	1.16(1.01 to 1.3)

Cause	Sex	Location	DALYs (95%CI)	ASDR (per 100,000 population, 95%CI)	AAPC (1990 – 2021, 95%CI)
Colon and rectum cancer	Female	Central Sub-Saharan Africa	1,759 (649 to 3,225)	53.39(19.49 to 98.43)	2.01(1.96 to 2.06)
Colon and rectum cancer	Female	Southern Sub-Saharan Africa	6,544 (2,772 to 10,315)	163.52(69.19 to 258.32)	1.82(1.5 to 2.14)
Colon and rectum cancer	Female	Four World Regions	708,427 (302,971 to 1,123,558)	120.77(51.64 to 191.49)	-0.1(-0.25 to 0.05)
Colon and rectum cancer	Female	Asia	237,029 (99,479 to 388,423)	71.88(30.1 to 117.85)	1.25(1.16 to 1.35)
Colon and rectum cancer	Female	America	166,362 (73,712 to 263,733)	171.8(76.26 to 271.96)	0.03(-0.15 to 0.21)
Colon and rectum cancer	Female	Europe	272,375 (117,543 to 436,943)	220.54(95.52 to 352.42)	-0.3(-0.44 to -0.16)
Colon and rectum cancer	Female	Africa	32,660 (13,752 to 51,390)	87.4(36.68 to 137.55)	1.64(1.48 to 1.8)
Gallbladder and biliary tract cancer	Female	Global	182,382 (120,376 to 258,795)	31.05(20.5 to 44.05)	-0.64(-0.74 to -0.54)
Gallbladder and biliary tract cancer	Female	High SDI	51,514 (33,046 to 72,864)	32.52(21.15 to 45.82)	-1.63(-1.8 to -1.45)
Gallbladder and biliary tract cancer	Female	High-middle SDI	52,391 (34,203 to 74,623)	36.68(23.97 to 52.24)	-0.95(-1.08 to -0.82)
Gallbladder and biliary tract cancer	Female	Middle SDI	49,729 (32,246 to 71,614)	28.28(18.32 to 40.73)	0.19(0.08 to 0.3)
Gallbladder and biliary tract cancer	Female	Low-middle SDI	24,519 (15,331 to 35,847)	26.97(16.89 to 39.45)	1.43(1.29 to 1.58)

Cause	Sex	Location	DALYs (95%CI)	ASDR (per 100,000 population, 95%CI)	AAPC (1990 – 2021, 95%CI)
Gallbladder and biliary tract cancer	Female	Low SDI	4,002 (2,276 to 6,094)	13.49(7.67 to 20.57)	1.27(1.13 to 1.4)
Gallbladder and biliary tract cancer	Female	High-income Asia Pacific	14,861 (8,738 to 21,657)	37.1(22.65 to 53.59)	-2.31(-2.51 to -2.11)
Gallbladder and biliary tract cancer	Female	High-income North America	10,653 (6,996 to 14,582)	21.92(14.46 to 29.91)	-0.69(-0.8 to -0.58)
Gallbladder and biliary tract cancer	Female	Western Europe	20,308 (13,090 to 28,799)	28.97(18.91 to 40.87)	-2.18(-2.43 to -1.94)
Gallbladder and biliary tract cancer	Female	Australasia	999 (645 to 1,419)	25.76(16.77 to 36.43)	-1(-1.08 to -0.91)
Gallbladder and biliary tract cancer	Female	Andean Latin America	4,081 (2,381 to 6,463)	107.74(62.86 to 170.63)	-0.17(-0.8 to 0.46)
Gallbladder and biliary tract cancer	Female	Tropical Latin America	9,306 (6,012 to 13,052)	51.81(33.47 to 72.67)	-0.76(-1.02 to -0.5)
Gallbladder and biliary tract cancer	Female	Central Latin America	9,867 (6,495 to 13,997)	58.65(38.62 to 83.22)	-1.93(-2.27 to -1.6)
Gallbladder and biliary tract cancer	Female	Southern Latin America	7,799 (5,022 to 11,095)	123.77(79.89 to 175.84)	-1.88(-2.28 to -1.47)
Gallbladder and biliary tract cancer	Female	Caribbean	645 (425 to 911)	17.98(11.86 to 25.4)	-1.13(-1.43 to -0.82)
Gallbladder and biliary tract cancer	Female	Central Europe	11,227 (7,455 to 15,790)	63.74(42.33 to 89.56)	-1.96(-2.19 to -1.74)
Gallbladder and biliary tract cancer	Female	Eastern Europe	10,588 (6,876 to 14,816)	34.75(22.58 to 48.61)	0.15(-0.52 to 0.81)

Cause	Sex	Location	DALYs (95%CI)	ASDR (per 100,000 population, 95%CI)	AAPC (1990 – 2021, 95%CI)
Gallbladder and biliary tract cancer	Female	Central Asia	1,201 (807 to 1,700)	21.47(14.44 to 30.38)	-0.49(-1.34 to 0.37)
Gallbladder and biliary tract cancer	Female	North Africa and Middle East	10,235 (6,071 to 15,248)	40.21(23.79 to 60.02)	0.41(0.31 to 0.51)
Gallbladder and biliary tract cancer	Female	South Asia	24,417 (13,768 to 37,061)	26.17(14.77 to 39.78)	2.49(2.29 to 2.69)
Gallbladder and biliary tract cancer	Female	Southeast Asia	8,031 (4,619 to 12,810)	18.73(10.72 to 30)	1.17(1.07 to 1.26)
Gallbladder and biliary tract cancer	Female	East Asia	35,602 (19,571 to 55,676)	24.24(13.32 to 37.91)	0.85(0.72 to 0.98)
Gallbladder and biliary tract cancer	Female	Oceania	37 (21 to 58)	9.74(5.61 to 15.1)	-0.31(-0.52 to -0.1)
Gallbladder and biliary tract cancer	Female	Western Sub-Saharan Africa	131 (70 to 199)	1.21(0.64 to 1.83)	1.52(1.35 to 1.69)
Gallbladder and biliary tract cancer	Female	Eastern Sub-Saharan Africa	1,324 (783 to 2,045)	13.42(7.91 to 20.77)	0.6(0.54 to 0.66)
Gallbladder and biliary tract cancer	Female	Central Sub-Saharan Africa	174 (91 to 301)	5.2(2.71 to 9.14)	1.72(1.65 to 1.78)
Gallbladder and biliary tract cancer	Female	Southern Sub-Saharan Africa	895 (529 to 1,325)	22.03(12.95 to 32.65)	1.48(1.09 to 1.88)
Gallbladder and biliary tract cancer	Female	Four World Regions	182,084 (120,169 to 258,388)	31.06(20.51 to 44.06)	-0.64(-0.74 to -0.53)
Gallbladder and biliary tract cancer	Female	Asia	87,527 (51,670 to 128,658)	26.56(15.67 to 39.04)	0.55(0.46 to 0.64)

Cause	Sex	Location	DALYs (95%CI)	ASDR (per 100,000 population, 95%CI)	AAPC (1990 – 2021, 95%CI)
Gallbladder and biliary tract cancer	Female	America	42,257 (28,056 to 58,600)	44.13(29.34 to 61.15)	-1(-1.2 to -0.8)
Gallbladder and biliary tract cancer	Female	Europe	44,455 (29,332 to 62,019)	36.31(24.07 to 50.48)	-1.52(-1.76 to -1.28)
Gallbladder and biliary tract cancer	Female	Africa	7,845 (4,676 to 11,810)	20.98(12.47 to 31.63)	1.11(0.93 to 1.29)
Liver cancer	Female	Global	262,013 (106,638 to 438,104)	44.64(18.17 to 74.65)	1.92(1.78 to 2.06)
Liver cancer	Female	High SDI	74,300 (30,596 to 123,440)	49.67(20.6 to 82.28)	2.44(2.29 to 2.6)
Liver cancer	Female	High-middle SDI	65,946 (26,381 to 115,848)	46.28(18.53 to 81.35)	1.63(1.25 to 2.01)
Liver cancer	Female	Middle SDI	73,386 (29,947 to 125,778)	41.38(16.86 to 70.87)	2.35(2.19 to 2.51)
Liver cancer	Female	Low-middle SDI	37,937 (15,578 to 63,400)	41.02(16.88 to 68.54)	1.39(1.24 to 1.54)
Liver cancer	Female	Low SDI	10,233 (3,797 to 17,844)	33.71(12.49 to 58.88)	1.13(1.09 to 1.17)
Liver cancer	Female	High-income Asia Pacific	10,736 (3,942 to 19,891)	30.26(11.18 to 56.2)	0.04(-0.22 to 0.31)
Liver cancer	Female	High-income North America	29,942 (12,774 to 48,528)	62.72(26.83 to 101.36)	3.11(2.91 to 3.31)
Liver cancer	Female	Western Europe	32,436 (12,770 to 56,121)	48.25(19.04 to 83.33)	1.9(1.67 to 2.14)

Cause	Sex	Location	DALYs (95%CI)	ASDR (per 100,000 population, 95%CI)	AAPC (1990 – 2021, 95%CI)
Liver cancer	Female	Australasia	2,253 (889 to 3,978)	60.78(24.03 to 106.98)	4.59(3.98 to 5.19)
Liver cancer	Female	Andean Latin America	1,849 (735 to 3,467)	48.83(19.4 to 91.56)	1.23(0.55 to 1.91)
Liver cancer	Female	Tropical Latin America	4,761 (1,891 to 8,092)	26.51(10.52 to 45.05)	0.86(0.64 to 1.09)
Liver cancer	Female	Central Latin America	10,306 (4,329 to 17,586)	61.37(25.74 to 104.72)	0.49(0.29 to 0.7)
Liver cancer	Female	Southern Latin America	1,853 (758 to 3,246)	29.53(12.12 to 51.66)	3.4(2.47 to 4.33)
Liver cancer	Female	Caribbean	845 (335 to 1,460)	23.58(9.36 to 40.71)	0.54(-0.03 to 1.1)
Liver cancer	Female	Central Europe	7,068 (2,866 to 12,044)	40.79(16.57 to 69.49)	-0.18(-0.62 to 0.26)
Liver cancer	Female	Eastern Europe	11,017 (4,485 to 18,221)	36.31(14.79 to 60.03)	1.4(0.58 to 2.22)
Liver cancer	Female	Central Asia	6,427 (2,664 to 11,360)	113.04(46.74 to 199.76)	0.49(0.27 to 0.72)
Liver cancer	Female	North Africa and Middle East	26,170 (10,924 to 43,761)	98.57(41.18 to 164.9)	0.61(0.28 to 0.95)
Liver cancer	Female	South Asia	11,054 (4,107 to 19,679)	11.68(4.35 to 20.88)	4.65(4.49 to 4.8)
Liver cancer	Female	Southeast Asia	11,214 (4,204 to 20,938)	25.48(9.47 to 47.84)	2.62(2.41 to 2.83)

Cause	Sex	Location	DALYs (95%CI)	ASDR (per 100,000 population, 95%CI)	AAPC (1990 – 2021, 95%CI)
Liver cancer	Female	East Asia	73,117 (28,700 to 132,079)	49.33(19.35 to 89.17)	3.64(3.16 to 4.13)
Liver cancer	Female	Oceania	136 (53 to 246)	33.26(12.96 to 60.14)	0.04(-0.15 to 0.23)
Liver cancer	Female	Western Sub-Saharan Africa	10,424 (3,994 to 17,978)	89.91(34.54 to 155.05)	0.67(0.55 to 0.79)
Liver cancer	Female	Eastern Sub-Saharan Africa	4,074 (1,478 to 7,397)	39.9(14.52 to 72.91)	2.09(1.95 to 2.24)
Liver cancer	Female	Central Sub-Saharan Africa	1,554 (390 to 4,577)	45.51(11.2 to 134.29)	2.13(2.03 to 2.24)
Liver cancer	Female	Southern Sub-Saharan Africa	4,778 (1,990 to 8,286)	116.32(48.41 to 201.96)	1.71(1.39 to 2.03)
Liver cancer	Female	Four World Regions	261,618 (106,469 to 437,459)	44.66(18.17 to 74.68)	1.93(1.79 to 2.06)
Liver cancer	Female	Asia	120,298 (48,471 to 208,001)	36.03(14.51 to 62.26)	2.71(2.41 to 3.02)
Liver cancer	Female	America	49,350 (20,944 to 81,198)	51.62(21.93 to 84.83)	2.11(1.96 to 2.27)
Liver cancer	Female	Europe	54,327 (21,931 to 91,524)	44.96(18.2 to 75.54)	1.3(1.1 to 1.5)
Liver cancer	Female	Africa	37,643 (15,481 to 62,077)	95.81(39.57 to 157.98)	1.07(0.91 to 1.22)
Multiple myeloma	Female	Global	71,145 (-29,999 to 176,973)	12.12(-5.11 to 30.13)	0.75(0.67 to 0.83)

Cause	Sex	Location	DALYs (95%CI)	ASDR (per 100,000 population, 95%CI)	AAPC (1990 – 2021, 95%CI)
Multiple myeloma	Female	High SDI	32,880 (-13,993 to 83,028)	21.36(-9.2 to 53.68)	0.13(0 to 0.26)
Multiple myeloma	Female	High-middle SDI	19,368 (-8,536 to 48,074)	13.59(-6 to 33.71)	1.1(1.01 to 1.2)
Multiple myeloma	Female	Middle SDI	12,690 (-5,068 to 31,333)	7.14(-2.84 to 17.62)	3.02(2.93 to 3.12)
Multiple myeloma	Female	Low-middle SDI	5,017 (-1,895 to 13,324)	5.45(-2.05 to 14.5)	3.31(3.22 to 3.4)
Multiple myeloma	Female	Low SDI	1,082 (-334 to 2,938)	3.59(-1.1 to 9.75)	2.64(2.56 to 2.72)
Multiple myeloma	Female	High-income Asia Pacific	2,022 (-562 to 5,456)	5.64(-1.61 to 15.26)	-0.31(-0.67 to 0.05)
Multiple myeloma	Female	High-income North America	14,740 (-6,354 to 36,703)	30.19(-13.07 to 74.96)	-0.19(-0.47 to 0.09)
Multiple myeloma	Female	Western Europe	16,645 (-6,941 to 43,043)	23.98(-10.07 to 61.74)	0.39(0.25 to 0.53)
Multiple myeloma	Female	Australasia	1,146 (-514 to 2,999)	29.85(-13.44 to 77.87)	0.85(0.64 to 1.07)
Multiple myeloma	Female	Andean Latin America	573 (-249 to 1,616)	15.11(-6.55 to 42.61)	2.7(2.09 to 3.32)
Multiple myeloma	Female	Tropical Latin America	3,458 (-1,445 to 8,817)	19.25(-8.04 to 49.09)	1.75(1.26 to 2.25)
Multiple myeloma	Female	Central Latin America	2,661 (-1,171 to 6,808)	15.71(-6.91 to 40.19)	1.55(0.86 to 2.23)

Cause	Sex	Location	DALYs (95%CI)	ASDR (per 100,000 population, 95%CI)	AAPC (1990 – 2021, 95%CI)
Multiple myeloma	Female	Southern Latin America	1,423 (-617 to 3,600)	22.48(-9.8 to 56.7)	0.27(0.07 to 0.47)
Multiple myeloma	Female	Caribbean	800 (-349 to 2,045)	22.34(-9.78 to 57.08)	1.42(0.82 to 2.02)
Multiple myeloma	Female	Central Europe	4,132 (-1,852 to 10,528)	23.71(-10.7 to 60.35)	1.46(1.36 to 1.57)
Multiple myeloma	Female	Eastern Europe	5,984 (-2,837 to 14,650)	19.84(-9.4 to 48.47)	2.15(1.86 to 2.45)
Multiple myeloma	Female	Central Asia	324 (-150 to 817)	5.46(-2.52 to 13.79)	2.66(2.06 to 3.27)
Multiple myeloma	Female	North Africa and Middle East	3,880 (-1,927 to 10,484)	14.74(-7.26 to 39.89)	2.09(1.95 to 2.23)
Multiple myeloma	Female	South Asia	4,017 (-1,347 to 10,980)	4.29(-1.43 to 11.74)	4.1(3.97 to 4.23)
Multiple myeloma	Female	Southeast Asia	1,174 (-443 to 3,421)	2.63(-0.98 to 7.69)	3.74(3.58 to 3.9)
Multiple myeloma	Female	East Asia	5,506 (-1,882 to 14,788)	3.71(-1.27 to 9.97)	6.16(5.64 to 6.67)
Multiple myeloma	Female	Oceania	14 (-7 to 37)	3.47(-1.66 to 9.07)	1.59(1.34 to 1.84)
Multiple myeloma	Female	Western Sub-Saharan Africa	692 (-246 to 2,125)	5.99(-2.13 to 18.38)	3.8(3.63 to 3.96)
Multiple myeloma	Female	Eastern Sub-Saharan Africa	734 (-232 to 1,976)	7.24(-2.26 to 19.54)	3.06(3 to 3.13)

Cause	Sex	Location	DALYs (95%CI)	ASDR (per 100,000 population, 95%CI)	AAPC (1990 – 2021, 95%CI)
Multiple myeloma	Female	Central Sub-Saharan Africa	97 (-35 to 301)	2.79(-1 to 8.67)	3.04(2.99 to 3.1)
Multiple myeloma	Female	Southern Sub-Saharan Africa	1,121 (-524 to 2,969)	26.85(-12.51 to 71.12)	2.37(2.21 to 2.53)
Multiple myeloma	Female	Four World Regions	70,858 (-29,869 to 176,243)	12.09(-5.1 to 30.07)	0.75(0.67 to 0.83)
Multiple myeloma	Female	Asia	15,529 (-5,438 to 39,535)	4.67(-1.63 to 11.9)	3.08(2.85 to 3.32)
Multiple myeloma	Female	America	23,452 (-10,227 to 59,189)	24.38(-10.66 to 61.5)	0.18(-0.01 to 0.36)
Multiple myeloma	Female	Europe	28,140 (-12,395 to 71,448)	23.23(-10.33 to 58.75)	1.07(0.9 to 1.23)
Multiple myeloma	Female	Africa	3,738 (-1,544 to 9,725)	9.61(-3.93 to 25.01)	2.71(2.64 to 2.78)
Non-Hodgkin lymphoma	Female	Global	86,999 (28,324 to 150,832)	14.81(4.82 to 25.68)	0.5(0.4 to 0.61)
Non-Hodgkin lymphoma	Female	High SDI	36,564 (11,900 to 63,737)	23.36(7.65 to 40.55)	-0.33(-0.43 to -0.22)
Non-Hodgkin lymphoma	Female	High-middle SDI	21,526 (6,890 to 37,730)	15.08(4.82 to 26.42)	0.79(0.59 to 0.99)
Non-Hodgkin lymphoma	Female	Middle SDI	17,726 (5,755 to 30,656)	10.03(3.26 to 17.36)	1.52(1.39 to 1.65)
Non-Hodgkin lymphoma	Female	Low-middle SDI	8,385 (2,929 to 14,860)	9.16(3.2 to 16.23)	2.29(2.19 to 2.4)

**Cause**	**Sex**	**Location**	**DALYs (95%CI)**	**ASDR (per 100,000 population, 95%CI)**	**AAPC (1990 – 2021, 95%CI)**
Non-Hodgkin lymphoma	Female	Low SDI	2,695 (919 to 4,696)	9.07(3.1 to 15.78)	1.73(1.64 to 1.81)
Non-Hodgkin lymphoma	Female	High-income Asia Pacific	3,694 (1,136 to 6,598)	10(3.12 to 17.74)	0.11(-0.07 to 0.28)
Non-Hodgkin lymphoma	Female	High-income North America	16,844 (5,500 to 28,840)	34.11(11.15 to 58.27)	-0.94(-1.11 to -0.77)
Non-Hodgkin lymphoma	Female	Western Europe	15,649 (5,019 to 27,623)	22.22(7.19 to 39.1)	-0.14(-0.39 to 0.11)
Non-Hodgkin lymphoma	Female	Australasia	1,178 (360 to 2,113)	30.34(9.27 to 54.21)	-0.57(-0.95 to -0.19)
Non-Hodgkin lymphoma	Female	Andean Latin America	1,225 (388 to 2,285)	32.34(10.23 to 60.34)	1.97(1.47 to 2.47)
Non-Hodgkin lymphoma	Female	Tropical Latin America	2,967 (952 to 5,223)	16.53(5.3 to 29.09)	0.94(0.69 to 1.18)
Non-Hodgkin lymphoma	Female	Central Latin America	3,432 (1,145 to 5,999)	20.41(6.81 to 35.69)	1.59(1.36 to 1.81)
Non-Hodgkin lymphoma	Female	Southern Latin America	1,585 (508 to 2,828)	24.92(7.99 to 44.45)	0.18(-0.18 to 0.54)
Non-Hodgkin lymphoma	Female	Caribbean	655 (213 to 1,150)	18.29(5.96 to 32.13)	0.63(0.28 to 0.98)
Non-Hodgkin lymphoma	Female	Central Europe	3,644 (1,175 to 6,365)	20.84(6.71 to 36.4)	1.35(1.18 to 1.52)
Non-Hodgkin lymphoma	Female	Eastern Europe	5,625 (1,809 to 9,721)	18.53(5.95 to 32.03)	2.26(1.56 to 2.98)

Cause	Sex	Location	DALYs (95%CI)	ASDR (per 100,000 population, 95%CI)	AAPC (1990 – 2021, 95%CI)
Non-Hodgkin lymphoma	Female	Central Asia	435 (139 to 760)	7.55(2.41 to 13.17)	0.32(-0.51 to 1.16)
Non-Hodgkin lymphoma	Female	North Africa and Middle East	5,885 (1,907 to 10,620)	22.76(7.37 to 41.08)	1.53(1.37 to 1.7)
Non-Hodgkin lymphoma	Female	South Asia	5,738 (1,953 to 10,138)	6.15(2.1 to 10.88)	2.58(2.45 to 2.72)
Non-Hodgkin lymphoma	Female	Southeast Asia	3,191 (1,088 to 5,752)	7.22(2.46 to 13.02)	1.95(1.83 to 2.07)
Non-Hodgkin lymphoma	Female	East Asia	10,938 (3,267 to 19,784)	7.42(2.22 to 13.43)	1.01(0.64 to 1.38)
Non-Hodgkin lymphoma	Female	Oceania	38 (13 to 68)	9.76(3.19 to 17.4)	0.94(0.81 to 1.08)
Non-Hodgkin lymphoma	Female	Western Sub-Saharan Africa	1,429 (417 to 2,561)	12.46(3.65 to 22.32)	1.79(1.72 to 1.86)
Non-Hodgkin lymphoma	Female	Eastern Sub-Saharan Africa	1,609 (528 to 2,903)	16.18(5.33 to 29.19)	1.86(1.78 to 1.94)
Non-Hodgkin lymphoma	Female	Central Sub-Saharan Africa	283 (80 to 622)	8.34(2.36 to 18.49)	2.19(2.1 to 2.28)
Non-Hodgkin lymphoma	Female	Southern Sub-Saharan Africa	953 (296 to 1,708)	23.27(7.21 to 41.7)	2.24(1.53 to 2.96)
Non-Hodgkin lymphoma	Female	Four World Regions	86,748 (28,240 to 150,403)	14.8(4.82 to 25.65)	0.5(0.4 to 0.61)
Non-Hodgkin lymphoma	Female	Asia	27,397 (8,886 to 47,478)	8.26(2.67 to 14.32)	1.44(1.34 to 1.54)

Cause	Sex	Location	DALYs (95%CI)	ASDR (per 100,000 population, 95%CI)	AAPC (1990 – 2021, 95%CI)
Non-Hodgkin lymphoma	Female	America	26,539 (8,733 to 45,591)	27.43(9.03 to 47.07)	-0.34(-0.5 to -0.19)
Non-Hodgkin lymphoma	Female	Europe	26,389 (8,555 to 46,369)	21.49(6.98 to 37.69)	0.68(0.48 to 0.88)
Non-Hodgkin lymphoma	Female	Africa	6,423 (2,064 to 11,262)	16.71(5.38 to 29.29)	1.95(1.73 to 2.16)
Leukemia	Female	Global	156,356 (111,267 to 205,661)	26.62(18.95 to 35)	-0.31(-0.36 to -0.26)
Leukemia	Female	High SDI	58,690 (40,968 to 76,414)	37.1(26.27 to 48.01)	-0.69(-0.76 to -0.63)
Leukemia	Female	High-middle SDI	44,821 (31,709 to 59,396)	31.39(22.23 to 41.59)	-0.5(-0.6 to -0.41)
Leukemia	Female	Middle SDI	34,714 (23,947 to 47,143)	19.69(13.56 to 26.74)	0.11(0.03 to 0.19)
Leukemia	Female	Low-middle SDI	14,545 (10,186 to 19,992)	16.01(11.18 to 22.03)	0.99(0.92 to 1.06)
Leukemia	Female	Low SDI	3,373 (2,175 to 4,731)	11.49(7.36 to 16.14)	0.22(0.18 to 0.26)
Leukemia	Female	High-income Asia Pacific	4,676 (2,890 to 6,537)	13.33(8.53 to 18.46)	-1.57(-1.7 to -1.43)
Leukemia	Female	High-income North America	25,382 (17,929 to 32,658)	51.04(36.33 to 65.5)	-0.7(-0.8 to -0.6)
Leukemia	Female	Western Europe	28,929 (19,575 to 38,690)	39.98(27.6 to 52.99)	-0.76(-0.98 to -0.53)

Cause	Sex	Location	DALYs (95%CI)	ASDR (per 100,000 population, 95%CI)	AAPC (1990 – 2021, 95%CI)
Leukemia	Female	Australasia	1,705 (1,121 to 2,361)	43.31(28.79 to 59.74)	-0.5(-1.02 to 0.03)
Leukemia	Female	Andean Latin America	1,479 (929 to 2,182)	39.04(24.53 to 57.56)	0.92(0.19 to 1.66)
Leukemia	Female	Tropical Latin America	5,496 (3,833 to 7,262)	30.61(21.35 to 40.44)	0.14(-0.07 to 0.35)
Leukemia	Female	Central Latin America	5,427 (3,959 to 7,160)	32.24(23.51 to 42.56)	0.67(0.56 to 0.78)
Leukemia	Female	Southern Latin America	2,499 (1,725 to 3,407)	38.92(27 to 52.91)	-0.23(-0.5 to 0.05)
Leukemia	Female	Caribbean	1,031 (731 to 1,393)	28.58(20.31 to 38.6)	0.3(-0.1 to 0.7)
Leukemia	Female	Central Europe	8,468 (6,063 to 10,975)	47.81(34.29 to 61.88)	-0.3(-0.48 to -0.11)
Leukemia	Female	Eastern Europe	12,667 (9,206 to 16,568)	41.79(30.46 to 54.65)	-0.27(-0.6 to 0.07)
Leukemia	Female	Central Asia	1,240 (902 to 1,668)	21.46(15.62 to 28.8)	-0.69(-1.11 to -0.27)
Leukemia	Female	North Africa and Middle East	12,785 (8,195 to 17,693)	49.72(31.83 to 69.02)	0.22(0.09 to 0.35)
Leukemia	Female	South Asia	8,831 (5,759 to 13,017)	9.56(6.21 to 14.11)	0.98(0.85 to 1.11)
Leukemia	Female	Southeast Asia	8,101 (5,384 to 11,499)	18.6(12.3 to 26.53)	0.7(0.54 to 0.86)

Cause	Sex	Location	DALYs (95%CI)	ASDR (per 100,000 population, 95%CI)	AAPC (1990 – 2021, 95%CI)
Leukemia	Female	East Asia	23,887 (14,318 to 34,921)	16.22(9.7 to 23.73)	-0.29(-0.5 to -0.07)
Leukemia	Female	Oceania	83 (49 to 125)	21.33(12.5 to 31.86)	-0.22(-0.35 to -0.09)
Leukemia	Female	Western Sub-Saharan Africa	658 (394 to 934)	5.87(3.52 to 8.34)	0.81(0.7 to 0.92)
Leukemia	Female	Eastern Sub-Saharan Africa	1,366 (864 to 2,006)	14.01(8.79 to 20.6)	-0.16(-0.23 to -0.09)
Leukemia	Female	Central Sub-Saharan Africa	339 (185 to 554)	10.17(5.42 to 16.97)	1.41(1.36 to 1.46)
Leukemia	Female	Southern Sub-Saharan Africa	1,307 (797 to 1,809)	32.63(19.71 to 45.29)	0.89(0.58 to 1.2)
Leukemia	Female	Four World Regions	155,930 (110,951 to 205,136)	26.6(18.93 to 34.98)	-0.31(-0.36 to -0.26)
Leukemia	Female	Asia	53,597 (35,473 to 74,352)	16.11(10.65 to 22.35)	0.01(-0.15 to 0.17)
Leukemia	Female	America	41,069 (29,368 to 52,631)	42.31(30.37 to 54.14)	-0.21(-0.25 to -0.16)
Leukemia	Female	Europe	53,205 (37,720 to 69,547)	42.9(30.74 to 55.82)	-0.5(-0.66 to -0.33)
Leukemia	Female	Africa	8,059 (5,149 to 11,117)	21.17(13.51 to 29.22)	0.89(0.74 to 1.04)
Kidney cancer	Female	Global	183,052 (73,395 to 292,970)	31.15(12.49 to 49.86)	0.04(-0.09 to 0.16)

Cause	Sex	Location	DALYs (95%CI)	ASDR (per 100,000 population, 95%CI)	AAPC (1990 – 2021, 95%CI)
Kidney cancer	Female	High SDI	79,024 (31,504 to 127,348)	51.12(20.57 to 81.97)	-0.22(-0.35 to -0.09)
Kidney cancer	Female	High-middle SDI	62,954 (25,508 to 99,759)	44.05(17.86 to 69.76)	0.15(-0.14 to 0.43)
Kidney cancer	Female	Middle SDI	29,415 (11,909 to 47,901)	16.68(6.74 to 27.19)	1.86(1.77 to 1.95)
Kidney cancer	Female	Low-middle SDI	9,344 (3,626 to 15,050)	10.25(3.97 to 16.54)	2.33(2.13 to 2.53)
Kidney cancer	Female	Low SDI	2,011 (735 to 3,475)	6.72(2.45 to 11.62)	1.7(1.63 to 1.77)
Kidney cancer	Female	High-income Asia Pacific	5,328 (1,929 to 9,002)	14.05(5.14 to 23.57)	0.62(0.35 to 0.88)
Kidney cancer	Female	High-income North America	30,448 (12,457 to 47,405)	62.4(25.62 to 96.8)	-0.38(-0.53 to -0.24)
Kidney cancer	Female	Western Europe	37,848 (14,725 to 61,951)	54.33(21.35 to 88.38)	-0.17(-0.28 to -0.06)
Kidney cancer	Female	Australasia	1,910 (758 to 3,161)	49.58(19.72 to 81.69)	-0.63(-1.03 to -0.24)
Kidney cancer	Female	Andean Latin America	1,641 (667 to 2,914)	43.3(17.61 to 76.91)	0.42(-0.26 to 1.1)
Kidney cancer	Female	Tropical Latin America	6,674 (2,651 to 10,907)	37.12(14.74 to 60.66)	1.65(1.18 to 2.12)
Kidney cancer	Female	Central Latin America	8,829 (3,711 to 14,212)	52.32(21.97 to 84.27)	1.02(0.82 to 1.21)

Cause	Sex	Location	DALYs (95%CI)	ASDR (per 100,000 population, 95%CI)	AAPC (1990 – 2021, 95%CI)
Kidney cancer	Female	Southern Latin America	5,378 (2,120 to 8,698)	85.45(33.74 to 137.93)	0.72(0.43 to 1.01)
Kidney cancer	Female	Caribbean	979 (388 to 1,624)	27.31(10.82 to 45.25)	1.06(0.96 to 1.16)
Kidney cancer	Female	Central Europe	15,351 (6,395 to 24,883)	87.21(36.5 to 141.16)	0.79(0.64 to 0.93)
Kidney cancer	Female	Eastern Europe	26,812 (10,875 to 41,910)	87.9(35.68 to 137.36)	0.62(0.07 to 1.16)
Kidney cancer	Female	Central Asia	2,448 (1,013 to 3,936)	42.55(17.58 to 68.56)	0.59(-0.04 to 1.22)
Kidney cancer	Female	North Africa and Middle East	6,763 (2,769 to 10,749)	26.41(10.75 to 42.19)	1.39(1.21 to 1.57)
Kidney cancer	Female	South Asia	4,711 (1,696 to 8,166)	5.03(1.82 to 8.75)	3.91(3.71 to 4.11)
Kidney cancer	Female	Southeast Asia	3,488 (1,288 to 6,046)	7.99(2.94 to 13.89)	2.71(2.59 to 2.82)
Kidney cancer	Female	East Asia	20,821 (8,077 to 36,624)	14.17(5.49 to 24.93)	2.69(2.39 to 3)
Kidney cancer	Female	Oceania	8 (3 to 13)	2.18(0.87 to 3.7)	0.92(0.39 to 1.46)
Kidney cancer	Female	Western Sub-Saharan Africa	1,015 (358 to 1,745)	8.66(3.06 to 14.9)	2.62(2.57 to 2.67)
Kidney cancer	Female	Eastern Sub-Saharan Africa	1,361 (482 to 2,475)	13.57(4.79 to 24.6)	2.08(2 to 2.17)

**Cause**	**Sex**	**Location**	**DALYs (95%CI)**	**ASDR (per 100,000 population, 95%CI)**	**AAPC (1990 – 2021, 95%CI)**
Kidney cancer	Female	Central Sub-Saharan Africa	166 (53 to 350)	4.91(1.55 to 10.47)	2.02(1.93 to 2.11)
Kidney cancer	Female	Southern Sub-Saharan Africa	1,073 (428 to 1,708)	26.38(10.51 to 42.11)	1.87(1.54 to 2.19)
Kidney cancer	Female	Four World Regions	182,562 (73,190 to 292,216)	31.13(12.48 to 49.83)	0.04(-0.09 to 0.16)
Kidney cancer	Female	Asia	40,763 (15,837 to 68,583)	12.33(4.78 to 20.76)	1.95(1.79 to 2.12)
Kidney cancer	Female	America	53,726 (22,050 to 84,871)	55.85(22.97 to 88.14)	0.11(-0.1 to 0.32)
Kidney cancer	Female	Europe	82,667 (33,261 to 132,689)	68.03(27.53 to 108.82)	0.24(0.11 to 0.36)
Kidney cancer	Female	Africa	5,406 (2,101 to 8,822)	14.05(5.44 to 22.95)	2.15(2.02 to 2.28)
Thyroid cancer	Female	Global	48,334 (35,010 to 62,679)	8.24(5.97 to 10.68)	0.14(0.07 to 0.21)
Thyroid cancer	Female	High SDI	12,665 (8,865 to 16,366)	8.15(5.8 to 10.45)	-0.48(-0.62 to -0.34)
Thyroid cancer	Female	High-middle SDI	11,819 (8,526 to 15,392)	8.27(5.97 to 10.76)	-0.73(-0.88 to -0.59)
Thyroid cancer	Female	Middle SDI	14,779 (10,691 to 19,564)	8.4(6.06 to 11.13)	0.81(0.74 to 0.88)
Thyroid cancer	Female	Low-middle SDI	7,043 (4,971 to 9,349)	7.76(5.47 to 10.32)	1.42(1.36 to 1.48)

Cause	Sex	Location	DALYs (95%CI)	ASDR (per 100,000 population, 95%CI)	AAPC (1990 – 2021, 95%CI)
Thyroid cancer	Female	Low SDI	1,971 (1,312 to 2,853)	6.68(4.43 to 9.7)	0.57(0.52 to 0.61)
Thyroid cancer	Female	High-income Asia Pacific	2,712 (1,661 to 3,834)	7.16(4.56 to 10.09)	-0.59(-0.94 to -0.23)
Thyroid cancer	Female	High-income North America	4,315 (3,132 to 5,464)	8.85(6.46 to 11.19)	0.73(0.56 to 0.91)
Thyroid cancer	Female	Western Europe	5,006 (3,419 to 6,546)	7.31(5.1 to 9.49)	-1.58(-1.85 to -1.31)
Thyroid cancer	Female	Australasia	270 (179 to 373)	7.12(4.74 to 9.82)	0.03(-0.94 to 1.01)
Thyroid cancer	Female	Andean Latin America	1,049 (684 to 1,513)	27.72(18.07 to 39.97)	1.04(0.35 to 1.74)
Thyroid cancer	Female	Tropical Latin America	1,717 (1,222 to 2,245)	9.57(6.81 to 12.52)	-0.45(-0.69 to -0.22)
Thyroid cancer	Female	Central Latin America	3,409 (2,535 to 4,432)	20.29(15.08 to 26.39)	0.51(-0.07 to 1.1)
Thyroid cancer	Female	Southern Latin America	762 (540 to 1,011)	11.94(8.48 to 15.83)	-0.85(-1.39 to -0.31)
Thyroid cancer	Female	Caribbean	388 (277 to 521)	10.8(7.73 to 14.49)	0.66(0.09 to 1.23)
Thyroid cancer	Female	Central Europe	1,839 (1,360 to 2,350)	10.52(7.81 to 13.43)	-1.56(-2.19 to -0.92)
Thyroid cancer	Female	Eastern Europe	3,341 (2,489 to 4,273)	10.93(8.17 to 13.99)	0.2(-0.66 to 1.07)

Cause	Sex	Location	DALYs (95%CI)	ASDR (per 100,000 population, 95%CI)	AAPC (1990 – 2021, 95%CI)
Thyroid cancer	Female	Central Asia	586 (432 to 756)	10.38(7.65 to 13.41)	-0.4(-1.76 to 0.98)
Thyroid cancer	Female	North Africa and Middle East	2,970 (2,063 to 4,178)	11.61(8.04 to 16.35)	0.86(0.75 to 0.97)
Thyroid cancer	Female	South Asia	5,814 (3,780 to 8,106)	6.28(4.07 to 8.78)	1.97(1.78 to 2.16)
Thyroid cancer	Female	Southeast Asia	5,058 (3,378 to 7,025)	11.62(7.73 to 16.21)	1.23(1.13 to 1.33)
Thyroid cancer	Female	East Asia	7,221 (4,558 to 10,684)	4.92(3.1 to 7.28)	-0.32(-0.47 to -0.16)
Thyroid cancer	Female	Oceania	47 (27 to 71)	12.4(7.32 to 18.8)	0.19(0.11 to 0.26)
Thyroid cancer	Female	Western Sub-Saharan Africa	176 (116 to 251)	1.55(1.02 to 2.21)	-0.08(-0.23 to 0.06)
Thyroid cancer	Female	Eastern Sub-Saharan Africa	1,017 (650 to 1,607)	10.31(6.57 to 16.24)	0.1(0.03 to 0.18)
Thyroid cancer	Female	Central Sub-Saharan Africa	173 (86 to 322)	5.2(2.55 to 9.77)	0.74(0.69 to 0.8)
Thyroid cancer	Female	Southern Sub-Saharan Africa	466 (317 to 633)	11.44(7.74 to 15.58)	1.33(1.06 to 1.6)
Thyroid cancer	Female	Four World Regions	48,236 (34,937 to 62,557)	8.23(5.97 to 10.68)	0.14(0.07 to 0.22)
Thyroid cancer	Female	Asia	22,656 (15,407 to 30,659)	6.84(4.64 to 9.27)	0.76(0.71 to 0.82)

Cause	Sex	Location	DALYs (95%CI)	ASDR (per 100,000 population, 95%CI)	AAPC (1990 – 2021, 95%CI)
Thyroid cancer	Female	America	11,586 (8,607 to 14,661)	12.07(8.99 to 15.25)	0.53(0.43 to 0.63)
Thyroid cancer	Female	Europe	11,142 (8,094 to 14,133)	9.16(6.72 to 11.58)	-0.95(-1.28 to -0.62)
Thyroid cancer	Female	Africa	2,851 (1,953 to 4,017)	7.5(5.12 to 10.56)	0.55(0.4 to 0.7)

Table S9. Number and age-specific rates of DALYs for total cancer attributable to high BMI among older people by age groups in 2021, with average annual percent change from 1990 to 2021.

Sex	Age	DALYs in 1990 (95%UI)	DALYs in 2021 (95%UI)	Age special DALYs rate in 1990 (per 100,000 population,95%UI)	Age special DALYs rate in 2021 (per 100,000 population, 95%UI)	AAPC of age special DALYs rate (95%CI)
Both	60 to 64	585,316 (236,528 to 964,786)	1,353,072 (523,619 to 2,202,283)	364.43(147.27 to 600.7)	422.77(163.61 to 688.11)	0.48(0.31 to 0.66)
Both	65 to 69	544,755 (219,427 to 892,149)	1,339,697 (547,732 to 2,170,152)	440.71(177.52 to 721.75)	485.68(198.57 to 786.74)	0.31(0.19 to 0.43)
Both	70 to 74	382,766 (155,754 to 626,851)	1,095,300 (446,459 to 1,787,368)	452.11(183.97 to 740.42)	532.11(216.9 to 868.33)	0.53(0.39 to 0.67)
Both	75 to 79	318,749 (130,642 to 523,875)	714,942 (290,565 to 1,167,970)	517.82(212.24 to 851.06)	542.1(220.32 to 885.6)	0.12(0.03 to 0.22)
Both	80 to 84	167,968 (67,272 to 277,442)	458,725 (178,518 to 756,619)	474.81(190.16 to 784.27)	523.76(203.83 to 863.89)	0.32(0.24 to 0.4)
Both	85 to 89	75,106 (29,137 to 126,121)	266,173 (101,022 to 446,525)	497.03(192.82 to 834.63)	582.16(220.95 to 976.61)	0.52(0.39 to 0.64)
Both	90 to 94	25,233 (9,551 to 42,553)	127,589 (47,319 to 217,821)	588.84(222.89 to 993.03)	713.21(264.51 to 1217.6)	0.63(0.52 to 0.74)
Both	95 plus	6,413 (2,334 to 10,969)	43,223 (15,569 to 74,145)	629.94(229.26 to 1077.43)	793.04(285.65 to 1360.39)	0.76(0.56 to 0.96)
Male	60 to 64	201,446 (90,677 to 331,619)	520,444 (225,101 to 839,389)	256.47(115.44 to 422.2)	334.61(144.73 to 539.67)	0.86(0.75 to 0.98)
Male	65 to 69	181,061 (80,396 to 296,633)	516,430 (221,797 to 844,499)	315.82(140.23 to 517.4)	391.73(168.24 to 640.58)	0.71(0.55 to 0.87)
Male	70 to 74	123,300 (55,947 to 201,668)	421,907 (180,715 to 689,423)	327.76(148.72 to 536.09)	437.7(187.48 to 715.23)	0.95(0.8 to 1.09)
Male	75 to 79	96,481 (44,093 to 154,295)	276,246 (119,866 to 444,339)	382.36(174.75 to 611.49)	462.05(200.49 to 743.21)	0.59(0.44 to 0.74)
Male	80 to 84	47,720 (22,207 to 75,835)	169,805 (73,750 to 277,476)	359.25(167.18 to 570.9)	463.29(201.22 to 757.06)	0.83(0.74 to 0.93)
Male	85 to 89	18,713 (8,666 to 30,199)	93,536 (40,763 to 151,940)	369.53(171.13 to 596.33)	542.15(236.27 to 880.67)	1.26(1.08 to 1.43)
Male	90 to 94	5,104 (2,382 to 8,237)	36,630 (16,284 to 59,479)	405.45(189.21 to 654.24)	628.46(279.39 to 1020.48)	1.44(1.34 to 1.55)
Male	95 plus	1,012 (467 to 1,658)	8,911 (3,899 to 14,547)	389.07(179.41 to 637.29)	589.34(257.87 to 962.11)	1.33(1.15 to 1.51)
Female	60 to 64	383,870 (145,851 to 637,850)	832,627 (297,493 to 1,360,763)	467.77(177.73 to 777.27)	506.12(180.83 to 827.16)	0.26(0.07 to 0.45)
Female	65 to 69	363,694 (139,015 to 605,131)	823,267 (320,735 to 1,338,065)	548.74(209.74 to 913.02)	571.68(222.72 to 929.15)	0.11(-0.02 to 0.24)
Female	70 to 74	259,466 (101,593 to 428,032)	673,392 (262,553 to 1,112,733)	551.55(215.96 to 909.88)	615.26(239.89 to 1016.68)	0.35(0.23 to 0.48)

Sex	Age	DALYs in 1990 (95%UI)	DALYs in 2021 (95%UI)	Age special DALYs rate in 1990 (per 100,000 population,95%UI)	Age special DALYs rate in 2021 (per 100,000 population, 95%UI)	AAPC of age special DALYs rate (95%CI)
Female	75 to 79	222,268 (86,870 to 368,224)	438,696 (168,277 to 728,865)	611.93(239.16 to 1013.76)	608.47(233.4 to 1010.94)	-0.03(-0.18 to 0.12)
Female	80 to 84	120,248 (45,095 to 200,977)	288,920 (104,768 to 486,661)	544.29(204.12 to 909.71)	567.27(205.71 to 955.53)	0.13(0.06 to 0.2)
Female	85 to 89	56,393 (20,766 to 95,802)	172,637 (60,864 to 296,506)	561.29(206.69 to 953.53)	606.4(213.79 to 1041.5)	0.27(0.14 to 0.4)
Female	90 to 94	20,128 (7,255 to 34,363)	90,959 (31,642 to 157,963)	665.13(239.72 to 1135.5)	754.17(262.35 to 1309.71)	0.4(0.29 to 0.51)
Female	95 plus	5,401 (1,886 to 9,364)	34,312 (11,674 to 59,644)	712.64(248.8 to 1235.56)	871.24(296.41 to 1514.47)	0.67(0.43 to 0.91)

Table S10. Proportion of DALYs for total cancer attributable to high body mass index from 1990 to 2021 by age groups and regions.

Location	Sex	60 to 64	65 to 69	70 to 74	75 to 79	80 to 84	85 to 89	90 to 94	95 plus
Global	Both	25.06%	24.82%	20.29%	13.24%	8.50%	4.93%	2.36%	0.80%
Asia	Both	27.45%	27.14%	20.11%	12.35%	6.77%	3.93%	1.70%	0.56%
America	Both	25.27%	24.09%	20.47%	13.97%	8.01%	4.84%	2.44%	0.92%
Europe	Both	21.13%	22.65%	20.66%	14.01%	10.96%	6.35%	3.19%	1.04%
Africa	Both	34.96%	28.66%	18.17%	10.14%	5.15%	2.21%	0.58%	0.12%
High SDI	Both	20.03%	21.43%	20.68%	15.27%	10.41%	6.92%	3.80%	1.45%
High-middle SDI	Both	25.13%	25.84%	20.61%	12.47%	8.84%	4.61%	1.97%	0.53%
Middle SDI	Both	29.86%	27.78%	19.66%	11.96%	6.22%	3.14%	1.10%	0.28%
Low-middle SDI	Both	33.88%	28.56%	19.23%	10.56%	4.91%	2.00%	0.67%	0.19%
Low SDI	Both	35.98%	28.87%	18.55%	9.90%	4.50%	1.64%	0.46%	0.10%
High-income Asia Pacific	Both	15.13%	17.33%	19.84%	16.28%	12.24%	10.03%	6.34%	2.81%
High-income North America	Both	23.02%	23.06%	21.17%	14.67%	8.51%	5.45%	2.96%	1.18%
Western Europe	Both	16.39%	18.19%	19.77%	16.72%	13.22%	9.04%	4.94%	1.74%
Australasia	Both	19.21%	20.28%	20.57%	16.13%	10.76%	7.37%	4.19%	1.48%
Andean Latin America	Both	28.89%	26.01%	19.80%	12.68%	7.03%	3.64%	1.51%	0.45%
Tropical Latin America	Both	28.88%	25.94%	19.62%	12.43%	7.14%	3.76%	1.68%	0.55%
Central Latin America	Both	31.19%	26.20%	18.61%	12.32%	6.60%	3.38%	1.30%	0.39%
Southern Latin America	Both	24.05%	24.27%	20.49%	14.64%	8.50%	5.15%	2.21%	0.70%
Caribbean	Both	27.63%	24.75%	19.23%	13.66%	7.62%	4.26%	2.01%	0.86%
Central Europe	Both	21.00%	25.93%	22.31%	14.19%	9.19%	5.05%	1.84%	0.49%
Eastern Europe	Both	28.36%	27.85%	21.46%	9.19%	8.80%	2.81%	1.27%	0.25%
Central Asia	Both	39.06%	29.46%	16.64%	7.47%	5.42%	1.34%	0.49%	0.11%

Location	Sex	60 to 64	65 to 69	70 to 74	75 to 79	80 to 84	85 to 89	90 to 94	95 plus
North Africa and Middle East	Both	32.25%	27.55%	19.14%	11.09%	5.84%	2.96%	0.90%	0.26%
South Asia	Both	33.52%	29.17%	19.91%	10.43%	4.45%	1.82%	0.59%	0.12%
Southeast Asia	Both	37.25%	28.36%	17.73%	9.62%	4.22%	1.89%	0.73%	0.21%
East Asia	Both	25.60%	29.03%	21.07%	12.75%	6.62%	3.57%	1.13%	0.23%
Oceania	Both	43.18%	28.72%	15.44%	7.26%	3.56%	1.41%	0.34%	0.09%
Western Sub-Saharan Africa	Both	35.77%	27.74%	17.46%	10.83%	5.33%	2.03%	0.67%	0.16%
Eastern Sub-Saharan Africa	Both	35.75%	28.55%	18.81%	9.57%	4.95%	1.77%	0.48%	0.13%
Central Sub-Saharan Africa	Both	39.82%	32.23%	14.74%	8.18%	3.37%	1.26%	0.34%	0.07%
Southern Sub-Saharan Africa	Both	31.21%	28.10%	18.69%	11.16%	6.67%	3.13%	0.93%	0.11%

Table S11. Proportion of DALYs attributable to high body mass index from 1990 to 2021 by sex, cancer subtypes, and age groups.

Location	Sex	Age groups	Breast cancer	Uterine cancer	Ovarian cancer	Colon and rectum cancer	Liver cancer	Kidney cancer	Leukemia	Gallbladder and biliary tract cancer	Non-Hodgkin lymphoma	Multiple myeloma	Thyroid cancer
Global	Male	60 to 64	NA	NA	NA	36.60%	24.78%	16.14%	8.06%	5.31%	4.58%	3.19%	1.35%
Global	Male	65 to 69	NA	NA	NA	38.19%	22.36%	15.54%	8.76%	5.66%	4.72%	3.46%	1.31%
Global	Male	70 to 74	NA	NA	NA	39.70%	17.98%	15.64%	10.00%	6.22%	5.17%	3.97%	1.33%
Global	Male	75 to 79	NA	NA	NA	40.33%	15.25%	15.33%	11.07%	6.79%	5.26%	4.19%	1.80%
Global	Male	80 to 84	NA	NA	NA	41.92%	13.27%	15.35%	11.60%	6.88%	5.21%	4.17%	1.62%
Global	Male	85 to 89	NA	NA	NA	43.45%	10.95%	15.54%	11.95%	7.22%	5.23%	4.17%	1.50%
Global	Male	90 to 94	NA	NA	NA	44.78%	9.23%	15.51%	12.88%	6.97%	5.33%	3.88%	1.42%
Global	Male	95 plus	NA	NA	NA	47.04%	7.57%	14.25%	13.54%	6.76%	5.55%	3.96%	1.32%
Global	Female	60 to 64	27.37%	18.80%	8.87%	17.72%	7.99%	4.74%	4.10%	4.91%	2.25%	1.86%	1.38%
Global	Female	65 to 69	23.70%	18.98%	8.12%	19.36%	8.98%	5.25%	4.36%	5.31%	2.44%	2.05%	1.44%
Global	Female	70 to 74	21.75%	17.87%	7.44%	21.73%	8.13%	5.77%	4.89%	5.75%	2.80%	2.34%	1.53%
Global	Female	75 to 79	20.48%	15.59%	6.56%	24.34%	8.19%	6.10%	5.34%	6.17%	3.07%	2.57%	1.60%
Global	Female	80 to 84	20.43%	14.33%	5.42%	27.09%	7.00%	6.68%	5.66%	6.17%	3.12%	2.48%	1.62%
Global	Female	85 to 89	20.96%	11.89%	4.58%	29.61%	6.59%	6.98%	5.92%	6.48%	3.12%	2.37%	1.51%
Global	Female	90 to 94	21.58%	10.89%	3.89%	32.14%	5.49%	6.88%	6.14%	6.33%	3.10%	2.15%	1.41%
Global	Female	95 plus	23.74%	9.71%	3.22%	33.49%	4.72%	6.21%	6.22%	6.46%	3.06%	1.75%	1.41%

Table S12. Decomposition analysis of DALYs for total cancer attributable to high BMI among older people by region, 1990 to 2021.

Location		Overall difference	Aging	Population	Epidemiological change
Global		3292413.45(156.31%)	46956.67(2.23%)	2802276.34(133.04%)	443180.44(21.04%)
	Male	1369071.7(202.87%)	25049.9(3.71%)	1019739.42(151.11%)	324282.39(48.05%)
	Female	1923341.75(134.36%)	17714.47(1.24%)	1759677.11(122.93%)	145950.18(10.20%)
Asia		1354887.41(391.46%)	6795.15(1.96%)	813951.63(235.17%)	534140.62(154.33%)
America		853878.54(145.87%)	-346.7(-0.06%)	771304.89(131.76%)	82920.35(14.17%)
Europe		817149.65(74.32%)	15091.16(1.37%)	612998.78(55.75%)	189059.71(17.19%)
Africa		257610.04(376.82%)	-428.44(-0.63%)	133796.61(195.71%)	124241.87(181.73%)
Low-middle SDI		365382.93(435.33%)	-1328.13(-1.58%)	194970.24(232.29%)	171740.82(204.62%)
Low SDI		75471.8(263.42%)	-472.85(-1.65%)	45950.17(160.38%)	29994.47(104.69%)
High-middle SDI		956843.08(140.31%)	7347.14(1.08%)	769236.72(112.8%)	180259.22(26.43%)
High SDI		993306.35(90.68%)	18858.37(1.72%)	979722.7(89.44%)	-5274.71(-0.48%)
Middle SDI		897318.6(422.55%)	-891.88(-0.42%)	540284.04(254.42%)	357926.44(168.55%)
East Asia		706739.68(507.78%)	1230.98(0.88%)	381723.73(274.26%)	323784.97(232.64%)
Southeast Asia		166708.24(545.2%)	-214.38(-0.7%)	89219.67(291.78%)	77702.96(254.12%)
Oceania		1934.79(219.33%)	-17.59(-1.99%)	1487.01(168.57%)	465.37(52.75%)
Central Asia		27581.46(85.93%)	954.93(2.97%)	24646.15(76.78%)	1980.37(6.17%)

Location	Overall difference	Aging	Population	Epidemiological change
Central Europe	165571.02(90.23%)	2729.21(1.49%)	108614.42(59.19%)	54227.4(29.55%)
Eastern Europe	230626.83(83.58%)	-1802.62(-0.65%)	103882.21(37.65%)	128547.23(46.58%)
High-income Asia Pacific	122131.69(174.71%)	6603.97(9.45%)	97608.32(139.63%)	17919.41(25.63%)
Australasia	35716.14(145.48%)	720.82(2.94%)	32022.84(130.44%)	2972.48(12.11%)
Western Europe	348724.2(56.68%)	19306.16(3.14%)	339727(55.22%)	-10308.96(-1.68%)
Southern Latin America	59740.36(111.02%)	162.11(0.3%)	51037.62(94.85%)	8540.63(15.87%)
High-income North America	437546.59(101.36%)	-1395.93(-0.32%)	403089.24(93.38%)	35853.29(8.31%)
Caribbean	29097.72(239.27%)	-71.03(-0.58%)	17451.85(143.51%)	11716.9(96.35%)
Andean Latin America	34618.15(359.99%)	-75.99(-0.79%)	24596.41(255.77%)	10097.73(105%)
Central Latin America	152768.38(388.15%)	-446.76(-1.14%)	109957.64(279.37%)	43257.5(109.91%)
Tropical Latin America	145754.54(346.32%)	-400.25(-0.95%)	104595.9(248.53%)	41558.89(98.75%)
North Africa and Middle East	266672.51(389.94%)	-146.35(-0.21%)	163231.02(238.68%)	103587.84(151.47%)
South Asia	215103.77(630.63%)	-1046.3(-3.07%)	109684.84(321.57%)	106465.23(312.13%)
Central Sub-Saharan Africa	12589.88(385.55%)	-65.15(-2%)	6704.83(205.33%)	5950.2(182.22%)
Eastern Sub-Saharan Africa	35653.37(302.93%)	-166.39(-1.41%)	19957.48(169.57%)	15862.28(134.78%)
Southern Sub-Saharan Africa	43251.82(384.39%)	-63.07(-0.56%)	21213.93(188.53%)	22100.97(196.42%)
Western Sub-Saharan Africa	53882.29(318.07%)	-85.79(-0.51%)	28259.69(166.82%)	25708.39(151.76%)

Table S13. Slope index of inequality and concentration index in global ASDR of 11 cancer subtypes in 1990 and 2021.

Sex	Cause	Year	Slope index of inequality	Concentration index
Female	Breast cancer	1990	239.24 (209.91 to 268.58)	0.3652 (0.3182 to 0.4122)
Female	Breast cancer	2021	141.23 (102.14 to 180.33)	0.1732 (0.1259 to 0.2206)
Both	Colon and rectum cancer	1990	262.09 (236.91 to 287.26)	0.4038 (0.3615 to 0.4461)
Both	Colon and rectum cancer	2021	220.55 (193.09 to 248.02)	0.2688 (0.2287 to 0.3089)
Both	Gallbladder and biliary tract cancer	1990	42.36 (35.62 to 49.10)	0.3035 (0.2342 to 0.3728)
Both	Gallbladder and biliary tract cancer	2021	23.54 (17.60 to 29.47)	0.1174 (0.0679 to 0.1670)
Both	Kidney cancer	1990	71.73 (63.39 to 80.08)	0.4550 (0.4032 to 0.5068)
Both	Kidney cancer	2021	83.75 (73.25 to 94.25)	0.3515 (0.2943 to 0.4088)
Both	Leukemia	1990	54.82 (48.10 to 61.54)	0.3140 (0.2759 to 0.3521)
Both	Leukemia	2021	44.41 (38.15 to 50.67)	0.2387 (0.1959 to 0.2814)
Both	Liver cancer	1990	20.57 (4.62 to 36.52)	0.1759 (0.0916 to 0.2601)
Both	Liver cancer	2021	29.13 (10.83 to 47.42)	0.1554 (0.0824 to 0.2284)
Both	Multiple myeloma	1990	23.62 (20.43 to 26.80)	0.5000 (0.4408 to 0.5592)
Both	Multiple myeloma	2021	28.11 (24.51 to 31.70)	0.3310 (0.2707 to 0.3913)
Both	Non-Hodgkin lymphoma	1990	20.05 (16.35 to 23.75)	0.3789 (0.3258 to 0.4321)
Both	Non-Hodgkin lymphoma	2021	19.21 (15.42 to 22.99)	0.2421 (0.1969 to 0.2873)
Female	Ovarian cancer	1990	76.36 (66.94 to 85.79)	0.4343 (0.3822 to 0.4865)
Female	Ovarian cancer	2021	66.20 (55.96 to 76.44)	0.2370 (0.1825 to 0.2916)
Both	Thyroid cancer	1990	9.30 (7.75 to 10.85)	0.2094 (0.1733 to 0.2455)
Both	Thyroid cancer	2021	3.82 (1.98 to 5.66)	0.0469 (0.0113 to 0.0826)
Female	Uterine cancer	1990	116.37 (88.77 to 143.97)	0.2876 (0.2354 to 0.3398)
Female	Uterine cancer	2021	80.69 (45.18 to 116.21)	0.2455 (0.1842 to 0.3067)

Table S14. Frontier analysis based on SDI and total cancer related ASDR attributable to high BMI among older people in 204 countries and territories in 2021.

Location	SDI	Frontier DALYs	Effective difference	Effective difference rank (Age-standardized DALYs rank)
Afghanistan	0.337199998	32.02	435.05	75 (75)
Albania	0.706849791	32.22	512.99	87 (87)
Algeria	0.659500924	31.87	279.26	46 (46)
American Samoa	0.723727533	31.57	974.11	173 (173)
Andorra	0.869444113	32.03	671.29	122 (122)
Angola	0.453721949	32.12	209.49	30 (30)
Antigua and Barbuda	0.749886887	31.62	812.01	153 (152)
Argentina	0.723122973	31.77	988.5	176 (176)
Armenia	0.701833194	32.16	907.41	166 (166)
Australia	0.844252814	31.66	809.75	151 (151)
Austria	0.853837004	32	657.87	115 (115)
Azerbaijan	0.694851274	31.9	601.13	103 (103)
Bahamas	0.805020668	31.73	990.68	177 (177)
Bahrain	0.753043204	32.1	923.88	168 (168)
Bangladesh	0.492420885	32.15	34.85	1 (1)
Barbados	0.746748764	31.97	1160.75	190 (190)
Belarus	0.784484711	31.5	981.93	174 (174)
Belgium	0.853654016	32.51	676.63	125 (125)
Belize	0.610229002	32.01	549.52	95 (95)
Benin	0.373486574	32.22	263.87	40 (40)
Bermuda	0.821365422	32.11	888.79	161 (161)
Bhutan	0.473062378	32.09	190.93	27 (27)

Location	SDI	Frontier DALYs	Effective difference	Effective difference rank (Age-standardized DALYs rank)
Bolivia (Plurinational State of)	0.599010799	32.06	743.58	140 (140)
Bosnia and Herzegovina	0.723077893	31.81	901.16	164 (164)
Botswana	0.642721629	31.82	545.34	93 (93)
Brazil	0.653043887	32.39	547.73	94 (94)
Brunei Darussalam	0.810234367	32.27	532.58	92 (92)
Bulgaria	0.768150939	32.01	1117.96	187 (187)
Burkina Faso	0.285118402	32.05	77.67	6 (4)
Burundi	0.289374365	31.81	87.02	8 (6)
Cabo Verde	0.533534539	31.79	416.85	72 (71)
Cambodia	0.473621491	32.2	150.47	19 (18)
Cameroon	0.479691223	32.27	510.44	86 (86)
Canada	0.87317068	32.15	765.76	147 (147)
Central African Republic	0.30916769	32.09	183.19	25 (25)
Chad	0.240436019	37.75	158.65	22 (23)
Chile	0.771514716	31.95	902.96	165 (165)
China	0.72162976	31.56	266.66	42 (42)
Colombia	0.655442913	32.24	517.88	90 (90)
Comoros	0.475978688	32.13	313.9	50 (50)
Congo	0.583075236	32.33	401.18	68 (68)
Cook Islands	0.779109955	32.09	688.55	129 (129)
Costa Rica	0.700340477	32.25	752.37	142 (142)
Coted'Ivoire	0.425941883	32.14	213.42	32 (32)
Croatia	0.798341027	32.32	1169.64	191 (191)

Location	SDI	Frontier DALYs	Effective difference	Effective difference rank (Age-standardized DALYs rank)
Cuba	0.668729864	32.16	672.04	123 (123)
Cyprus	0.835630545	32.2	620.56	112 (112)
Czechia	0.828450433	32.07	1201.63	195 (195)
Democratic People's Republic of Korea	0.569854634	31.7	163.89	23 (22)
Democratic Republic of the Congo	0.383179849	31.99	208.22	29 (29)
Denmark	0.896424204	32.4	696.08	130 (130)
Djibouti	0.487958371	32.36	132.14	12 (11)
Dominica	0.746967185	31.56	1041.9	182 (182)
Dominican Republic	0.619388201	31.96	338.14	57 (57)
Ecuador	0.661017053	32.03	620.33	111 (111)
Egypt	0.606787094	32.34	1188.2	193 (193)
El Salvador	0.563775188	32.27	504.27	85 (85)
Equatorial Guinea	0.657857456	32.28	460.35	80 (80)
Eritrea	0.403863943	31.9	137.9	14 (13)
Estonia	0.844917787	32.14	1017.96	181 (181)
Eswatini	0.585459713	32.25	1327.68	201 (201)
Ethiopia	0.358823295	32.32	145.98	17 (16)
Fiji	0.675051631	32.17	825.61	154 (154)
Finland	0.859831368	32.28	705.34	131 (131)
France	0.838364875	32.29	716.07	132 (132)
Gabon	0.634691393	32.26	682.42	127 (127)
Gambia	0.40971416	32.15	368.62	63 (63)
Georgia	0.732473604	31.71	893.98	163 (163)

Location	SDI	Frontier DALYs	Effective difference	Effective difference rank (Age-standardized DALYs rank)
Germany	0.902957091	31.79	783.63	148 (148)
Ghana	0.56493039	32.27	332.71	54 (54)
Greece	0.791854408	32.38	806.77	150 (150)
Greenland	0.826210336	32.42	918.89	167 (167)
Grenada	0.668993028	31.63	853.24	158 (158)
Guam	0.803982203	32.28	416.67	71 (72)
Guatemala	0.539972424	31.72	428.51	74 (74)
Guinea	0.336401293	31.65	228.87	35 (35)
Guinea-Bissau	0.353109621	32.19	300.6	49 (49)
Guyana	0.650812335	32.12	580.79	98 (98)
Haiti	0.448278285	32.16	252.63	38 (38)
Honduras	0.513037248	32.03	606.58	105 (105)
Hungary	0.790754768	32.34	1253.48	199 (199)
Iceland	0.87636168	31.73	733.55	137 (136)
India	0.575401649	32.15	91.29	9 (7)
Indonesia	0.656868336	32.17	147.61	18 (17)
Iran (Islamic Republic of)	0.697207398	32.32	383.76	64 (64)
Iraq	0.662626231	32.18	554.57	96 (96)
Ireland	0.87375385	32.07	679.73	126 (126)
Israel	0.809011652	32	674.75	124 (124)
Italy	0.805773534	32.06	686.99	128 (128)
Jamaica	0.683263064	32.14	811.94	152 (153)
Japan	0.871241813	31.77	280.11	47 (47)

Location	SDI	Frontier DALYs	Effective difference	Effective difference rank (Age-standardized DALYs rank)
Jordan	0.725307227	32.18	659.79	116 (116)
Kazakhstan	0.725144495	32.18	611.9	108 (108)
Kenya	0.523768077	32.04	219.37	34 (34)
Kiribati	0.527186583	31.82	586.01	100 (100)
Kuwait	0.846651055	32.2	595.19	102 (102)
Kyrgyzstan	0.603979328	31.76	520.21	91 (91)
Lao People's Democratic Republic	0.489136091	32.39	167.67	24 (24)
Latvia	0.830663516	31.65	1125.21	188 (188)
Lebanon	0.744746351	32.13	759.95	146 (146)
Lesotho	0.510393066	31.68	886.12	160 (160)
Liberia	0.352442452	32.03	442.51	76 (76)
Libya	0.725771399	31.76	1117.71	186 (186)
Lithuania	0.856484049	32.19	1091.81	185 (185)
Luxembourg	0.884428955	32.36	729.87	134 (134)
Madagascar	0.400246943	32.49	153.42	20 (19)
Malawi	0.384553634	32.18	154.79	21 (20)
Malaysia	0.742523828	32.13	450.52	79 (79)
Maldives	0.650886627	31.79	113.55	11 (10)
Mali	0.268579941	32.12	217.07	33 (33)
Malta	0.801585034	31.83	666.04	117 (117)
Marshall Islands	0.574091128	31.68	667.13	119 (119)
Mauritania	0.4989451	32.23	583.62	99 (99)
Mauritius	0.718260446	31.87	443.05	77 (77)

Location	SDI	Frontier DALYs	Effective difference	Effective difference rank (Age-standardized DALYs rank)
Mexico	0.664575304	31.88	608.46	107 (106)
Micronesia (Federated States of)	0.587534967	32.22	751.57	141 (141)
Monaco	0.908262831	32.08	1189.61	194 (194)
Mongolia	0.617621565	32.27	1444.51	203 (203)
Montenegro	0.795800584	32.43	1147.35	189 (189)
Morocco	0.562698301	32.23	245.42	37 (37)
Mozambique	0.326462614	31.79	277.34	45 (45)
Myanmar	0.53390084	32.13	142.44	16 (15)
Namibia	0.617564872	32.11	448.96	78 (78)
Nauru	0.625177834	32.24	1010.75	178 (178)
Nepal	0.433174635	32.32	56.88	3 (3)
Netherlands	0.888464256	32.18	736.38	138 (138)
New Zealand	0.849442499	32.39	837.16	156 (156)
Nicaragua	0.523958472	31.78	419.55	73 (73)
Niger	0.168072774	84.29	57.59	4 (9)
Nigeria	0.503390833	32.34	324.04	53 (53)
Niue	0.72622205	31.98	741.09	139 (139)
North Macedonia	0.750629703	32.17	1067.7	183 (183)
Northern Mariana Islands	0.771535213	32.03	986.46	175 (175)
Norway	0.91613281	32.32	586.14	101 (101)
Oman	0.773391602	32.23	356.62	61 (61)
Pakistan	0.504028689	32.21	341.81	58 (58)
Palau	0.754046931	32.02	788.18	149 (149)

Location	SDI	Frontier DALYs	Effective difference	Effective difference rank (Age-standardized DALYs rank)
Palestine	0.631011665	32.16	1011.45	179 (179)
Panama	0.708864828	31.57	718.59	133 (133)
Papua New Guinea	0.417797443	32.15	133.04	13 (12)
Paraguay	0.635718099	32.04	604.59	104 (104)
Peru	0.662054037	32.16	516.99	88 (88)
Philippines	0.651219329	32.14	257.72	39 (39)
Poland	0.812042809	31.56	1172.84	192 (192)
Portugal	0.744151851	32.24	666.31	118 (118)
Puerto Rico	0.825525847	32.14	730.61	135 (135)
Qatar	0.846860584	32.36	1324.1	200 (200)
Republic of Korea	0.886675267	32.24	274.26	43 (43)
Republic of Moldova	0.732214875	32.18	953.15	171 (171)
Romania	0.768453864	32.35	946.25	170 (170)
Russian Federation	0.808536005	32.35	1074.26	184 (184)
Rwanda	0.435588706	32.4	198.33	28 (28)
Saint Kitts and Nevis	0.754987055	32.02	925.01	169 (169)
Saint Lucia	0.672509735	31.94	499.61	84 (84)
Saint Vincent and the Grenadines	0.637195963	32.18	475.05	82 (83)
Samoa	0.593392769	32	733.45	136 (137)
San Marino	0.888005474	31.73	475.36	83 (82)
Sao Tome and Principe	0.505413747	31.9	367.44	62 (62)
Saudi Arabia	0.815143493	31.72	668.09	121 (120)
Senegal	0.408054193	32.09	242.78	36 (36)

Location	SDI	Frontier DALYs	Effective difference	Effective difference rank (Age-standardized DALYs rank)
Serbia	0.792416294	31.74	1205.34	196 (196)
Seychelles	0.730150775	32.31	648.39	114 (114)
Sierra Leone	0.358665881	32.32	183.56	26 (26)
Singapore	0.856097766	31.66	320.73	52 (52)
Slovakia	0.81061053	32.19	1370.57	202 (202)
Slovenia	0.842430731	32.41	1015.16	180 (180)
Solomon Islands	0.429360316	31.71	315.95	51 (51)
Somalia	0.077688109	112.17	76	5 (21)
South Africa	0.679626598	31.73	756.72	144 (144)
South Sudan	0.278371125	32.04	92.16	10 (8)
Spain	0.769283698	32.38	829.48	155 (155)
Sri Lanka	0.701534935	31.61	139.14	15 (14)
Sudan	0.541949735	32.01	333.64	55 (55)
Suriname	0.633665739	32.07	391.29	65 (65)
Sweden	0.886880299	32.32	608.17	106 (107)
Switzerland	0.933059111	32.24	468.19	81 (81)
Syrian Arab Republic	0.623004075	31.52	570.81	97 (97)
Taiwan (Province of China)	0.874747053	32.41	517.42	89 (89)
Tajikistan	0.541511187	32.4	300	48 (48)
Thailand	0.682547933	31.69	392.6	66 (66)
Timor-Leste	0.444667619	31.7	38.04	2 (2)
Togo	0.408533695	32.09	264.16	41 (41)
Tokelau	0.686425621	32.14	619.47	110 (110)

Location	SDI	Frontier DALYs	Effective difference	Effective difference rank (Age-standardized DALYs rank)
Tonga	0.626349936	31.92	1215.84	198 (198)
Trinidad and Tobago	0.768763254	32.02	753.39	143 (143)
Tunisia	0.682432216	31.6	405.7	69 (69)
Turkey	0.712692673	31.98	758.48	145 (145)
Turkmenistan	0.682160776	31.65	393.46	67 (67)
Tuvalu	0.576620529	32.17	618.54	109 (109)
Uganda	0.423261181	32.27	276.58	44 (44)
Ukraine	0.760773913	32.22	847.35	157 (157)
United Arab Emirates	0.849317734	32.22	1581.21	204 (204)
United Kingdom	0.859000182	32.15	889.12	162 (162)
United Republic of Tanzania	0.446568273	32.06	353.75	60 (60)
United States Virgin Islands	0.821830853	32.23	631.75	113 (113)
United States of America	0.862448354	32.23	968.97	172 (172)
Uruguay	0.719283445	32.08	1212.2	197 (197)
Uzbekistan	0.662621694	31.95	350.65	59 (59)
Vanuatu	0.473100706	31.79	334.88	56 (56)
Venezuela (Bolivarian Republic of)	0.596513059	32	667.95	120 (121)
Viet Nam	0.627933721	32.02	82.8	7 (5)
Yemen	0.450376375	31.6	212.31	31 (31)
Zambia	0.505948954	32.19	413.17	70 (70)
Zimbabwe	0.473819486	31.96	858.73	159 (159)

Figure S1 Joinpoint regression analysis of ASDR for total cancer attributable to high BMI among older people from 1990 to 2021 across 21 GBD regional levels.

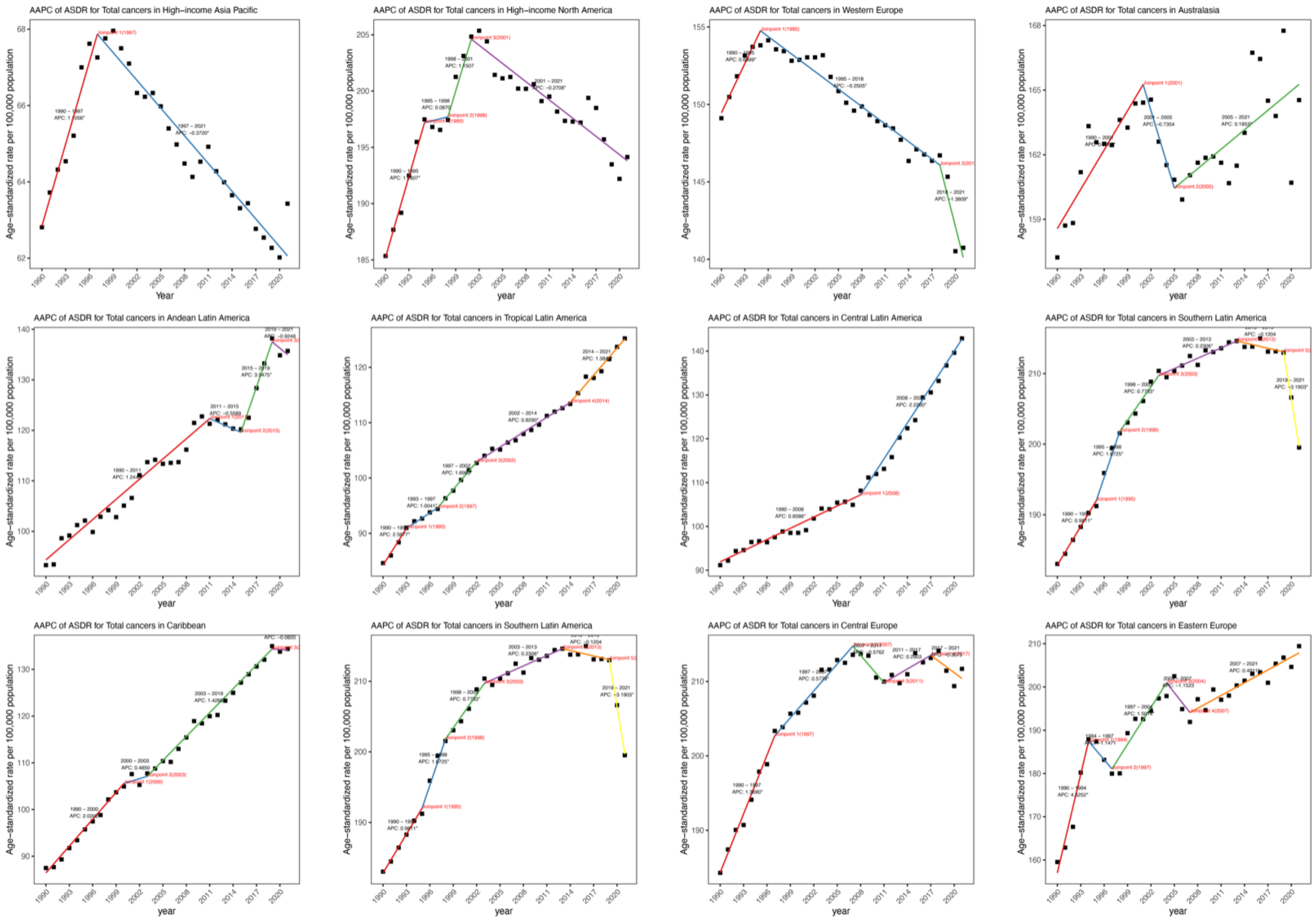

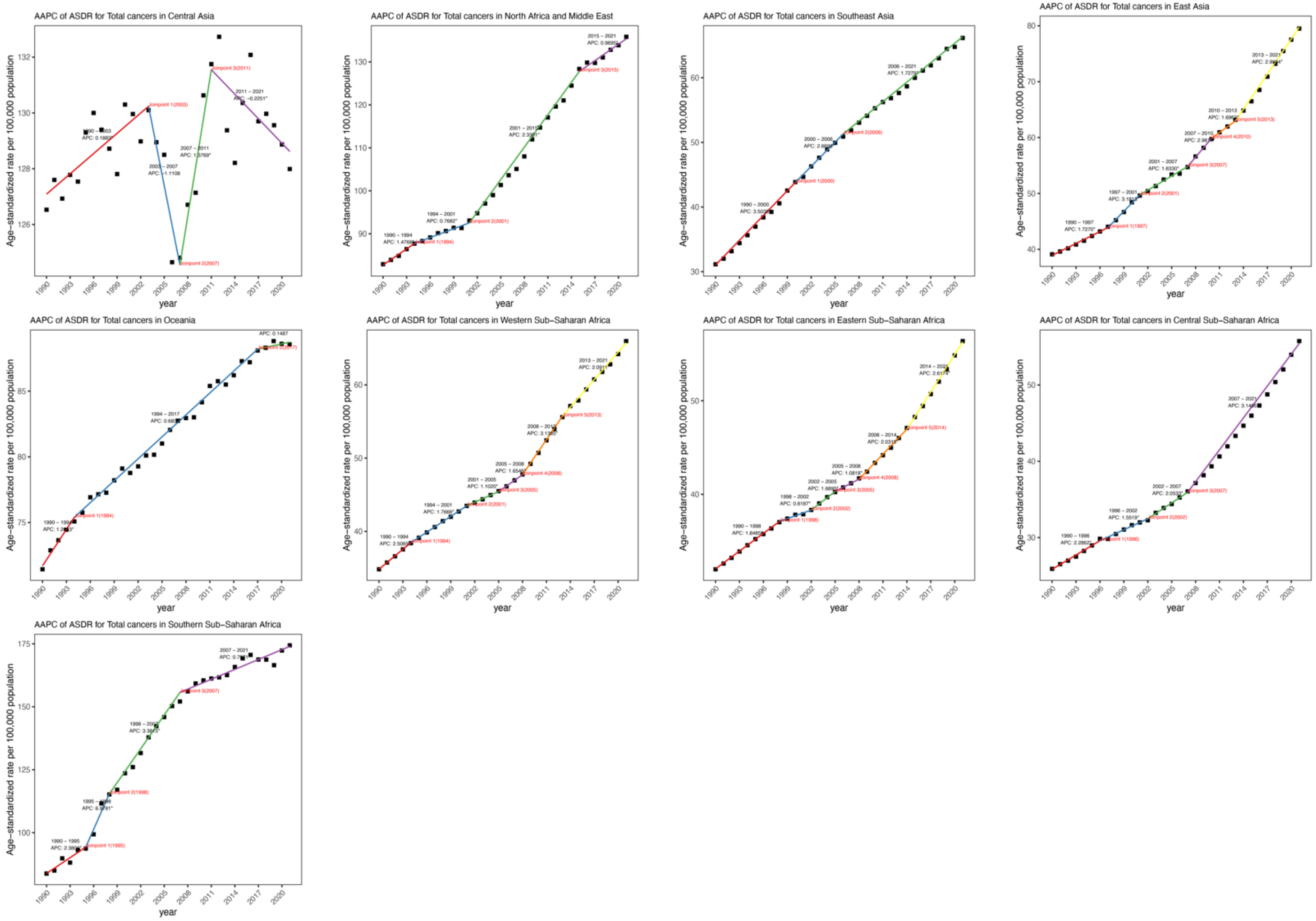
AAPC of ASDR for Total cancers in Central Asia
AAPC of ASDR for Total cancers in North Africa and Middle East
AAPC of ASDR for Total cancers in Southeast Asia
AAPC of ASDR for Total cancers in East Asia
AAPC of ASDR for Total cancers in Oceania
AAPC of ASDR for Total cancers in Western Sub−Saharan Africa
AAPC of ASDR for Total cancers in Eastern Sub−Saharan Africa
AAPC of ASDR for Total cancers in Central Sub−Saharan Africa
AAPC of ASDR for Total cancers in Southern Sub−Saharan Africa
Age−standardized rate per 100,000 population
year
